# Supplementary figures and images for: The use of brain-machine interface, motor imagery, and action observation in the rehabilitation of individuals with Parkinson’s disease: A protocol study for a randomized clinical trial
Source: PLoS One. 2025 Apr 7;20(4):e0315148. doi: 10.1371/journal.pone.0315148 (PMC11975075; doi:10.1371/journal.pone.0315148)

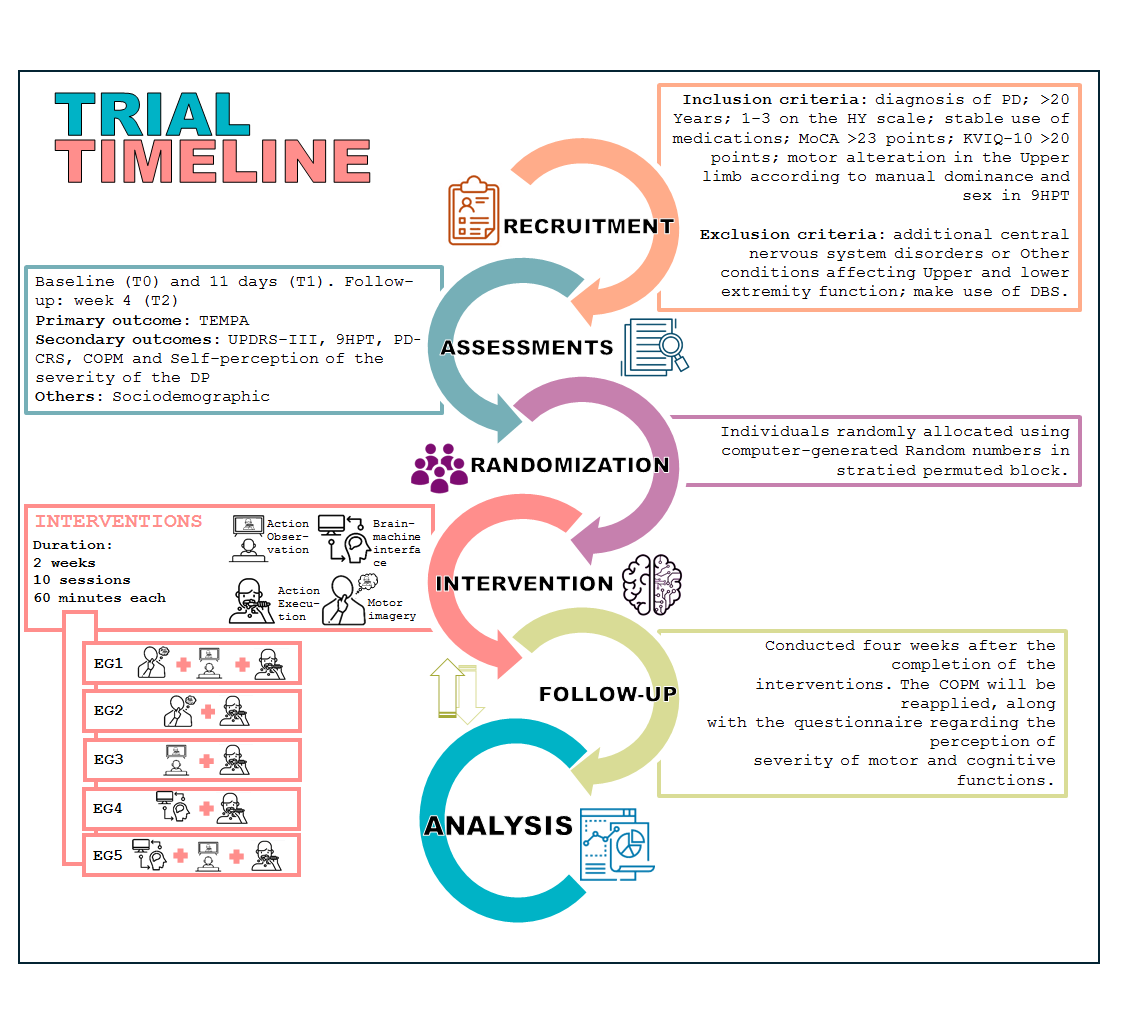

Supplement: S1 Graphycal Abstract — (TIF) [file pone.0315148.s002.tif]
