# Supplementary material for: The use of brain-machine interface, motor imagery, and action observation in the rehabilitation of individuals with Parkinson’s disease: A protocol study for a randomized clinical trial
Source: PLoS One. 2025 Apr 7;20(4):e0315148. doi: 10.1371/journal.pone.0315148 (PMC11975075; doi:10.1371/journal.pone.0315148)
Supplement: S1 File — (PDF) [file pone.0315148.s006.pdf]

PARECER CONSUBSTANCIADO DO CEP

DADOS DO PROJETO DE PESQUISA

Título da Pesquisa: EFEITOS DA IMAGINAÇÃO MOTORA E DA OBSERVAÇÃO DA AÇÃO NAS ALTERAÇÕES MOTORAS EM MEMBROS SUPERIORES E NAS ALTERAÇÕES COGNITIVAS NA DOENÇA DE PARKINSON: ENSAIO CLÍNICO RANDOMIZADO

Pesquisador: Fernanda Cechetti

Área Temática:

Versão: 2

CAAE: 61710822.0.0000.5345

Instituição Proponente: Universidade Federal de Ciências da Saúde de Porto Alegre

Patrocinador Principal: Financiamento Próprio

DADOS DO PARECER

Número do Parecer: 5.700.603

Apresentação do Projeto:

As informações elencadas neste campo foram retiradas do arquivo Informações Básicas da pesquisa (PB_INFORMAÇÕES_BÁSICAS_DO_PROJETO_1998037.pdf) de 21/09/2022. A doença de Parkinson é degenerativa, progressiva e crônica. Considerada potencialmente incapacitante, em vista das alterações motoras, como bradicinesia, rigidez e tremor nos membros superiores, e alterações não-motoras, como as cognitivas envolvendo dificuldades de atenção e concentração e de memória. Assim, tem-se apostado nas modalidades de neuroreabilitação, como a imaginação motora e a observação da ação. O objetivo da pesquisa é investigar os efeitos da imaginação motora e da observação da ação nas alterações motoras em membros superiores e nas alterações cognitivas na doença de Parkinson. Trata-se de um estudo do tipo ensaio clínico controlado randomizado. A população do estudo envolve pessoas com doença de Parkinson no estágio 1-3 na escala Hoehn e Yahr, na faixa etária entre 20 até 59 anos, e deve estar fazer uso estável de medicamentos, não apresentar alteração cognitiva com risco de demência, e ser capaz de imaginar atividades motoras, e apresentar alteração motora em membro superior. Os grupos do estudo serão: a

imaginação motora, observação da ação e execução motora; b) imaginação motora e execução motora; c) observação da ação e execução motora; d) imaginação motora e execução motora e exoesqueleto; e) observação da ação e execução motora e exoesqueleto. As intervenções de

Endereço: Rua Sarmento Leite, 245, prédio 03, sala 605
Bairro: Sarmento **CEP:** 90.050-170
UF: RS **Município:** PORTO ALEGRE
Telefone: (51)3303-8804 **E-mail:** cep@ufcspa.edu.br

todos grupos serão de uma abordagem intensivista de 10 sessões contínuas, com intervalo de dois dias na metade da intervenção, totalizando duas semanas, sendo cada sessão de 60 minutos por dia. As etapas para coleta de dados do estudo envolverão o pré-teste, as intervenções, o pós-teste imediato e o teste após um período de quatro semanas sem intervenção. Os instrumentos que serão usados para as avaliações: a) parte da Escala de Avaliação da Doença de Parkinson Unificada (UPDRS-III); b) Test D'évaluation Des Membres Supérieurs Des Personnes Âgées (TEMPA); c) 9-Hole Peg Test para avaliar a função da extremidade superior; d) Escala de Avaliação Cognitiva da Doença de Parkinson; e) Medida Canadense de Desempenho Ocupacional para identificar o desempenho e a satisfação na realização das atividades-problema que considera importante nas áreas de autocuidado, produtividade e lazer.

Objetivo da Pesquisa:

Objetivo Primário:

Investigar os efeitos da imaginação motora e da observação da ação nas alterações motoras em membros superiores e nas alterações cognitivas na doença de Parkinson.

Objetivo Secundário:

1. Analisar os efeitos isolados da imaginação motora e da observação da ação nas alterações motoras em membros superiores, através do Test d'Évaluation des Membres Supérieurs of Personnes Âgées (TEMPA) e do 9-Hole Peg Test (9HPT) e alterações cognitivas, através do Parkinson's Disease-Cognitive Rating Scale (PD-CRS).
2. Analisar os efeitos da combinação da imaginação motora e da observação da ação nas alterações motoras em membros superiores, através do Test d'Évaluation des Membres Supérieurs of Personnes Âgées (TEMPA) e do 9-Hole Peg Test (9HPT) e alterações cognitivas, através do Parkinson's Disease-Cognitive Rating Scale (PD-CRS).
3. Comparar os efeitos entre a imaginação motora e execução da ação com a observação da ação e a execução da ação.
4. Comparar os efeitos entre o uso do exoesqueleto combinado com imaginação motora e execução da ação e com observação da ação e execução da ação.
5. Avaliar o reflexo do efeito da imaginação motora e da observação da ação no desempenho ocupacional na realização de atividades relacionadas com as áreas de autocuidado, produtividade e lazer, através da Medida Canadense de Desempenho Ocupacional (COPM).

Avaliação dos Riscos e Benefícios:

Riscos:

A pesquisa envolve riscos mínimos aos participantes. O uso dos instrumentos pode causar algum desconforto ou constrangimento ou cansaço durante a aplicabilidade, bem como durante a realização das intervenções. Se acontecer qualquer tipo de situação que cause risco ao participante, o mesmo pode suspender temporariamente a participação na pesquisa, ou desistir da mesma em qualquer etapa, sem provocar-lhe algum prejuízo e serão acompanhados até a resolução.

Benefícios:

Como benefícios, a pesquisa pretende investigar a associação dos efeitos da imaginação motora e da observação da ação nas alterações motoras em membros superiores e cognitivas da DP. Caso as hipóteses do estudo aponte para uma possibilidade positiva de ambas as intervenções, tem-se uma condição de melhora de sintomas motores e cognitivos, beneficiando os participantes na condição de saúde. Como reflexo, pode-se melhorar o

desempenho ocupacional em relação a realização das atividades diárias. O participante também receberá um parecer dos resultados dos testes, tendo um conhecimento de suas condições motoras e cognitivas.

Comentários e Considerações sobre a Pesquisa:

Trata-se de um estudo do tipo ensaio clínico controlado randomizado, simples-cego, uma vez que os participantes serão distribuídos aleatoriamente em grupos para aplicação das intervenções por um período de tempo, analisando posteriormente os desfechos do estudo. Os participantes da pesquisa serão formados por pessoas que apresentam diagnóstico de doença de Parkinson e estar no estágio 1-3 na escala Hoehn e Yahr, condizentes com incapacidade leve e moderada, com idade entre 20 anos até 59 anos.

Estudo de caráter acadêmico, como projeto de Doutorado do Programa de Pós-Graduação em Ciências da Reabilitação da UFCSPA, com início previsto para o 2° semestre de 2022 e encerramento no 2° semestre de 2024.

Considerações sobre os Termos de apresentação obrigatória:

Termos obrigatórios apresentados e aceitos.

Recomendações:

Iniciar coleta de dados somente após a aprovação do projeto junto ao CEP. Vide campo “Conclusões ou Pendências e Lista de Inadequações”.

Conclusões ou Pendências e Lista de Inadequações:

O projeto está adequado para ser desenvolvido, tendo seu término previsto para 12/2024.

Ressalta-se que cabe ao pesquisador responsável encaminhar os relatórios parciais e final da pesquisa, por meio da Plataforma Brasil, via notificação do tipo “relatório” para que sejam devidamente apreciadas no CEP, conforme Norma Operacional CNS nº 001/12, item XI.2.d.

Considerações Finais a critério do CEP:

De acordo com o parecer do Relator.

Este parecer foi elaborado baseado nos documentos abaixo relacionados:

Tipo Documento	Arquivo	Postagem	Autor	Situação
Informações Básicas do Projeto	PB_INFORMAÇÕES_BÁSICAS_DO_PROJETO_1998037.pdf	21/09/2022 15:57:50		Aceito
Outros	cartazdivulgacao.pdf	21/09/2022 15:57:20	Fernanda Cechetti	Aceito
Outros	Cartaresposta.docx	21/09/2022 15:57:02	Fernanda Cechetti	Aceito
Projeto Detalhado / Brochura Investigador	projetorevisado.docx	21/09/2022 15:56:34	Fernanda Cechetti	Aceito
Outros	anexo1.pdf	17/08/2022 08:10:45	Fernanda Cechetti	Aceito
Outros	anexo5.doc	15/08/2022 14:51:22	Fernanda Cechetti	Aceito
Folha de Rosto	folhaDeRosto.pdf	15/08/2022 14:44:13	Fernanda Cechetti	Aceito
Outros	CurriculoRafael.pdf	11/08/2022 11:30:24	Fernanda Cechetti	Aceito
Outros	CurriculoFernanda.pdf	11/08/2022 11:29:13	Fernanda Cechetti	Aceito
TCLE / Termos de Assentimento / Justificativa de Ausência	TCLE.docx	11/08/2022 11:23:21	Fernanda Cechetti	Aceito
Outros	CurriculoTatiana.pdf	11/08/2022 11:23:06	Fernanda Cechetti	Aceito
Outros	CurriculoKatine.pdf	11/08/2022 11:22:48	Fernanda Cechetti	Aceito
Outros	CartaAnuenciaNeurogold.pdf	11/08/2022 11:22:20	Fernanda Cechetti	Aceito
Outros	cartadeanuenciaUFSM.pdf	11/08/2022 11:12:20	Fernanda Cechetti	Aceito
Projeto Detalhado / Brochura Investigador	ProjetoFinalCEP.pdf	11/08/2022 11:11:10	Fernanda Cechetti	Aceito

Situação do Parecer:
Aprovado

Necessita Apreciação da CONEP:
Não

PORTO ALEGRE, 14 de Outubro de 2022

Assinado por:
Fernanda Bordignon Nunes
(Coordenador(a))

Endereço: Rua Sarmento Leite, 245, prédio 03, sala 605
Bairro: Sarmento **CEP:** 90.050-170
UF: RS **Município:** PORTO ALEGRE
Telefone: (51)3303-8804 **E-mail:** cep@ufcspa.edu.br
