# Supplementary material for: The use of brain-machine interface, motor imagery, and action observation in the rehabilitation of individuals with Parkinson’s disease: A protocol study for a randomized clinical trial
Source: PLoS One. 2025 Apr 7;20(4):e0315148. doi: 10.1371/journal.pone.0315148 (PMC11975075; doi:10.1371/journal.pone.0315148)
Supplement: S2 File — (PDF) [file pone.0315148.s007.pdf]

CONSUBSTANTIATED OPINION OF THE CEP

RESEARCH PROJECT DATA

Research Title: EFFECTS OF MOTOR IMAGERY, AND ACTION OBSERVATION ON MOTOR CHANGES IN UPPER LIMBS AND COGNITIVE CHANGES IN PARKINSON'S DISEASE: RANDOMIZED CLINICAL TRIAL
Researcher: Fernanda Cechetti

Thematic Area:

Version: 2

CAAE: 61710822.0.0000.5345

Proposing Institution: Federel University of Health Sciences of Porto Alegre

Main Sponsor: Own Financing

OPINION DATA

Opinion Number: 5.700.603

Project Presentation:

The information outlined in this section was taken from the Basic Information document of the research (PB_BASIC_INFORMATION_OF_THE_PROJECT_1998037.pdf) dated 09/21/2022. Parkinson's disease is degenerative, progressive, and chronic. It is considered potentially disabling due to motor alterations, such as bradykinesia, rigidity, and tremor in the upper limbs, as well as non-motor changes, including cognitive difficulties involving attention, concentration, and memory. Consequently, there has been a focus on neurorehabilitation modalities, such as motor imagery and action observation. The objective of the research is to investigate the effects of motor imagery and action observation on motor alterations in the upper limbs and cognitive changes in Parkinson's disease. This is a randomized controlled clinical trial. The study population includes individuals with Parkinson's disease in stages 1-3 on the Hoehn and Yahr scale, aged between 20 and 59 years, who are on stable medication, do not show cognitive impairment with a risk of dementia, are capable of imagining motor activities, and present motor alterations in the upper limbs. The study groups will be: a) motor imagery, action observation, and motor execution; b) motor imagery and motor execution; c) action observation and motor execution; d) motor imagery, motor execution, and exoskeleton; e) action observation, motor execution, and exoskeleton. The interventions for all groups will follow an intensive approach consisting of 10 continuous sessions, with a two-day break in the middle of the intervention, totaling two weeks, with each session lasting 60 minutes per day. The data collection stages for the study will include a pre-test, the interventions, an immediate post-test, and a test after a four-week period without intervention. The instruments that will be used for evaluations include: a) part of the Unified Parkinson's Disease Rating Scale (UPDRS-III); b) Test D'évaluation Des Membres Supérieurs Des Personnes Âgées (TEMPA); c) 9-Hole Peg Test to assess upper extremity function; d) Cognitive

Assessment Scale for Parkinson's Disease; e) Canadian Occupational Performance Measure to identify performance and satisfaction in carrying out important problem-related activities in the areas of self-care, productivity, and leisure.

Research Objective:

Primary Objective:

To investigate the effects of motor imagery and action observation on motor alterations in the upper limbs and cognitive changes in Parkinson's disease.

Secondary Objectives:

1. To analyze the isolated effects of motor imagery and action observation on motor alterations in the upper limbs, using the Test d'Évaluation des Membres Supérieurs des Personnes Âgées (TEMPA) and the 9-Hole Peg Test (9HPT), as well as cognitive changes through the Parkinson's Disease-Cognitive Rating Scale (PD-CRS).
2. To analyze the effects of the combination of motor imagery and action observation on motor alterations in the upper limbs, using the TEMPA and the 9-Hole Peg Test (9HPT), and cognitive changes through the PD-CRS.
3. To compare the effects between motor imagery and action execution with action observation and action execution.
4. To compare the effects of using an exoskeleton combined with motor imagery and action execution against action observation and action execution.
5. To evaluate the impact of motor imagery and action observation on occupational performance in carrying out activities related to self-care, productivity, and leisure, using the Canadian Occupational Performance Measure (COPM).

Assessment of Risks and Benefits:

Risks:

The research involves minimal risks to participants. The use of the instruments may cause some discomfort, embarrassment, or fatigue during application, as well as during the interventions. If any situation arises that poses a risk to the participant, they may temporarily suspend their participation in the study or withdraw at any stage without incurring any disadvantage, and they will be monitored until the issue is resolved.

Benefits: The research aims to investigate the association between the effects of motor imagery and action observation on motor alterations in the upper limbs and cognitive changes in Parkinson's disease (PD). If the study hypotheses indicate a positive outcome for both interventions, it may lead to improvements in motor and cognitive symptoms, benefiting participants' health condition. Consequently, this could enhance occupational performance in daily activities. Participants will also receive feedback on their test results, gaining insights into their motor and cognitive conditions.

Comments and Considerations on the Research:

This is a randomized controlled clinical trial, single-blind study, as participants will be randomly assigned to groups for the application of interventions over a specified period, with outcomes analyzed afterward. The research participants will consist of individuals diagnosed with Parkinson's disease who are in stages 1-3 on the Hoehn and Yahr scale, corresponding to mild and moderate disability, aged between 20 and 59 years.

The study is of an academic nature, part of a doctoral project within the Graduate Program in Rehabilitation Sciences at UFCSPA, with a planned start in the second semester of 2022 and concluding in the second semester of 2024.

Considerations on the Mandatory Presentation Terms:

Mandatory terms have been presented and accepted.

Recommendations:

Data collection should begin only after the project has been approved by the Research Ethics Committee (CEP). See the section “Conclusions or Pending Issues and List of Inadequacies.”

Conclusions or Pending Issues and List of Inadequacies:

The project is deemed suitable for development, with an expected completion date of December 2024.

It is emphasized that it is the responsibility of the principal investigator to submit the partial and final reports of the research through the Plataforma Brasil, via a notification of the type “report,” so that they can be properly reviewed by the Research Ethics Committee (CEP), in accordance with Operational Norm CNS No. 001/12, item XI.2.d.

Final Considerations at the discretion of the CEP:

According to the opinion of the Rapporteur.

This opinion was prepared based on the documents listed below:

Document Typy	File	Post	Author	Situation
Basic Information of the project	PB_INFORMAÇÕES_BÁSICAS_DO_PROJETO_1998037.pdf	21/09/2022 15:57:50		Accepted
Others	cartazdivulgacao.pdf	21/09/2022 15:57:20	Fernanda Cechetti	Accepted
Others	Cartaresposta.docx	21/09/2022 15:57:02	Fernanda Cechetti	Accepted
Detailed desing / Researcher Brochure	projetorevisado.docx	21/09/2022 15:56:34	Fernanda Cechetti	Accepted
Others	anexo1.pdf	17/08/2022 08:10:45	Fernanda Cechetti	Accepted
Others	anexo5.doc	15/08/2022 14:51:22	Fernanda Cechetti	Accepted
Cover Page	folhaDeRosto.pdf	15/08/2022 14:44:13	Fernanda Cechetti	Accepted
Others	CurriculoRafael.pdf	11/08/2022 11:30:24	Fernanda Cechetti	Accepted

Others	CurriculoFernanda.pdf	11/08/2022 11:29:13	Fernanda Cechetti	Accepted
TCLE / Terms of Assent / Justification of absence	TCLE.docx	11/08/2022 11:23:21	Fernanda Cechetti	Accepted
Others	CurriculoTatiana.pdf	11/08/2022 11:23:06	Fernanda Cechetti	Accepted
Others	CurriculoKatine.pdf	11/08/2022 11:22:48	Fernanda Cechetti	Accepted
Others	CartaAnuenciaNeurogold.pdf	11/08/2022 11:22:20	Fernanda Cechetti	Accepted
Others	cartadeanuenciaUFSM.pdf	11/08/2022 11:12:20	Fernanda Cechetti	Accepted
Detailed desing / Researcher Brochure	ProjetoFinalCEP.pdf	11/08/2022 11:11:10	Fernanda Cechetti	Accepted

Status of the Opinion:
Approved

Needs CONEP Consideration:
No

PORTO ALEGRE, 14 de Outubro de 2022

Signed by: Fernanda Bordignon Nunes (Coordinator)

Endereço: Rua Sarmento Leite, 245, prédio 03, sala 605
Bairro: Sarmento **CEP:** 90.050-170
UF: RS **Município:** PORTO ALEGRE
Telefone: (51)3303-8804 **E-mail:** cep@ufcspa.edu.br

Endereço: Rua Sarmento Leite, 245, prédio 03, sala 605
Bairro: Sarmento **CEP:** 90.050-170
UF: RS **Município:** PORTO ALEGRE
Telefone: (51)3303-8804 **E-mail:** cep@ufcspa.edu.br
