# Supplementary material for: The use of brain-machine interface, motor imagery, and action observation in the rehabilitation of individuals with Parkinson’s disease: A protocol study for a randomized clinical trial
Source: PLoS One. 2025 Apr 7;20(4):e0315148. doi: 10.1371/journal.pone.0315148 (PMC11975075; doi:10.1371/journal.pone.0315148)
Supplement: S3 File — (PDF) [file pone.0315148.s008.pdf]

ClinicalTrials.gov PRS DRAFT Receipt (Working Version)
Last Update: 01/20/2023 10:06

ClinicalTrials.gov ID: NCT05696925

Study Identification

Unique Protocol ID: IM+OA+EX

Brief Title: Effects of Motor Imagery and Action Observation on Upper Limb Motor Chances and Cognitive Chances in Parkinson's Disease

Official Title: Effects of Motor Imagery and Action Observation on Upper Limb Motor Chances and Cognitive Chances in Parkinson's Disease: Randomized Clinical Trial

Secondary IDs:

Study Status

Record Verification: January 2023
ERROR : A record for an active study (Overall Recruitment Status is not Completed, Terminated or Withdrawn) must be reviewed, updated and verified at least once per year.

Overall Status: Not yet recruiting

Study Start: February 1, 2023 [Anticipated]
WARNING : Start Date February 1, 2023 should not be in the past for a study that is Not yet recruiting.
ERROR : Anticipated Start Date cannot be in the past.

Primary Completion: September 30, 2024 [Anticipated]
ERROR : Anticipated Primary Completion Date cannot be in the past.

Study Completion: September 30, 2026 [Anticipated]

Sponsor/Collaborators

Sponsor: Federal University of Health Science of Porto Alegre

Responsible Party: Principal Investigator
Investigator: Fernanda Cechetti [FCechetti]
Official Title: Clinical Professor, Ph.D Fernanda Cechetti
Affiliation: Federal University of Health Science of Porto Alegre

Collaborators:

Oversight

U.S. FDA-regulated Drug: No

U.S. FDA-regulated Device: No

U.S. FDA IND/IDE: No

Human Subjects Review: Board Status: Approved
Approval Number: 5.700.603
Board Name: omitê de Ética em Pesquisa da Fundação Faculdade Federal de Ciências Médicas de Porto Alegre (CEP-UFCSPA)
Board Affiliation: Fundação Faculdade Federal de Ciências Médicas de Porto Alegre (UFCSPA)
Phone: 55 (51)3303-8804
Email: cep@ufcspa.edu.br
Address:

Rua Sarmento Leite, 245, prédio 03, sala 605
Bairro: Sarmento CEP: 90.050-170

PORTO ALEGRE, RS, BRASIL

Data Monitoring: Yes

FDA Regulated Intervention: No

Study Description

Brief Summary: Parkinson's disease is degenerative, progressive, and chronic. It is considered potentially disabling, in view of the motor alterations, such as bradykinesia, rigidity, and tremor in the upper limbs, and non-motor alterations, such as cognitive ones involving attention, concentration and memory difficulties. Thus, neurorehabilitation modalities, such as motor imagery and action observation, have been used. The aim of this research is to investigate the effects of motor imagery and action observation on upper limb motor and cognitive changes in Parkinson's disease. This is a randomized controlled trial type study. The population of the study involves people with Parkinson's disease in stages 1-3 on the Hoehn and Yahr scale, aged 20 to 59 years, who must be on stable medication, have no cognitive impairment with risk of dementia, be able to imagine motor activities and have upper limb motor impairment. The study groups will be: a) motor imagery, observation of action and motor execution; b) motor imagery and motor execution; c) observation of action and motor execution; d) motor imagery and motor execution and exoskeleton; e) observation of action and motor execution and exoskeleton. The interventions of all groups will be an intensive approach of 10 continuous sessions, with a two-day break in the middle of the intervention, totaling two weeks, with each session lasting 40 minutes a day. The data collection steps for the study will involve the pre-test, the interventions, the immediate post-test, and the test after a four-week period without intervention. The instruments that will be used for the evaluations will be: a) part of the Unified Parkinson's Disease Rating Scale (UPDRS-III); b) Test D'évaluation Des Membres Supérieurs Des Personnes Âgées (TEMPA); c) 9-Hole Peg Test to assess upper extremity function; d) Parkinson's Disease Cognitive Assessment Scale; e) Canadian Occupational Performance Measure to identify performance and satisfaction in performing activities in the areas of self-care, productivity, and leisure.

Detailed Description: Parkinson's disease (PD) is degenerative, occurs slowly and progressively, is idiopathic, and involves several factors. In PD occurs the degeneration of dopamine-producing neurons (dopaminergic neurons) in the substantia nigra, within the basal ganglia. The activation of excitatory neurons then occurs, manifesting itself through the dysregulation of motor control.

PD is characterized by non-motor and motor symptoms, being causes of negative implications in quality of life, reflecting directly in several daily situations of the person with PD. Non-motor symptoms are considered as additional symptoms and are also frequent, having the prevalence of dementia and mild cognitive impairment. The most observed cognitive problems in PD are

in the following domains: concentration and attention, working memory, as well as difficulties with calculations and spatial orientation activities, and executive functions. The non-motor symptoms have a negative impact on occupational performance, and may present difficulties in activities such as driving, shopping, household maintenance; as well as self-care: dressing and bathing.

The main motor symptoms are: a) resting tremor - occurs at rest and decreases when voluntary movement is attempted; b) rigidity - simultaneous increase in muscle tone, which may present a constant resistance throughout the range of motion when the movement is slow and progressive or a rhythmically resisted movement throughout the range of motion; c) akinesia - difficulty to initiate movement; d) bradykinesia - slowness to maintain movement.

The tremor in PD is an involuntary rhythmic movement, typically characterized by unilateral resting tremor, which occurs in the upper extremities, especially the hands. Resting tremor is inhibited during movement and may reappear as frequently when adopting a posture or even when moving. The essential tremor occurs more in the forearms and hands, being more problematic during voluntary movement or when maintaining the posture against gravity, which affects the performance of activities of daily living, and the most affected are: writing by hand, eating, dressing, and taking care of oneself. There are also isolated postural and kinetic tremors, with an even higher frequency (greater than 4 Hz).

In turn, rigidity is one of the cardinal symptoms of PD and includes information on severity, distribution, and whether it is present at rest and in a non-drug state. People with PD have higher rigidity values, both at rest and during passive mobilization, because there is an increased tone in the muscles. Reduced range of motion can be a debilitating consequence of PD, affecting daily activities such as writing. Regarding bradykinesia, it refers to the difficulty of adjusting body position, initiating and executing movement, and performing sequential and simultaneous movements.

People with PD generally have difficulty initiating movements, with slower movements and reduced range. This reflects with the difficulty performing most daily activities: personal care, dressing, work and leisure activities and household chores, by the relationship with fine motor coordination and dexterity, as well as bimanual coordination, hypokinesis and initiation.

Currently, there is no approved treatment that changes the progression rate of PD, and the possibilities are focused on the mitigation of symptoms since there is no known cure. The degeneration of dopaminergic neurons, triggers the changes in the basal ganglia network and is mainly treated with medication, such as levodopa or dopaminergic antagonist, being a dopamine replacement therapy, which compensates for the lack of dopamine produced endogenously.

Conventional rehabilitation, associated with other intervention possibilities, in addition physical exercise, are important for the maintenance of motor and non-motor changes, complementing the pharmacological treatment.

Motor imagery (MI) and action observation (AO) are two innovative rehabilitation approaches that are feasible in various pathological conditions. Thus, there is an investment of applying both as a tool in neurorehabilitation, and significant benefits can be induced in PD.

From a systematic review, of the 25 articles selected, there was no record of a study involving the investigation of the joint action of MI and long-term AO, and only one comparing the effect of both in a single session, but the rest dealt with the effect of OA or MI, isolated, in a single session experiment, or the effect of only OA or only MI as a long-term treatment. Another relevant note is that the use of approaches in PD are more directed to motor symptoms in lower limbs, such as balance and gait alterations.

The combination of AO and MI increases imitation in PD, being a promising therapeutic approach for helping people with PD in their activities of daily living and symptom management, since the actions are presented in the context of daily life.Thus, MI and AO used as therapeutic programs can improve motor skills by increasing proprioceptive signals normally generated during movements, or slow down the deterioration of motor skills in PD.

Motor imagery (MI) is a cognitive process that involves the ability to perform an action mentally, without the need to perform the movement itself. The perspective that the person uses to imagine can be: internal perspective (first person - imagines himself/herself), which relates to the person's view of the content of the images or to his/her kinesthetic sensation - the person imagines the movement being performed, as if feeling the movement of the action; or external perspective (third person - imagines another person), which relates to the visual imagination of scenes outside the person.

Like motor execution, MI training can induce improvements in motor performance and therefore in the motor learning processes in PD. Despite the few studies in PD, there is evidence that mental practice can reduce bradykinesia, improve mobility and gait speed, as well as improve dynamic stability, besides showing no tremor in the condition with medication during rest and mental task.

Action observation consists of watching another person acting, or doing a motor task, on video or in real-time. It is recognized that when observing another person acting, there is brain activation in the same neural structures used for the real execution of the same actions, being recruited in the brain of the observer as if performing the observed action.

The AO shows everyday actions, providing information for performing them in life contexts. AO is an effective way to learn or improve the performance of a motor skill, modifying the speed and accuracy of actions in PD. The use of AO in PD improves the spontaneous rate of self-rhythmic finger movements, which reflects in the improvement in the performance of activities of daily living.

The term Brain-Machine Interface (BMI) refers to systems that capture the individual's brain activity signals, translating them into computer commands to control external devices, which can be communication devices, functional electrical stimulation (FES), or robotic exoskeletons. BMI technology is relatively new, and it allows a person to interact with the environment through brain signals and can restore motor function by inducing brain plasticity.

To capture these brain signals, invasive and non-invasive strategies can be used. In non-invasive systems, electrodes are positioned on the skull cap, using signals collected by electroencephalography (EEG), magnetoencephalography (MEG), and functional near-infrared spectroscopy (fNIRS), being more promising than invasive strategies due to safety and ethical issues.

In typical BMI, through EEG, the person's movement intention (motor imagery or execution) is decoded in real-time through the ongoing brain electrical activity triggered by sensory feedback. BMIs are currently used mainly in two applications: in assistive technologies or movement paralysis; and in rehabilitation technologies, also called rehabilitative Brain-Machine Interface or neurofeedback that aims to promote neuroplasticity through manipulation or autoregulation of neurophysiological activity to facilitate motor recovery.

Among numerous neurofunctional dysfunctions, Parkinson's disease (PD) could benefit most from this technology. The use of BMI, through EEG with Functional Electrical Stimulation (FES), to stimulate the muscles of the upper limb during the execution phase of the AO when facing the execution of an observed motor act, may present advantages, such as improved performance, whatever the severity of the neurological impairment, such as severe PD. The literature

presents different BMI employed to people with PD and, so far, there is no study evaluating the training of motor imagery and observation of the action to improve the activity of the sensorimotor cortex with EEG associated with a robotic haptic glove.

Conditions

Conditions: Parkinson Disease

Keywords: Parkinson Disease
Motor Imagery
Action Observation
Upper Limb
Cognition

Study Design

Study Type: Interventional

Primary Purpose: Treatment

Study Phase: N/A

Interventional Study Model: Factorial Assignment

Number of Arms: 5

Masking: Single (Outcomes Assessor)

Allocation: Randomized

Enrollment: 95 [Anticipated]

Arms and Interventions

Arms	Assigned Interventions
Experimental: motor imagery, observation of the action, and execution of the action (GE1)<br>in the sitting position, the participant will watch the recorded video to observe the action, for two minutes. Soon after, they will close their eyes, and receive the description of the action, through recorded audio of the same action, to imagine it, for two minutes. In the sitting or standing position, depending on the action to be performed, the participant must execute the same action imagined previously, for two minutes. Each session will have five different actions that will be repeated twice, totaling 60 minutes of intervention.	Motor Imagery<br>Motor imagery (MI) is a cognitive process that involves the ability to perform an action mentally, without the need to perform the movement itself. The perspective that the person uses to imagine can be: internal perspective (first person - imagines himself/herself), which relates to the person's view of the content of the images or to his/her kinesthetic sensation - the person imagines the movement being performed, as if feeling the movement of the action; or external perspective (third person - imagines another person), which relates to the visual imagination of scenes outside the person.<br>Action Observation<br>Action observation consists of watching another person acting, or doing a motor task, on video or in real-time.<br>Execution of the Action<br>In the sitting or standing position, depending on the action to be performed, the participant must execute the same action imagined or observed previously.
Experimental: motor imagery and execution of the action (GE2)	Motor Imagery<br>Motor imagery (MI) is a cognitive process that involves the ability to perform an action mentally, without the

Arms	Assigned Interventions
in the sitting position, the participant will close his eyes and will receive the description of the action, through recorded audio, to imagine it, for two minutes. Then, in the sitting or standing position, depending on the action to be performed, the participant must execute the same action imagined previously, for two minutes. Each session will have five different actions that will be repeated twice, totaling 40 minutes of intervention.	need to perform the movement itself. The perspective that the person uses to imagine can be: internal perspective (first person - imagines himself/herself), which relates to the person's view of the content of the images or to his/her kinesthetic sensation - the person imagines the movement being performed, as if feeling the movement of the action; or external perspective (third person - imagines another person), which relates to the visual imagination of scenes outside the person.<br>Execution of the Action<br>In the sitting or standing position, depending on the action to be performed, the participant must execute the same action imagined or observed previously.
Experimental: action observation and action execution (GE3)<br>in the sitting position, the participant will watch the recorded video to perform the action observation, which will last two minutes. Then, in the sitting or standing position, depending on the action to be performed, the participant must execute the same action observed previously, for two minutes. Each session will have five different actions that will be repeated twice, totaling 40 minutes of intervention.	Action Observation<br>Action observation consists of watching another person acting, or doing a motor task, on video or in real-time.<br>Execution of the Action<br>In the sitting or standing position, depending on the action to be performed, the participant must execute the same action imagined or observed previously.
Experimental: motor imagery, execution of the action, and exoskeleton (GE4)<br>in the sitting position, the participant will perform the exoskeleton protocol for 40 minutes. Then, the participant will close his eyes and will receive the description of the action, through recorded audio, to imagine it, for two minutes. In the sitting or standing position, depending on the action to be performed, the participant must execute the same action imagined previously, for two minutes. Each session will have five different actions that will be repeated twice, totaling 80 minutes of intervention.	Motor Imagery<br>Motor imagery (MI) is a cognitive process that involves the ability to perform an action mentally, without the need to perform the movement itself. The perspective that the person uses to imagine can be: internal perspective (first person - imagines himself/herself), which relates to the person's view of the content of the images or to his/her kinesthetic sensation - the person imagines the movement being performed, as if feeling the movement of the action; or external perspective (third person - imagines another person), which relates to the visual imagination of scenes outside the person.<br>Exoskeletons<br>The term Brain-Machine Interface (BMI) refers to systems that capture brain activity signals, translating them into computer commands to control external devices, with the robotic exoskeletons. The electrodes are positioned on the skull cap.<br>Other Names:<br>• Brain-Machine Interface<br>Execution of the Action<br>In the sitting or standing position, depending on the action to be performed, the participant must execute the same action imagined or observed previously.
Experimental: observation of the action, execution of the action, and exoskeleton (GE5)<br>in the sitting position, the participant will perform the exoskeleton protocol for 40 minutes.<br>Then, the participant will watch the recorded video to observe the action, for two minutes. Then, in the sitting or standing position, depending on the action	Action Observation<br>Action observation consists of watching another person acting, or doing a motor task, on video or in real-time.<br>Exoskeletons<br>The term Brain-Machine Interface (BMI) refers to systems that capture brain activity signals, translating them into computer commands to control external

Arms	Assigned Interventions
to be performed, the participant must execute the same action observed previously, for two minutes. Each session will have five different actions that will be repeated twice, totaling 80 minutes of intervention.	devices, with the robotic exoskeletons. The electrodes are positioned on the skull cap.<br>Other Names:<br>• Brain-Machine Interface<br>Execution of the Action<br>In the sitting or standing position, depending on the action to be performed, the participant must execute the same action imagined or observed previously.

Outcome Measures

Primary Outcome Measure:

1. Test TEMPA
   The assessment of qualitative are functional scoring and task analysis parameters.

   The functionality is graded according to a 4-level scale: 0, the task was successfully completed without hesitation or difficulty; 1, some difficulty or hesitation in completing the task; 2, the task was partially executed or certain steps were performed with significant difficulty; part of the task may have been modified or the need for assistance by the evaluator may have existed; and 3, could not complete the task, even when assistance was provided. The task analysis quantifies the difficulty found by the subject, according to 5 items relating to UL sensorimotor skills (1) strength; (2) range of motion; (3) accuracy of wide movements; (4) grasp; and (5) accuracy of fine movements.

   For a better interpretation of the evaluation, especially in the task analysis, it will be recorded for later analysis.

   [Time Frame: Pre-intervention (baseline)]
2. Test TEMPA
   The assessment of qualitative are functional scoring and task analysis parameters.

   The functionality is graded according to a 4-level scale: 0, the task was successfully completed without hesitation or difficulty; 1, some difficulty or hesitation in completing the task; 2, the task was partially executed or certain steps were performed with significant difficulty; part of the task may have been modified or the need for assistance by the evaluator may have existed; and 3, could not complete the task, even when assistance was provided. The task analysis quantifies the difficulty found by the subject, according to 5 items relating to UL sensorimotor skills (1) strength; (2) range of motion; (3) accuracy of wide movements; (4) grasp; and (5) accuracy of fine movements.

   For a better interpretation of the evaluation, especially in the task analysis, it will be recorded for later analysis.

   [Time Frame: Post-intervention (10 days of intervention)]

Secondary Outcome Measure:

3. 9-Hole Peg Test
   The 9-Hole Peg Test (9HPT) assesses manual dexterity and allows correlation with severity and PD duration by assessing upper extremity function. The task consists of quickly picking up nine small pins from within a container, one at a time, placing them into holes in a tray, and then moving them back into the container. The execution time is timed by the researcher.

   For men with PD it is, 21.1 seconds for the dominant hand and 22.3 seconds for the non-dominant hand; for women with PD, 19.9 seconds with the dominant hand and 21.4 seconds with the non-dominant hand.

   [Time Frame: Pre Test: one day before the intervention; Pos Test: one day after the intervention.]
4. Unified Parkinson's Disease Rating Scale of the Movement Disorders Society
   The third part (UPDRS-III), involves motor assessment, will be considered, but the following items speech and facial expression, as well as the items corresponding to motor symptoms in lower limbs, will not be considered. Therefore, the following items will be considered: postural tremor and kinetic tremor in the hands, rigidity, continuous finger tapping, hand movements, and alternating rapid hand movements; as well as resting tremor amplitude and resting tremor persistence for the upper limbs.

Each question is anchored with five responses that are linked to commonly accepted clinical terms: 0 = normal, 1 = slight, 2 = mild, 3 = moderate, and 4 = severe. Since there are eight total items, the maximum value in part III of the MDS-UPDRS is 32 points. For the maximum possible score, the higher the score, the worse the symptoms are.

[Time Frame: Pre Test: one day before the intervention; Pos Test: one day after the intervention (last session).]

5. Parkinson's Disease-Cognitive Rating Scale
The Parkinson's Disease Cognitive Rating Scale (PD-CRS) arises from the need for a more comprehensive approach to the cognitive assessment of front-subcortical and cortical functions impaired throughout PD. In PD-CRS there are nine functions assessed, seven involving the "frontal-subcortical": sustained attention, working memory, the immediate and delayed evocation of verbal memory (word list), alternating verbal fluency, verbal fluency of actions, and spontaneous clock drawing; and two involving the "posterior cortical": naming twenty figures by visual confrontation, added to copying the clock drawing. The administration time is approximately 20 minutes, and training is available in the public domain. The total score of the PD-CRS is 134 points, with the ideal cutoff score being 81 points, and a score less than or equal to 64 points indicates PD with dementia.

   [Time Frame: Pre Test: one day before the intervention; Pos Test: one day after the intervention (last session).]

6. Canadian Occupational Performance Measure
The Canadian Occupational Performance Measure (COPM) is a semi-structured interview that allows the identification of important activities, but in which there is difficulty in performing them or cannot perform them, called problem activities. The COPM considers the areas of occupational performance: self-care, productivity, and leisure. The importance score ranges from 1 to 10 points. The most important problem activities (up to five of them) will also be scored regarding performance: 1 point for "unable to do" up to 10 points for "able to do extremely well"; and also, regarding satisfaction: 1 point for "not at all satisfied" and 10 points for "extremely satisfied". In the end, we have an average of the performance value and the satisfaction value.

   [Time Frame: Pre Test: one day before the intervention; Pos Test: one day after the intervention (last session).]

Eligibility

Minimum Age: 20 Years

Maximum Age: 59 Years

Sex: All

Gender Based:

Accepts Healthy Volunteers: No

Criteria: Inclusion Criteria:

- have a diagnosis of Parkinson's disease and be in stage 1-3, consistent with a mild and moderate disability;
- be aged between 20 years old to 59 years old;
- be on stable use of medication;
- no cognitive impairment or dementia;
- be minimally capable of imagining motor activities;
- present motor alteration in the dominant upper limb;
- have signed the Informed Consent Form.

Exclusion Criteria:

- not present additional central nervous system disorders or other conditions that may affect upper and lower extremity function;
- present with other uncontrolled chronic conditions that may interfere with the participant's safety.

Contacts/Locations

Central Contact Person:

Central Contact Backup:

Study Officials:

Locations:

IPDSharing

Plan to Share IPD:

References

Citations:

Links:

Available IPD/Information:
