# Supplementary material for: The use of brain-machine interface, motor imagery, and action observation in the rehabilitation of individuals with Parkinson’s disease: A protocol study for a randomized clinical trial
Source: PLoS One. 2025 Apr 7;20(4):e0315148. doi: 10.1371/journal.pone.0315148 (PMC11975075; doi:10.1371/journal.pone.0315148)
Supplement: S4 File — (PDF) [file pone.0315148.s009.pdf]

FEDERAL UNIVERSITY OF HEALTH SCIENCES OF PORTO ALEGRE
POSTGRADUATE DIPLOMA IN REHABILITATION SCIENCES

Kátine Marchezan Estivalet

EFFECTS OF MOTOR IMAGERY AND ACTION OBSERVATION ON MOTOR CHANGES IN THE UPPER LIMBS AND COGNITIVE CHANGES IN PARKINSON'S DISEASE: A RANDOMIZED CLINICAL TRIAL

Research Team: Physiotherapists Rafael Goldani and Tatiana Salayaran de Aguiar Pettenuzzo

Porto Alegre

2022

Kátine Marchezan Estivalet

EFFECTS OF MOTOR IMAGERY AND ACTION OBSERVATION ON MOTOR CHANGES IN THE UPPER LIMBS AND COGNITIVE CHANGES IN PARKINSON'S DISEASE: A RANDOMIZED CLINICAL TRIAL

Doctoral Research Project submitted to the Graduate Program in Rehabilitation Sciences of the Federal University of Health Sciences of Porto Alegre, for thesis qualification.

Advisor: Profa. Dra. Fernanda Cechetti

Porto Alegre

2022

SUMMARY

Parkinson's disease is degenerative, progressive, and chronic. It is considered potentially disabling, in view of motor alterations, such as bradykinesia, rigidity and tremor in the upper limbs, and non-motor alterations, such as cognitive alterations involving difficulties in attention and concentration and memory. Thus, there has been a focus on neurorehabilitation modalities, such as motor imagination and observation of action. The objective of the research is to investigate the effects of motor imagery and action observation on motor changes in the upper limbs and cognitive changes in Parkinson's disease. This is a randomized controlled clinical trial type study. The study population involves people with Parkinson's disease at stage 1-3 on the Hoehn and Yahr scale, aged between 20 and 59 years, and must be on stable medication use, have no cognitive impairment at risk of dementia, and be able to imagine motor activities, and have motor impairment in the upper limb. The study groups will be: a) motor imagination, observation of action and motor execution; b) motor imagination and motor execution; c) observation of the action and motor execution; d) motor imagery and motor execution and exoskeleton; e) observation of motor action and execution and exoskeleton. The interventions of all groups will be of an intensive approach of 10 continuous sessions, with an interval of two days in the middle of the intervention, totaling two weeks, with each session of 60 minutes per day. The steps for data collection of the study will involve pre-testing, interventions, immediate post-testing, and testing after a four-week intervention-free period. The instruments that will be used for the evaluations: a) part of the Unified Parkinson's Disease Assessment Scale (UPDRS-III); (b) *Test D'évaluation des membres supérieurs des personnes âgées* (TEMPA); c) *9-Hole Peg Test* to evaluate upper extremity function; d) Cognitive Assessment Scale for Parkinson's Disease; e) Canadian Measure of Occupational Performance to identify performance and satisfaction in the performance of problem activities that it considers important in the areas of self-care, productivity and leisure.

Keywords: Cognition. Parkinson's disease. Imagination. Rehabilitation.

LIST OF ABBREVIATIONS AND ACRONYMS

9HPT 9-Hole Peg Test
BCI *Brain Computer Interface*
CEP Research Ethics Committee
COPM Canadian Occupational Performance Measure
PD Parkinson's Disease
EA Execution of the Action
EEG Electroencephalography
ICM Brain-Machine Interface
IM Motor Imagination
fNIRS Functional Infrared Spectroscopy
GE Experimental Groups
GR Reference Group
KVIQ-10 Kinesthetic and Visual Imagery Questionnaire
MDS-UPDRS Movement Disorders Society Unified Parkinson's Disease Rating Scale
MEG Magnetoencephalography
MoCA *Montreal Cognitive Assessment*
OA Action Observation
PD-CRS Parkinson's Disease Cognitive Assessment Scale
RS Rio Grande do Sul
TEMPA *Test d'Évaluation des Membres Supérieurs of Personnes Âgées*
Informed Consent Form
UFCSPA Federal University of Health Sciences of Porto Alegre
UFSM Federal University of Santa Maria

SUMMARY

1 INTRODUCTION AND RATIONALE FOR THE STUDY....................................6
2 THEORETICAL FRAMEWORK...7
3 RESEARCH PROBLEM...20
4 HYPOTHESES..20
4.1 Null Hypothesis...20
4.2 Alternative hypothesis...20
5 OBJECTIVES...20
5.1 General Objective..20
5.2 Specific Objectives..20
6 STUDY METHODOLOGY...21
6.1 Design...21
6.2 Area of Coverage..21
6.3 Location of the research..21
6.4 Population and sample..22
6.5 Eligibility Criteria..22
6.5.1 Inclusion Criteria..22
6.5.2 Exclusion criteria...22
6.6 Assessment tools...23
6.6.1 Hoehn and Yahr scale...23
6.6.3 Kinesthetic and Visual Imagery Questionnaire......................................23
6.6.4 *9-Hole Peg Test*...24
6.6.5 Movement Disorders Society Unified Parkinson's Disease Rating Scale...............25
6.6.6 *Test d'évaluation des membres supérieurs des personnes âgées*...........................25
6.6.7 Parkinson's Disease-Cognitive Rating Scale...26
6.6.8 Canadian Occupational Performance Measure.......................................27
6.7 Outcomes..27
6.7.1 Main Outcome..27
6.7.2 Secondary Outcome..28
6.8 Data Collection Procedure..28
6.8.1 Stages of the Study...28
6.8.2 Intervention Proposal...28
6.9 Data Analysis..30
6.10 Sample Calculation...31
6.11 Ethical and legal procedures..31
6.12 Risks and Benefits...31
7 SCHEDULE OF ACTIVITIES...33
8 BUDGET 34
REFERENCES...35
APPENDIX 1 – INITIAL ASSESSMENT...44
APPENDIX 2 - INFORMED CONSENT FORM.....................................45
ANNEX 1 – STAGES OF PARKINSON'S DISEASE ACCORDING TO HOEHN AND YAHR……………………………………………………………...49
ANNEX 2 – MONTREAL COGNITIVE ASSESSMENT…………………………..50
ANNEX 3 – KINESTHETIC AND VISUAL IMAGINATION QUESTIONNAIRE..51
APPENDIX 4 - AVERAGE TIME TO PERFORM THE TASK ACCORDING TO THE STAGES OF PARKINSON'S DISEASE…………………………......52

APPENDIX 5 - UNIFIED PARKINSON'S DISEASE ASSESSMENT OF THE MOVEMENT DISORDERS SOCIETY……………………………………53
ANNEX 6 - TEST D'ÉVALUATION DES MEMBRES SUPÉRIERS DE PERSONNES AGÉES…………………………...………………………59
ANNEX 7 – PARKINSON'S DISEASE-COGNITIVE RATING SCALE……..….60
ANNEX 8 - CANADIAN OCCUPATIONAL THERAPY MEASURE...................73

1 INTRODUCTION AND STUDY JUSTIFICATION

Parkinson's disease (PD) is a progressive and degenerative condition that leads to disability due to its motor and non-motor complications (NIELSEN et al., 2020). Its symptoms negatively impact motor control and motor planning, interfering with the use of the upper limbs in performing daily activities (BEK et al., 2016). Furthermore, non-motor symptoms, particularly cognitive impairments, also affect the execution of daily tasks that involve multiple stimuli, further incapacitating individuals with PD (KOBAYASHI et al., 2022).

Although pharmacological treatments exist, the regulation of dosages and duration of use remains challenging, particularly in combination with disease progression (ABRAMI et al., 2020). Other treatment alternatives are available, some of which are invasive, such as deep brain stimulation surgery. Rehabilitation, on the other hand, seeks to complement treatments through various resources, including alternative approaches combined with physical exercises (ABBRUZZESE et al., 2015).

Given the interaction between cognitive and motor components in PD, it is essential to consider these alterations throughout the assessment and rehabilitation process. Despite being a recommended and promising approach in PD rehabilitation, motor imagery remains under-researched, resulting in weak evidence and limited protocols. Similarly, action observation is still scarcely explored in rehabilitation, particularly concerning the upper limbs in PD. Existing approaches primarily target gait disturbances and postural stability. Despite PD causing significant impairments in hand movements, few studies have focused on therapeutic approaches for the upper limbs (BEK et al., 2021).

Therefore, the development of this research project is justified by its originality in investigating the effects of motor imagery and action observation on motor and cognitive impairments in the upper limbs in PD, with potential applications in rehabilitation. This study may contribute to the development of intervention protocols. Notably, there is a lack of studies examining the isolated practice of motor imagery, as it is typically applied in combination with other types of interventions, including in PD. Additionally, little research has explored the combined effects of action observation and motor imagery on the upper limbs, particularly hand function. In previous studies, training has been conducted separately (BEK et al., 2021).

Moreover, technology has been increasingly incorporated into rehabilitation in an accessible manner, such as in brain-computer interface applications. Regarding the use of upper limb exoskeletons, current applications are primarily focused on other neurological disorders. This study offers a novel approach by investigating its effects on PD symptoms. The exoskeleton presents a promising avenue for PD rehabilitation by facilitating neuronal activation and, consequently, promoting movement recovery.

Thus, this research project aims to explore the effects of motor imagery and action observation on upper limb motor and cognitive impairments in PD, as well as the potential use of exoskeletons as rehabilitation tools.

2 THEORETICAL FRAMEWORK

Parkinson's Disease

Among chronic and potentially disabling conditions, Parkinson's disease (PD) is one of the leading neurological causes of impairment. As is well known, PD is a degenerative disorder that progresses slowly over time, is idiopathic, and involves multiple factors (CIKAJLO; PETERLIN, 2019; NIELSEN et al., 2020). It affects approximately 7 to 10 million people worldwide, typically beginning in adulthood between the ages of 35 and 60 (CIKAJLO; PETERLIN, 2019). It is the second most common neurodegenerative disease affecting the elderly (PONDÉ et al., 2019), with a prevalence of 0.5% to 1% among individuals aged 65 to 69 years and 1% to 3% among those over 80 years old (FLORIANO et al., 2015).

Under normal conditions, the midbrain processes visual and auditory information and generates voluntary somatic responses. In the substantia nigra, which contains pigmented cells, excitatory neurons are kept inactive due to the effects of dopamine (PAUL et al., 2020). Dopamine is a neurotransmitter that facilitates and modulates neuronal activity (ABRAMI et al., 2020) and plays a key role in regulating motor output from the basal ganglia—structures responsible for motor coordination, control, feedback, and muscle contractions. In PD, there is degeneration of dopamine-producing neurons (dopaminergic neurons) in the substantia nigra within the basal ganglia (PAUL et al., 2020). This leads to the activation of excitatory neurons, resulting in dysregulated motor control (ABRAMI et al., 2020).

PD is characterized by both motor and non-motor symptoms (ABBRUZZESE et al., 2015), which negatively impact quality of life and interfere with various daily activities. Non-motor symptoms are considered additional yet frequent manifestations (FERREIRA et al., 2018). These include reduced facial expression, dysphagia, and dysarthria, as well as emotional disturbances such as anxiety and depression (SOUSA; MACEDO; BRUCKI, 2021). Non-motor symptoms also encompass cognitive impairments, including mild cognitive impairment and dementia (ROSCA; SIMU, 2020). The most commonly observed cognitive deficits in PD involve concentration and attention, working memory (NIELSEN et al., 2020), recent memory, difficulties with calculations, spatial orientation tasks, and executive functions (SOUSA; MACEDO; BRUCKI, 2021). These non-motor symptoms negatively affect occupational performance, leading to difficulties in activities such as driving, shopping, household maintenance, as well as self-care tasks like dressing and bathing (KOBAYASHI et al., 2022).

The primary motor symptoms of PD include (ABRAMI et al., 2020): a) **Resting tremor** – occurs at rest and decreases with voluntary movement; b) **Rigidity** – simultaneous increase in muscle tone, which may present as constant resistance throughout the range of motion during slow and progressive movements or as rhythmic resistance during movement; c) **Akinesia** – difficulty initiating movement; d) **Bradykinesia** – slowness in maintaining movement.

PD manifests with different types of tremor depending on the circumstances, affected body part, and tremor frequency (HELMICH et al., 2012). The tremor in PD is an involuntary, rhythmic movement, typically characterized by unilateral resting tremor, primarily affecting the upper extremities, especially the hands (MALLING et al., 2019). Tremor is considered a key marker of PD, as tremor-dominant PD is associated with relatively slower disease progression and less severe dopaminergic dysfunction (HELMICH et al., 2012).

Even during slow movements, tremor (approximately 7 Hz) is observed in the upper limbs (VAN DEN NOORT et al., 2017). However, it can also affect the head, lower limbs, and even the trunk, leading to performance difficulties (HU et al., 2019). Individuals with tremor-dominant PD exhibit significantly more resting and postural tremors, whereas those without tremor experience more axial and gait-related symptoms, as well as speech-related impairments and hypomimia (HELMICH; BLOEM; TONI, 2012).

Resting tremor is inhibited during movement but may reappear with the same frequency when adopting a posture or performing an action (HELDMAN et al., 2011). A higher-frequency tremor (>1.5 Hz), though less common, is classified as essential tremor, predominantly affecting the forearms and hands. Essential tremor is most problematic during voluntary movement or while maintaining a posture against gravity, significantly impacting daily activities such as handwriting, eating, dressing, and self-care (HELDMAN et al., 2011). Postural and kinetic tremors, which occur at even higher frequencies (>4 Hz), have also been identified (HELMICH et al., 2012).

Tremor-related movement must be distinguished from voluntary movement before tremor quantification can be performed. Voluntary movements typically occur at lower frequencies (<3 Hz), while tremors are observed at higher frequencies (>4 Hz) (HELDMAN et al., 2011). However, it remains unclear how tremor interferes with voluntary movement performance in dynamic tasks, such as rapid reaching movements, which limits the development of non-invasive rehabilitation strategies for individuals with tremor-dominant PD (HU et al., 2019). For example, in daily tasks such as folding clothes, which involve free movement without rhythmic patterns, tremor can still be distinguished from voluntary movement due to its distinct spectral peak, while voluntary movements contain multiple frequency components (HELDMAN et al., 2011).

Rigidity is one of the cardinal symptoms of PD and is characterized by severity, distribution, and its presence at rest or in an unmedicated state (ZETTERBERG et al., 2015). Rigidity depends on angular velocity and joint amplitude during mobilization (FERREIRA-SÁNCHEZ; MORENO-VERDÚ; CANO-DE-LA-CUERDA, 2020). Increased joint excursion and muscle stretching lead to a dominance of stretch reflexes over shortening reactions, resulting in greater rigidity (POWELL et al., 2011). Additionally, individuals with PD have difficulty relaxing unused muscles during a task, further increasing rigidity during contralateral activation maneuvers (ZETTERBERG et al., 2015).

PD patients exhibit higher rigidity values both at rest and during passive mobilization due to increased muscle tone, as evidenced by electromyographic activity, biomechanical deformation responses, and stretch resistance, which can be detected using instrumented assessment tools (FERREIRA-SÁNCHEZ; MORENO-VERDÚ; CANO-DE-LA-CUERDA, 2020). A biomechanical model has identified increased neural contributions to passive movement resistance in the hand and finger flexor muscles of PD patients (ZETTERBERG et al., 2015).

The effects of movement amplitude on rigidity in PD are less understood and rarely studied. However, evidence suggests that supraspinal mechanisms likely contribute to the amplitude-dependent nature of PD-related rigidity (POWELL et al., 2011). Both movement amplitude and velocity modulate PD rigidity and serve as parameters for assessing rigidity through manually applied passive joint movements (POWELL et al., 2012).

Treatments for Parkinson's disease

Currently, there is no approved treatment that changes the rate of PD progression (ABRAMI et al., 2020), and the possibilities are focused on alleviating symptoms, since there is no known cure. Therefore, the management of PD is traditionally based on symptomatic treatment (ABBRUZZESE et al., 2015).

The degeneration of dopaminergic neurons, which triggers changes in the basal ganglia network, is mainly treated with medication, such as levodopa or dopaminergic antagonist (CIKAJLO, PETERLIN, 2019), being a dopamine replacement therapy, which compensates for the lack of dopamine produced endogenously (ABRAMI et al., 2020). Prescribing an adequate amount of medication is important, as insufficient dosage leads to insufficient symptom control; Excessive medication leads to faster habituation and potentially disabling dyskinesias (ABRAMI et al., 2020). On the other hand, with the progression of the disease, the action of levadapa decreases, resulting in a lower drug response over time (CIKAJLO, PETERLIN, 2019). Remembering, therefore, that the lack of dopamine results in slower movements, and in PD with tremor there is a reduced capacity for motor control (HU et al., 2019).

As PD is progressive, there is a need for changes in treatment and continuous long-term monitoring, involving a personalized plan to maintain symptom control, as well as avoiding medication side effects (SHAWEN et al., 2020). With the use of medication, an improvement can be seen in all parameters regarding the opening and closing movements of the hand, such as an increase in speed and range of movement (VAN DEN NOORT et al., 2017). In the condition without medication, there is an irregularity in movement, such as in pronation and supination movements, making it difficult to perform due to the small range of movement (VAN DEN NOORT et al., 2017). Object manipulation depends on fine hand and finger coordination and cannot be restored by dopamine levels, since finger dexterity is insensitive to dopaminergic treatment

(CIKAJLO, PETERLIN, 2019). Therefore, exogenous dopamine - replacement medication - can have positive effects on the motor skills of the upper extremities, existing based on the type of task and practice resources, in mild to moderate PD. However, there are potentially harmful effects on the (re)learning of motor skills in the context of rehabilitation (PAUL et al., 2020).

Motor symptoms have direct responses to dopaminergic medication and deep brain stimulation and are therefore often used to judge the effects of such therapies (VAN DEN NOORT et al., 2017). So much so that the assessment of finger touch, including speed, amplitude and rhythm, contributes to the discrimination of PD (ENDO et al., 2011).

Conventional rehabilitation, associated with other intervention possibilities, as well as the practice of physical exercises, are important for maintaining motor and non-motor changes, complementing pharmacological treatment (ABBRUZZESE et al., 2015). There is also the use of unconventional strategies, such as music therapy and dance and martial arts (ABBRUZZESE et al., 2015). However, even though rehabilitation induces short-term, clinically important benefits, mainly for gait and balance, interventions are largely heterogeneous, and there is still no consensus on the ideal approach (ABBRUZZESE et al., 2015). But it is known that, even considering motor recovery, most approaches do not involve the organization of the motor system (BUCCINO, 2014).

Thus, rehabilitation must be aimed at practicing and learning specific activities in the central areas that are impaired, such as manual activities, leading to better performance in activities of daily living (ABBRUZZESE et al., 2015). Therefore, therapies for PD should be aimed at improving or maintaining movement control, such as facilitating initiation and increasing amplitude or speed (BEK et al., 2016). As neuroplasticity is largely dependent on the intensity, repetition, specificity, difficulty and complexity of the practice, it is important to remember that the time to achieve effective learning and automation is longer in PD (ABBRUZZESE et al., 2015).

Motor Imagination and Action Observation

Motor imagination (MI) and action observation (OA) are two innovative rehabilitation approaches that are viable in various pathological conditions, even though studies have existed for a long time with healthy people (CALIGIORE et al., 2017). Thus,

there is an investment in applying both as a tool in neurorehabilitation (BUCCINO, 2014), potentially capable of inducing significant benefits in PD (ABBRUZZESE et al., 2015).

However, the majority of studies carried out are using the approaches alone or associated with other rehabilitation practices. In the case of MI, there are studies focused on the treatment of motor symptoms, especially with stroke in the acute and chronic phases (CALIGIORE et al., 2017). In a systematic review of the literature, to investigate mental practice protocols in motor rehabilitation in PD, only four studies were identified (BRAUN et al., 2011; TAMIR; DICKSTEIN; HUBERMAN, 2007; EL-WISHY, FAYEZ, 2013 ; SANTIAGO et al., 2015) with the associated use of motor imagination with conventional rehabilitation, with the majority using visual or visual motor imagination. and kinesthetic, with the main objective of improving mobility and gait (SILVA et al., 2016). In the case of OA, there is the involvement of studies with other neurological pathologies such as stroke (BUCCINO, 2014) and cerebral palsy, as well as post-surgical orthopedic pathologies (CALIGIORE et al., 2017; SARASSO et al., 2015).

From a systematic review, of the 25 selected articles, there was no record of any study involving the investigation of the joint action of MI and long-term OA, and only one comparing the effect of both in a single session, but the rest were it dealt with the effect of OA or MI, alone, in a single-session experiment, or the effect of just OA or just MI as a long-term treatment (CALIGIORE et al., 2017). Another relevant note is that the use of approaches in PD are more focused on motor symptoms in the lower limbs, such as changes in balance and gait (PELOSIN et al., 2010; KIKUCHI et al., 2014).

OA is triggered by visual stimuli of external origin and in IM by internal stimuli - reactivation of a motor representation stored in memory (CALIGIORE et al., 2017). MI training is as effective as motor execution training in the first phase of learning, that is, it can induce improvements in motor performance and, therefore, in motor learning processes (ABBRUZZESE et al., 2015). However, for the consolidation and retention of motor skills to occur effectively, it is suggested to provide an external sensory stimulus (BONASSI et al., 2020), such as OA. Thus, it can be mentioned that there is a sharing between OA and MI as an internal reproduction of behavior, which improves learning and the neural traits of motor actions (CALIGIORE et al., 2017).

It is therefore pointed out that an increase in the behavioral and neural effects of OA may occur with MI, by activating the motor system, influencing movement and increasing learning, demonstrating that MI improves the speed and time of movement in people with DP (BEK et al., 2018) and OA the speed and precision of actions

(ABBRUZZESE et al., 2015). Cortical activity related to the execution of observed and imagined movements is induced by motor simulations through OA and MI (KANEKO et al., 2021), recruiting high-level brain processes involved in motor behavior (CALIGIORE et al., 2017).

There is a finding that OA is better than MI as a strategy for learning a new complex motor task, at least at the beginning of the rapid phase of motor learning (GATTI et al., 2013; BUCCINO, 2014). However, the combination of OA and MI increases imitation in PD, being a promising therapeutic approach (BEK et al., 2018), as it helps people with PD in their daily activities and symptom management (BEK et al ., 2016), since the actions are presented in the context of everyday life (GATTI et al., 2013). Thus, MI and OA used as therapeutic programs can improve motor skills by increasing proprioceptive signals normally generated during movements (ABBRUZZESE et al., 2015), or delay the deterioration of motor skills in PD (CALIGIORE et al., 2017).

Motor Imagination

Motor imagination (MI) is a cognitive process that involves the ability to perform an action mentally, without the need to perform the movement itself (ABBRUZZESE et al., 2015; BEK et al., 2016). It is defined as the possibility of imagining a motor action without physically executing it (SILVA et al., 2016), involving the process of creating visual, auditory or kinesthetic experiences in the mind (ABRAHAM et al., 2018). The ability to mentally simulate a certain movement is strictly linked to the correspondence of the movement with the personal motor repertoire (AVANZINO et al., 2013).

Even when still inconclusive, especially regarding the lack of knowledge about the neural basis of the rehabilitative effect in PD, MI was already considered promising (FISCHER et al., 2017). MI uses the same internal motor representations as movements performed at a specific neural level (PONDÉ et al., 2019): ventral and dorsal part of the premotor cortex, supplementary motor area, anterior cingulate cortex, superior parietal lobule and parietal lobule inferior, basal ganglia and cerebellum. Thus, with a better understanding of the network activity underlying motor images, it was possible to inform the best way to take advantage of the therapeutic potential of MI as an adjuvant to rehabilitation in people with Parkinson's disease (FISCHER et al., 2017).

In MI, there are explicit and implicit experiments with divergent relationships with motor cognition (DI RIENZO et al., 2014). The perspective that the person uses to

imagine can be: internal perspective (first person - imagines themselves), which relates to the person's vision of the content of the images or to their kinesthetic sensation - the person imagines the movement being performed , as if feeling the movement of the action; or external perspective (third person - imagine another person), which is related to the visual imagination of scenes outside the person (ABBRUZZESE et al., 2015; SILVA et al., 2016).

Regarding the internal perspective, it is interesting to mention that visual images are considered easier than kinesthetic images, as they involve the practice of mental images (HEREMANS et al., 2012). In fact, 85% of studies identified the use of a systematic review of first-person visual MI, sometimes combined with kinesthetic information (DI RIENZO et al., 2014). Therefore, it is suggested to first learn visual MI as it involves tasks that emphasize form, and then incorporate kinesthetic MI for tasks that emphasize timing or hand coordination (FÉRY, 2003; SILVA et al., 2016). From MI performed visually or kinesthetically, there is preparation for the next movement sequences (HEREMANS et al., 2012), effectively facilitating the learning of skills (BONASSI et al., 2020).

Like motor execution, MI training can induce improvements in motor performance and, therefore, in motor learning processes in PD (ABBRUZZESE et al., 2015). Therefore, MI is being seen as a promising new rehabilitation method for people with neurological disorders, mainly cerebrovascular diseases, having relevance and potential for PD (ABRAHAM et al., 2018), adjusting to motor limitations, such as decreased the amplitude or speed of movement (HEREMANS et al., 2012). Despite the few studies on PD, there is evidence that mental practice can reduce bradykinesia, improve mobility and gait speed (SILVA et al., 2016), as well as improve dynamic stability (SANTIAGO et al., 2015), in addition to not showing tremor in the condition with medication during rest and during the mental task (VAN DEN NOORT et al., 2017).

The ability of people with PD to imagine movements efficiently is still controversial. Although the use of levodopa has been suggested to normalize brain activity in several cortical areas (including the supplementary motor area), people with PD are able to imagine in a similar way to older adults whether on or off the medication (ABBRUZZESE et al. ., 2015). Remember that the performance of imagery tasks is slower in PD, but the sharpness and precision of motor imagery are preserved (HEREMANS et al., 2012). It is also worth highlighting that an important advantage of

MI is using it to practice potentially dangerous movements in a safe way (HEREMANS et al., 2011; HEREMANS et al., 2012).

Observation of Action

Action observation consists of observing another person performing an action, or motor task, on video or in real time (SARASSO et al., 2015). There is recognition that, when observing the performance of an action by another person, there is activation in the brain in the same neural structures used for the actual execution of the same actions, being recruited in the brain of the person observing as if they were actually carrying out the observed action (BUCCINO, 2014; ABBRUZZESE et al., 2015). Thus, OA awakens a mirror mechanism that serves the brain's ability to couple an observed action with its motor counterpart in the observer's brain (GATTI et al., 2013).

In OA, specific areas are recruited in the frontal and parietal lobes in a similar way to what happens during motor execution: ventral premotor cortex, posterior part of the inferior frontal gyrus, rostral part of the inferior parietal lobule and posterior superior temporal sulcus (ABBRUZZESE et al., 2015). Thus, what was previously a concern that OA did not cause a repair of the underlying neural circuits, there is now another panorama that used as a neurorehabilitation approach has the possibility of restoring neural structures or activating supplementary or related pathways to perform the original functions (BUCCINO, 2014).

In OA, daily actions can be shown, providing information for carrying them out in life contexts (BUCCINO, 2014). OA is an effective way to learn or improve the performance of a motor skill, modifying the speed and precision of actions in PD (ABBRUZZESE et al., 2015). It is known that people with PD have the ability to adjust the amplitude of hand movements in response to observed actions, indicating the potential for OA-based interventions to increase the amplitude as well as the speed of movements (BEK et al., 2018). The observation of rapid, rhythmic finger movements implicitly induces an increase in the rate of spontaneous finger movement in people with PD, thus improving bradykinesia (PELOSIN et al., 2013). The use of OA in PD improves the rate of spontaneous movement of self-paced finger movements, which reflects in improvements in carrying out activities of daily living (SARASSO et al., 2015).

The Brain Machine Interface

The term Brain–Machine Interface (ICM), which in English is called Brain Computer Interface (BCI), refers to systems that capture signals from an individual's brain activities, translating them into computerized commands to control external devices, which can be communication devices (SELLERS; DONCHIN, 2006), functional electrical stimulation (FES) (LI et al., 2014) or robotic exoskeletons (ANG et al., 2015), among others. ICM technology is relatively new, and allows the person to interact with the environment through brain signals and can restore motor function by inducing brain plasticity (ANG et al., 2011).

To capture these brain signals, invasive and non-invasive strategies can be used. In invasive ICM, spatio-temporal signals can be acquired and have a greater capacity to distinguish the dimensions of the individual's intention through electrodes positioned on the surface of the brain (electrocorticography or ECoG) or implanted within the cerebral cortex (microelectrodes) (MILLER et al., 2010; NISHIO; PFURTSCHELLER, 2006; et al., 2008). In non-invasive systems, electrodes are positioned on the skullcap, using signals collected by electroencephalography (EEG), magnetoencephalography (MEG), functional infrared spectroscopy (fNIRS). However, non-invasive brain-machine interfaces may be more promising than invasive strategies due to safety and ethical concerns (BIRBAUMER; COHEN, 2007).

Among those mentioned above, ICM based on brain signals captured through EEG is the most commonly used system as it is a simpler and cheaper piece of equipment (WOLPAW et al., 2002). In typical ICM, through EEG, the intention of the individual's movement (motor imagery or execution) is decoded in real-time through ongoing electrical brain activity, extracting the relevant characteristics from this. And the detection of movement intention would trigger contingent sensory feedback to the user (ALIMARDANI; NISHIO; ISHIGURO, 2016; PFURTSCHELLER; NEUPER, 2006; BUCH et al., 2008)

ICM are currently used mainly in two applications, the first in assistive technologies, which aim to restore lost functions, for example, for communication in locked-in syndrome (PFURTSCHELLER; LOPES DA SILVA, 1999) or in movement paralysis, in tetraplegia, using robotic actuators and/or a functional electrical stimulation system for the daily task of feeding (ZHANG et al., 2018). The second, in rehabilitation technologies, also called rehabilitative Brain-Machine Interface or neurofeedback

(BANIQUED et al., 2021) which aims to promote neuroplasticity through manipulation or self-regulation of neurophysiological activity to facilitate motor recovery.

In the last five years, several researches have been developed in the area of neurological rehabilitation, with the majority of studies focusing on the Brain-Machine Interface and cerebrovascular accident (CVA), with an interest in promoting the functional recovery of the upper limb, as it is frequently and widely affected and generate a major limitation in activities of daily living (LAWRENCE et al., 2001; MONGE-PEREIRA et al., 2017). A 2018 review by Mehrhols and colleagues selected randomized controlled trials that used robot-assisted electromechanical upper limb training to improve activities of daily living, function, and arm muscle strength in post-stroke patients (MEHRHOLZ et al., 2018).

The results included 45 studies involving 1,619 participants, comparing robot-assisted electromechanical arm training for recovery of arm function with other rehabilitation interventions or placebo, or no treatment. Training with the robot-assisted electromechanical arm promoted significant improvement in activities of daily living, function and arm muscle strength. However, the results must be interpreted with caution, although the quality of the evidence was high, as variations between trials in aspects of intensity, duration and quantity of training must be observed; type of treatment; characteristics of the participants.

The assistance of robotic technologies in the functional recovery process is providing advances in research on ICM, especially those related to stroke. However, it is noted that in relation to other neurological pathologies there are few studies related to this rehabilitation technology. Among numerous neurofunctional dysfunctions, Parkinson's disease (PD) could benefit most from this technology, as movement disorders of the upper limbs may be present from diagnosis, often presenting micrography or resting tremor as the initial symptom of the disease (DICKSON; GRU¨NEWALD, 2004), difficulty with speed and dexterity in manipulating objects (GEBHARDT et al., 2008; PROUD; MORRIS, 2010; MANSON; CAIRD, 1985). As the disease progresses, these upper limb disorders can lead to greater limitations in work, recreation and daily tasks such as dressing and eating (STURKENBOOM et al., 2011), impacting the quality of life and functionality of these individuals.

Yongbin and colleagues in 2015 investigated the resting-state functional connectivity patterns of the whole brain of patients with Parkinson's disease (PD) through pattern recognition and neuroimaging techniques that resulted in providing additional

information for clinical diagnosis. and evaluation of the treatment of the disease. Another interesting study was that of Hanson and collaborators in 2012, with the application of invasive ICM, in which 25 patients who had essential tremor or Parkinson's disease underwent neurosurgery to place therapeutic Deep Brain Stimulation implants in a set of neurons in the subcortical region, with their activities recorded during the performance of a target tracking motor task using a cursor controlled by a haptic glove. Modulations in the firing rate of a substantial number of neurons were observed to account for target onset, movement onset/direction, and hand tremor. Notably, all tremor-associated neurons exhibited synchrony within the ensemble, with the subcortical neuron ensemble being largely unexplored but with the potential to advance both neuroscience and neurorehabilitation.

The use of ICM, through EEG with Functional Electrical Stimulation, with the aim of stimulating the muscles of the upper limb during the execution phase of OA in the face of the execution of an observed motor act, can present advantages, such as improved performance, whatever the severity of their neurological impairment, such as severe Parkinson's disease (ROSSI et al., 2021).

The literature presents different ICM used for people with PD and, to date, there is no study evaluating motor imagination training and action observation to improve the activity of the sensorimotor cortex (SM1) with EEG associated with a robotic haptic glove. As an ICM is non-invasive, capable of providing rapid and effective neurofeedback on the activation of the region of interest, it is promising for improving the daily and functional activities of the compromised upper limb.

3 RESEARCH PROBLEM

Do motor imagination and action observation, when applied alone or in combination, positively interfere with motor changes in the upper limbs and cognitive changes in Parkinson's disease?

4 HYPOTHESES

4.1 Null Hypothesis

H0 - There will be no improvement in upper limb motor symptoms after treatment.

H0 – There will be no improvement in cognitive symptoms after treatment.

H0 – There will be no improvement in performance and satisfaction in carrying out occupations after treatment.

4.2 Alternative Hypothesis

H1 - There will be improvement in upper limb motor symptoms after treatment.

H1 – There will be an improvement in cognitive symptoms after treatment.

H1 – There will be an improvement in performance and satisfaction in carrying out occupations after treatment.5

5 OBJECTIVES

5.1 General Objective

Investigate the effects of motor imagery and action observation on upper limb motor impairments and cognitive alterations in Parkinson's disease.

5.2 Specific Objectives

- Analyze the isolated effects of motor imagery and action observation on upper limb motor impairments using the *Test d'Évaluation des Membres Supérieurs of Personnes Âgées* (TEMPA) and the 9-Hole Peg Test (9HPT), as well as on cognitive alterations using the *Parkinson's Disease-Cognitive Rating Scale* (PD-CRS).
- Analyze the effects of combining motor imagery and action observation on upper limb motor impairments using the *Test d'Évaluation des Membres Supérieurs of Personnes Âgées* (TEMPA) and the 9-Hole Peg Test (9HPT), as well as on cognitive alterations using the *Parkinson's Disease-Cognitive Rating Scale* (PD-CRS).
- Compare the effects of motor imagery combined with action execution versus action observation combined with action execution.
- Compare the effects of using an exoskeleton combined with motor imagery and action execution versus action observation and action execution.
- Assess the impact of motor imagery and action observation on occupational performance in activities related to self-care, productivity, and leisure using the *Canadian Occupational Performance Measure* (COPM).

6 STUDY METHODOLOGY

6.1 Design

This is a randomized controlled clinical trial with a single-blind design, in which participants will be randomly assigned to groups for the application of interventions over a specific period, followed by an analysis of the study outcomes (KANG; RANG; PARK, 2008). This study design is justified by its potential to provide evidence for clinical practice.

6.2 Coverage Area

Major area: Health Sciences.
Area: Physiotherapy and Occupational Therapy.

6.3 Search location

The research will be carried out in two different locations. In the city of Santa Maria, the research will take place on the premises of the Federal University of Santa Maria, in the building linked to the Department of Occupational Therapy. In the city of Porto Alegre, the research will take place at NeuroGold, a reference clinic in the functional rehabilitation of individuals with neurological pathologies.

6.4 Population and sample

The target population of the study involves people with Parkinson's disease, considering the inclusion and exclusion criteria for selection. The non-probabilistic sampling of the study points to the convenience recruitment of the population, based on publicity in the media and contact with the health department of municipalities and associations.

6.5 Eligibility Criteria

6.5.1 Inclusion Criteria

As inclusion criteria, the study participant must:

1) present a diagnosis of Parkinson's disease and be at stage 1-3 on the Hoehn and Yahr scale, consistent with mild and moderate disability, with the aim of homogenizing the sample in less advanced stages of PD.

2) be between 20 years old and 59 years old;

3) stable use of medications;

4) do not present cognitive impairment or dementia, having a score greater than 26 points on the Montreal Cognitive Assessment (MoCA);

5) be minimally capable of imagining motor activities, presenting a minimum score of 20 points on the Kinesthetic and Visual Imagery Questionnaire (KVIQ-10) (MALOUIN et al., 2007);

6) present motor alterations in the dominant upper limb, verified with the 9-Hole Peg Test (9HPT) above the average time according to sex and manual dominance, being: men 21.1 seconds for the dominant hand and 22.3 seconds for the non-dominant hand; for women with PD 19.9 seconds with the dominant hand and 21.4 seconds with the non-dominant hand (EARHART et al., 2011);

7) Have signed the Free and Informed Consent Form.

6.5.2 Exclusion criteria

As exclusion criteria, the study participant: do not present additional disorders of the central nervous system or other conditions that may affect the function of the upper and lower extremities; present other uncontrolled chronic conditions, which may interfere with the participant's safety.

6.6 Assessment instruments

The four instruments mentioned below will be applied to the inclusion criteria of participants in the study.

6.6.1 Hoehn and Yahr Scale

The Hoehn and Yahr Scale addresses five stages to assess the severity of PD. Having the advantage of ease of application and simplicity, it is also used mainly to define inclusion and exclusion criteria (GOETZ et al., 2004). The scale assesses: postural instability, rigidity, tremor and bradykinesia. Classifications 1, 2 and 3 refer to mild to moderate disability and stages 4 and 5 refer to more severe disabilities. For the study, stages 1-3 on the Hoehn and Yahr scale will be considered for inclusion criteria (Appendix 1).

6.6.2 *Montreal Cognitive Assessment*

The Montreal Cognitive Assessment (MoCA) is considered the most appropriate instrument for screening mild cognitive impairment and dementia in PD, since in people with PD it is sensitive to small cognitive changes (VÁZQUEZ et al., 2019). The MoCA is made up of eight cognitive domains scored from zero to 30 points and has an application time of around ten minutes. The domains are: visual and spatial skills, executive function, language, memory, attention and orientation, calculation and abstraction (MEMÓRIA et al., 2012) (Appendix 2). The MoCA has a good test-retest effect, low inter-rater variability, sensitivity of 82% and specificity of 75% relative to other tests for detecting mild cognitive impairment and dementia using a cutoff of 26 points.

6.6.3 Kinesthetic and Visual Imagery Questionnaire

The Kinesthetic and Visual Imagery Questionnaire (KVIQ-10) is a reliable and valid test to identify the capacity for imagination in people with PD (DEMANBORO et al., 2018). It is easy to administer and the movements, both real and imaginary, are appropriate for individuals with neuropathology (RANDHAWA; HARRIS; BOYD, 2010; HEREMANS et al., 2011). The KVIQ-10 version is a 10-item questionnaire that assesses visual (5 items) and kinesthetic (5 items) imagination capacity (MALOUIN et al., 2007).

The movement is first described, then demonstrated, and then the participant is asked to perform the movement, imagine it (using a first-person perspective), and then rate the clarity of the visual images or the intensity of the sensations. associated with an image of movement. The movements are: shoulder flexion, opposing the thumb with the

other fingers, forward trunk flexion, hip abduction, foot tapping. A visual scale is used ranging from 1 (“no image/sensation”) to 5 (“image as clear as seeing/as intense as performing the action”) (Appendix 3).

The total score is 50 points. As for the result, we have: higher scores, greater capacity for imagination (RANDHAWA; HARRIS; BOYD, 2010; HEREMANS et al., 2011). Thus, the KVIQ-10 will be used as an inclusion criteria instrument, considering a minimum score of 20 points, as a minimum capacity for imagination is expected to participate in the study. During the test, it will be recorded whether the side is dominant or non-dominant.

6.6.4 *9-Hole Peg Test*

The 9-Hole Peg Test (9HPT) involves a peg insertion task that allows correlation with the severity and duration of PD by assessing upper extremity function (EARHART et al., 2011). The task requires precise and coordinated finger movements, with the participant quickly picking up nine small pegs from a container, one at a time, placing them in holes on a board, and then moving them back to the table. container (MATHIOWETZ et al., 1985).

The average time considered to complete the 9HPT varies depending on the stage of PD (Appendix 4), considering the Hoehn and Yahr scale, and for each stage there is an average time to complete the task (EARHART et al., 2011). However, for the study, considering as inclusion criteria, there is a score according to sex and manual dominance, and must present a longer time than indicated, precisely to identify that there is impairment in manual dexterity.

Therefore, for men with PD there are: 21.1 seconds for the dominant hand and 22.3 seconds for the non-dominant hand; for women with PD 19.9 seconds with the dominant hand and 21.4 seconds with the non-dominant hand (EARHART et al., 2011). For selection criteria, the test will be carried out to check whether there is motor impairment on the dominant side. In addition to use for selection criteria, the 9HPT will also be used in pre- and post-testing on the dominant side.

The four instruments below will be used pre and post study intervention to assess motor and non-motor symptoms. An initial assessment, prepared for the research itself, will also be completed, with personal information about the participants and their PD (Appendix 1). The initial assessment includes indicators of the perception of motor

symptoms in the dominant upper limb and cognition by the participants themselves who will be questioned in the three test phases.

6.6.5 Movement Disorders Society Unified Parkinson's Disease Rating Scale

The gold standard instrument for assessing PD is the Movement Disorders Society Unified Parkinson's Disease Rating Scale (MDS-UPDRS) test, which contains four parts: 1) non-motor aspects of daily life experiences; 2) motor aspects of daily life experiences; 3) motor assessment; and 4) motor complications (GOETZ et al., 2004). For the study, the third part (UPDRS-III) will be considered, which involves motor assessment, but the following items, speech and facial expression, as well as the items corresponding to motor symptoms in the lower limbs will not be considered.

Therefore, the following items will be considered: postural tremor and kinetic tremor in the hands, rigidity, continuous finger tapping, hand movement and rapid alternating hand movements; in addition to the amplitude of the resting tremor and the persistence of the resting tremor for the upper limbs. This allows us to evaluate: speed, amplitude, hesitations, interruptions and decreases in amplitude, in addition to postural and kinetic tremor of the hands and amplitude and persistence of tremor at rest (GOETZ et al., 2004). For each of the eight items that will be considered, a value between 0 and 4 points can be assigned. For the maximum possible score, the higher the score, the worse the symptoms. As there are eight total items, the total score in part III of the MDS-UPDRS is 32 points (Appendix 5).

6.6.6 *Test D'évaluation Des Membres Supérieurs Des Personnes Âgées*

Considering the importance of evaluating the limitations of the upper limbs with tests or tasks representative of daily life, including the use of objects, the "Test d'Évaluation des Membres Supérieurs of Personnes Âgées" (TEMPA) was chosen (DE FREITAS et al. , 2017). TEMPA involves unilateral and bilateral tasks and a variety of real objects for evaluating quantitative (quotation of execution speed in tenths of a second) and qualitative (functional and task analysis scores) parameters.

There are eight functional tasks, four of which are bilateral: opening a pot and taking out a spoonful of coffee, unlocking a lock and opening a container of pills, writing on an envelope and sticking a stamp on it, tying a scarf around your neck, and shuffle and

deal playing cards; and four tasks are unilateral: picking up and carrying a jar; picking up a jug and pouring water into a glass, handling coins and picking up and moving small objects (NEDELEC et al., 2011).

The functional score is according to a four-level scale: 0, the task was completed successfully without hesitation or difficulty; 1, some difficulty or hesitation in completing the task; 2, the task was partially performed or certain steps were performed with significant difficulty; modified part of the task or need for assistance; and 3, was unable to complete the task, even with assistance. Thus, the value of the total functional score represents the sum of the right unilateral tasks (0 to 12), left (0 to 12) and bilateral tasks (0 to 12) and, therefore, can vary from 0 to 36 points (DE FREITAS et al., 2017).

The task analysis quantifies the difficulty encountered according to five items referring to the sensory and motor skills of the upper limb: strength, range of movement, precision of large movements, grasping and precision of fine movements (NEDELEC et al., 2011). Thus, the sum is performed for the five dimensions of the task analysis, ranging from 0 to 150 points. Remember that fine motion accuracy is not rated for tasks 1-3, and strength is not rated for tasks 5-8. The total score represents the sum of the functional grading and task analysis, totaling 186 points (Appendix 6). The assessment will be carried out pre and post intervention, and unilateral tasks will be performed only with the dominant upper limb. For a better interpretation of the assessment, especially when analyzing the task, it will be recorded for later analysis.

6.6.7 Parkinson's Disease-Cognitive Rating Scale

The Parkinson's Disease Cognitive Rating Scale (PD-CRS) arises from the need for a more comprehensive approach to the cognitive assessment of impaired fronto-subcortical and cortical functions throughout PD (PAGONABARRAGA et al., 2008). The PD-CRS can help identify people with PD in need of more specific assessments and care, differentiating those who have PD with cognitive impairment from those who have PD and are cognitively intact (ROSCA; SIMU, 2020).

In PD-CRS, nine functions are assessed, seven involving the "frontal-subcortical" function: sustained attention, working memory, immediate and delayed recall of verbal memory (list of words), alternating verbal fluency, verbal fluency of actions and clock drawing spontaneous; and two involving the "posterior cortex": naming twenty figures by visual comparison, added to the copy of the clock drawing (Appendix 7).

Administration time is approximately 20 minutes and is in the public domain, with training available. The total PD-CRS score is 134 points, with the ideal cut-off score being 81 points, and a score less than or equal to 64 points indicates PD with dementia (ROSCA; SIMU, 2020).

6.6.8 Canadian Occupational Performance Measure

The Canadian Occupational Performance Measure (COPM) is a semi-structured interview that allows the identification of activities that are important, but that there is difficulty in carrying out or are unable to carry out, called problem activities (DEDDING et al., 2004). COPM considers the areas of occupational performance: self-care, productivity and leisure. The importance score varies from 1 to 10 points. The most important problem activities (up to five of them) will also be scored based on performance: 1 point as “unable to do” up to 10 points as “able to do extremely well”; and also regarding satisfaction: 1 point for “not at all satisfied” and 10 points for “extremely satisfied”. In the end, there is an average value of performance and satisfaction. For the study, the values of the first assessment (before the intervention) and the second assessment (after the intervention) will be considered, taking the average sums of the scores for the number of problem activities mentioned (Appendix 8).

6.7 Outcomes

6.7.1 Main Outcome

As the main outcome of the study, it is expected to provide clinical evidence that action observation and motor imagination interventions have a positive impact on motor symptoms of the upper limbs, especially the improvement in the TEMPA score.

6.7.2 Secondary Outcome

As a secondary outcome of the study, I expect improvement in motor symptoms of PD using the UPDRS-III, and cognitive symptoms of PD using the Parkinson's Disease Cognitive Rating Scale (PD-CRS), and the impact of interventions on occupational

performance, with improving performance and satisfaction in carrying out daily activities through COPM.

6.8 Data collection procedure

6.8.1 Study Stages

After selecting participants, there will be randomization to allocate them to groups. The allocation will be through the Randomizer program, considering a random assignment in blocks for an equal chance for all participants, with the assignment in each of the five possibilities of the experimental condition, randomly separating them into five experimental groups (GE).

The study data collection stages will involve the pre-test (T0), the 10 intervention sessions, the immediate post-test (T1) and the test after a period of four weeks without intervention for follow-up (T2). All GE will be evaluated in the three stages corresponding to the study tests (T0, T1 and T2). Test application time will be around 30 to 45 minutes. T2 will be carried out through telephone contact to ask about the perception in relation to the dominant upper limb and cognition, as well as the reassessment of the COPM in relation to performance and satisfaction.

The study is single-blind, as the researcher will carry out the intervention protocol with the participants, but will not participate in the T0, T1 and T2 testing stages. These steps will be carried out by a group of research project participants who will be trained by the researcher to apply the instruments.

6.8.2 Intervention Proposal

The GE will be formed according to the proposed intervention: motor imagination, observation of action and motor execution (GE1), motor imagination and execution of action (GE2), observation of action and execution of action (GE3), motor imagination, execution of action and exoskeleton (GE4) and observation of action, execution of action and exoskeleton (GE5).

Regarding interventions, a protocol will be drawn up so that applicability occurs equally for all participants, both motor imagination (MI) and action observation (OA), in addition to the execution of the action (EA), as well as of the exoskeleton. The choice of

actions imagined, observed and executed will be based on activities in three areas of occupational performance: self-care, productivity and leisure. Thus, the applicability of the protocol will involve an approach based on occupations carried out in accordance with the COPM such as: personal care, mobility, independence for self-care; work and household chores for productivity; and quiet recreation, active recreation, and leisure socialization. The selection of such occupations makes the protocol centered on the needs of each person based on what they report is important, being of a more unique modality.

For IM, audios describing the actions will be recorded. Videos of the actions will be recorded for the OA. The execution of the action will be the same activities imagined and observed. The orientation for performing IM, OA and EX is in the sitting position, whereas for EA, depending on the action to be performed, it can be sitting or standing. During MI, the participant must remain with their eyes closed. The actions for imagination will be conducted in the first person, in which you imagine yourself performing the action (kinesthetic image) (SILVA et al., 2016). It is important that the environment does not have any type of interference that could cause interruptions. There is the possibility of applicability of interventions in groups or individually. The interventions of all groups will be an intensive approach of 10 continuous sessions, with a two-day break halfway through the intervention, totaling two weeks, with each session lasting 60 minutes per day. It is worth noting that, in order to understand all the interventions, an experiment will be carried out at T0 so that there are no doubts.

a) motor imagination, observation of the action and execution of the action (GE1): in a sitting position, the participant will watch the recorded video to observe the action, lasting 10 minutes. Soon after, you will close your eyes, and receive a description of the action, through recorded audio of the same previous action, to imagine it, lasting 10 minutes. In a sitting or standing position, depending on the action to be performed, the participant must perform the same action imagined previously, for 10 minutes. Each service session will have two different actions, thus totaling 60 minutes of intervention.
b) motor imagination and execution of the action (GE2): in a sitting position, the participant will close their eyes, and will receive a description of the action, through recorded audio, to imagine it, lasting 10 minutes. Therefore, in a sitting or standing position, depending on the action to be performed, the participant must perform the same action imagined previously, for 10 minutes. Each service session will have three different actions, thus totaling 60 minutes of intervention.

c) observation of the action and execution of the action (GE3): in a sitting position, the participant will watch the recorded video to observe the action, lasting 10 minutes. Therefore, in a sitting or standing position, depending on the action to be performed, the participant must perform the same action observed previously, for a period of 10 minutes. Each service session will have three different actions, thus totaling 60 minutes of intervention.
d) motor imagination, execution of the action and exoskeleton (GE4): in a sitting position, the participant will close their eyes, and will receive a description of the action, through recorded audio, to imagine it, lasting 10 minutes. In a sitting or standing position, depending on the action to be performed, the participant must perform the same action imagined previously, for 10 minutes. Then, you will perform the exoskeleton protocol for 40 minutes. Thus totaling 60 minutes of intervention.
e) observation of the action, execution of the action and exoskeleton (GE5): in a sitting position, the participant will watch the recorded video to observe the action, lasting 10 minutes. Therefore, in a sitting or standing position, depending on the action to be performed, the participant must perform the same action observed previously, for a period of 10 minutes. Then, you will perform the exoskeleton protocol for 40 minutes. Thus totaling 60 minutes of intervention.

As stated in the criteria, participants will be using dopaminergic medication normally, thus carrying out the data collection steps in the “on” period of the medication. Therefore, participants will also be invited to maintain all activities normally carried out during the study.

6.9 Data Analysis

The results of the qualitative variables will be presented as frequency and percentage, of the quantitative variables as symmetrical in mean and standard deviation and of the asymmetrical variables as median and interquartile range [P25 - P75]. Normality will be verified by the Shapiro-Wilk test. The groups will be compared regarding characteristics using the Chi-Square test, ANOVA and/or Kruskal-Wallis test with Tukey test for multiple comparisons.

Mixed ANOVA of repeated measures will be used or, in the absence of normality and/or presence of missings, generalized estimating equations (GEE) models will be used

to evaluate the main effects of group and moment and the group*moment interaction, with a matrix of unstructured correlation. The distributions that best fit the data (normal or gamma) will be evaluated using the AIK criterion, with the identity or logarithmic link functions, respectively. The Sidak test will be used for multiple comparisons. The results will be presented as mean and 95% CI. The analyzes will be performed using SPSS statistical software (IBM SPSS Statistics for Windows, Version 25.0. Armonk, NY: IBM Corp.). The significance level adopted will be 0.05.

6.10 Sample Calculation

The sample was estimated to find a non-negligible and useful effect size on the main outcome TEMPA, corresponding to Cohen's d=0.4 (f=0.2), when applied ANOVA, with a significant effect of the interaction (moment*intervention), suggested by Brysbaert (2019). Considering two evaluations, five groups, significance level of 0.05 with 80% power and 19% added for losses, 95 participants will be needed, 19 per group.

6.11 Ethical and legal procedures

Based on the recommendations of Resolution no. 466 of the National Health Council (BRASIL, 2012), involving research with human beings, the research project will be submitted for consideration by the Research Ethics Committee (CEP-UFCSPA). The researchers will undertake to maintain the confidentiality and anonymity of all participants who will be included in the study. Each research participant will receive the Free and Informed Consent Form (TCLE) (Appendix 2), which will inform the purposes of the research and risks associated with their participation, in easy and accessible language, in addition to clarifying that the study is voluntary and may be abandoned at any time without causing any harm. Data collection will only begin after the project is approved by the CEP.

6.12 Risks and Benefits

The research involves minimal risks to participants. The use of the instruments may cause some discomfort or embarrassment or fatigue during application, as well as during the interventions. If any type of situation occurs that poses a risk to the participant,

the participant may temporarily suspend participation in the research, or withdraw from it at any stage, without causing any harm and will be monitored until resolution.

As benefits, the research intends to investigate the association of the effects of motor imagination and action observation on motor changes in upper limbs and cognitive changes in PD. If the study hypotheses point to a positive possibility of both interventions, there will be a condition of improvement in motor and cognitive symptoms, benefiting the participants in their health condition. As a result, occupational performance can be improved in relation to carrying out daily activities. The participant will also receive an opinion on the results of the tests, having knowledge of their motor and cognitive conditions.

7 ACTIVITY SCHEDULE

2022

**ACTIVITY / MONTH**	**J**	**F**	**M**	**A**	**M**	**J**	**J**	**A**	**S**	**O**	**N**	**D**
Literature review	X	X	X									
Research project development	X	X	X	X	X	X						
Thesis qualification							X					
Submission to CEP-UFCSPA							X	X				
Training of research collaborators								X	X	X	X	

2023

**ACTIVITY / MONTH**	**J**	**F**	**M**	**A**	**M**	**J**	**J**	**A**	**S**	**O**	**N**	**D**
Participant recruitment	X	X										
Data collection	X	X	X	X	X	X	X	X	X	X	X	X

2024

**ACTIVITY / MONTH**	**J**	**F**	**M**	**A**	**M**	**J**	**J**	**A**	**S**	**O**	**N**	**D**
Data analysis	X	X										
Article development		X	X	X	X	X	X	X	X	X		
Submission of the Partial Report									X			

2025

**ACTIVITY / MONTH**	**J**	**F**	**M**	**A**	**M**	**J**	**J**	**A**	**S**	**O**	**N**	**D**
Thesis writing	X	X	X	X	X							
Thesis defense								X				

2026

ACTIVITY / MONTH	J	F	M	A	M	J	J	A	S	O	N	D
Submission of the Final Report						X						

8 BUDGET

The materials used to carry out the research are self-funded, with expenses being the responsibility of the researchers.

EXPENDITURE ELEMENT	AMOUNT (unit)	VALUE (in reais)
CONSUMER MATERIALS		
4 WHITE CRAFT PAPER 75G WITH 100 SHEETS	10	150,00
ballpoint pen 1.0mm blue crystal	10	10,00
small desktop stapler gp1000 26/6 for 20fls	1	12,00
clamp 26/6 galvanized 5,000 units	1	3,00
PERMANENT EQUIPMENT AND MATERIALS		
Inspiron 13 Notebook	1	5.749,00
Exoskeleton Equipment	1	10.000,00
TOTAL		15.924,00

REFERENCES

ABBRUZZESE, Giovanni; AVANZINO, Laura; MARCHESE, Roberta; PELOSIN, Elisa. Action Observation and Motor Imagery: Innovative Cognitive Tools in the Rehabilitation of Parkinson's Disease. **Parkinsons Dis**. v. 2015, n. 124214. 2015. doi: 10.1155/2015/124214.

ABBRUZZESE, Giovanni; MARCHESE, Roberta; AVANZINO, Laura; PELOSIN, Elisa. Rehabilitation for Parkinson's disease: Current outlook and future challenges. **Parkinsonism Relat Disord.** v. 22, supl. 1, p. S60-S64, Jan. 2016. doi: 10.1016/j.parkreldis.2015.09.005.

ABRAHAM, Amit; HART, Ariel; ANDRADE, Isaac; HACKNEY, Madeleine E. Dynamic Neuro-Cognitive Imagery Improves Mental Imagery Ability, Disease Severity, and Motor and Cognitive Functions in People with Parkinson's Disease. **Neural Plast.** v. 2018, 6168507. Mar. 2018. doi: 10.1155/2018/6168507.

ABRAMI, Avner; HEISIG, Stephen; RAMOS, Vesper; THOMAS, Kevin C; HO, Bryan K.; CAGGIANO, Vittorio. Using an unbiased symbolic movement representation to characterize Parkinson's disease states. **Scientific Reports**. v. 10, n. 7377, Apr. 2020. doi: 10.1038/s41598-020-64181-3.

ALIMARDANI, M.; NISHIO, S.; ISHIGURO, H. The importance of visual feedback design in BCIs; from embodiment to motor imagery learning. **PLoS ONE.** v. 11, n. e0161945, 2016.

ALMEIDA, Kelson James; CARVALHO, Larissa Clementino Leite de Sá; MONTEIRO, Tomásia Henrique Oliveira de Holanda; JÚNIOR, Paulo Cesar de Jesus Gonçalves; CAMPOS-SOUSA, Raimundo Nonato. Cut-off points of the Portuguese version of the Montreal Cognitive Assessment for cognitive evaluation in Parkinson’s disease. **Dementia & Neuropsychologia** [online]. v. 13, n. 2, p. 210-215. 2019. doi: 10.1590/1980-57642018dn13-020010.

ANG, K.K.; CHUA, K.S.; PHUA, K.S.; WANG, C.; CHIN, Z.Y.; KUAH, C.W.; LOW, W.; GUAN, C. A randomized controlled trial of EEG-based motor imagery brain-computer interface robotic rehabilitation for stroke. **Clin EEG Neurosci.** v. 46, p. 310–320. 2015.

ANG, K.K.; GUAN, C.; CHUA, K.S.; ANG, B.T.; KUAH, C.W.; WANG, C.; PHUA, K.S.; CHIN, Z.Y.; ZHANG, H. A large clinical study on the ability of stroke patients to use an EEG based moto imagery brain-computer interface. **Clinical EEG and Neuroscience.** v. 42, n. 4, p. 253–258. 2011.

AVANZINO, Laura; PELOSIN, Elisa; MARTINO, Davide; ABBRUZZESE, Giovanni. Motor timing deficits in sequential movements in Parkinson disease are related to action planning: a motor imagery study. **PLoS One.** v. 8, n. 9, Sept. 2013. DOI: 10.1371/journal.pone.0075454.

BANIQUED, P.D.E.; STANYER, E.C.; AWAIS, M.; ALAZMANI, A.; JACKSON, A.E.; MON-WILLIAMS, M.A.; MUSHTAQ, F.; HOLT, R.J. Brain-computer interface

robotics for hand rehabilitation after stroke: a systematic review. **J Neuroeng Rehabil.** v. 23, n. 8(1) 2021. doi: 10.1186/s12984-021-00820-8.

BARBOSA, Alessandra Ferreira; CHEN, Janini; FREITAG, Fernanda; VALENTE, Debora; SOUZA, Carolina de Oliveira; VOOS, Mariana Callil; CHIEN, Hsin Fen. Gait, posture and cognition in Parkinson’s disease. **Dement Neuropsychol.** v. 10, n. 4, p. 280-286, Dec. 2016. DOI: 10.1590/S1980-5764-2016DN1004005.

BEK, Judith; GOWEN, Emma; VOGT, Stefan; CRAWFORD, Trevor J.; POLIAKOFF, Ellen. Combined action observation and motor imagery influences hand movement amplitude in Parkinson's disease. **Parkinsonism & Related Disorders**. v. 61, p. 126-131. 2018. DOI: 10.1016/j.parkreldis.2018.11.001.

BEK, Judith; WEBB, Jordan; GOWEN, Emma; VOGT, Stefan; CRAWFORD, Trevor J.; SULLIVAN, Matthew S.; POLIAKOFF, Ellen. Patients' Views on a Combined Action Observation and Motor Imagery Intervention for Parkinson's Disease. **Parkinsons Dis.** v. 2016:7047910. 2016. DOI: 10.1155/2016/7047910.

BEK, Judith; HOLMES, Paul S.; CRAIG, Chesney E.; FRANKLIN, Zoe C.; SULLIVAN, Matthew; WEBB, Jordan, CRAWFORD, Trevor J.; VOGT, Stefan; GOWEN, Emma; POLIAKOFF, Ellen. Action Imagery and Observation in Neurorehabilitation for Parkinson's Disease (ACTION-PD): Development of a User-Informed Home Training Intervention to Improve Functional Hand Movements. **Parkinsons Dis.** v. 23:4559519, Jul. 2021. DOI: 10.1155/2021/4559519.

BIRBAUMER, N.; COHEN, L.G. Brain-computer interfaces: communication and restoration of movement in paralysis. **J Physiol**. v. 579, p. 621–636. 2007.

BONASSI, Gaia; LAGRAVINESE, Giovanna; BISIO, Ambra; RUGGERI, Piero; PELOSIN, Elisa; BOVE, Marco; AVANZINO, Laura. Consolidation and retention of motor skill after motor imagery training. **Neuropsychologia.** v. 143: 107472. 2020 DOI.org/10.1016/j.neuropsychologia.2020.107472.

BRASIL. Ministério da Saúde. Conselho Nacional de Saúde. Resolução n. 466, de 12 de dezembro de 2012.

BRAUN, Susy; BEURSKENS, Anna; KLEYNEN, Melanie; SCHOLS, Jos; WADE, Derick. Rehabilitation with mental practice has similar effects on mobility as rehabilitation with relaxation in people with Parkinson’s disease: a multicentre randomised trial. **J Physiother.** v. 57, n. 1, p. 27-34. 2011. DOI: 10.1016/S1836-9553(11)70004-2

BRYSBAERT, M. How Many Participants Do We Have to Include in Properly Powered Experiments? A Tutorial of Power Analysis with Reference Tables. **Journal of Cognition.** v. 2, n. 1:16, p. 1-38. Jul. 2019. DOI: 10.5334/joc.72. PMID: 31517234; PMCID: PMC6640316.

BUCCINO, Giovanni. Action observation treatment: a novel tool in neurorehabilitation. **Philosophical Transactions of the Royal Society**. v. 369(1644). 2014. DOI: 10.1098/rstb.2013.0185

BUCH, E.; WEBER, C.; COHEN, L.G.; et al. Think to move: a neuromagnetic brain-computer interface (BCI) system for chronic stroke. **Stroke**. v. 39, p. 910–917. 2008.

CALIGIORE, Daniele; MUSTILE, Magda; SPALLETTA, Gianfranco; BALDASSARRE, Gianluca. Action observation and motor imagery for rehabilitation in Parkinson's disease: A systematic review and an integrative hypothesis. **Neurosci Biobehav Rev.** v. 72, p. 210-222. 2017. DOI: 10.1016/j.neubiorev.2016.11.005.

CHEN, Y.; YANG, W.; LONG, J.; ZHANG, Y.; FENG, J. et al. Discriminative Analysis of Parkinson's Disease Based on Whole-Brain Functional Connectivity. PLOS ONE v. 10, n. 4, 2015. DOI: 10.1371/journal.pone.0124153

CIKAJLO, Imre; POTISK, Karmen Peterlin. Advantages of using 3D virtual reality based training in persons with Parkinson's disease: a parallel study. **J Neuroeng Rehabil.** v. 16. Oct. 2019. DOI: 10.1186/s12984-019-0601-1.

DEDDING, Christine; CARDOL, Mieke; EYSSEN, Isaline C.J.M.; DEKKER, Joost; BEELEN, Anita. Validity of the Canadian Occupational Performance Measure: a client-centred outcome measurement. **Clin Rehabil.** v. 18, n. 6, p. 660-667. Sept. 2004. DOI: 10.1191/0269215504cr746oa.

DE FREITAS, Paula Ruiz; LEMOS, Ana Elisa; SANTOS, Mariana Palla; MICHAELSEN, Stella Maris; CORRÊA, Clynton Lourenço; SWAROWSKY, Alessandra. "Test D'évaluation Des Membres Supérieurs Des Personnes Âgées" (TEMPA) to assess upper limb activity in Parkinson's disease. **J Hand Ther.** v. 30, n. 3, p. 320-327, Jul-Sept. 2017. DOI: 10.1016/j.jht.2016.07.003.

DEMANBORO, Alan; STERR, Annette; DOS ANJOS, Sarah Monteiro; CONFORTO, Adriana Bastos. A Brazilian-Portuguese version of the Kinesthetic and Visual Motor Imagery Questionnaire. **Arquivos de Neuro-Psiquiatria.** v. 76, n. 1, p. 26-31. 2018. DOI: 10.1590/0004-282X20170181.

DI RIENZO, Franck; COLLET, Chhristian; HOYEK, Nady; GUILLOT, Aymeric. Impact of neurologic deficits on motor imagery: a systematic review of clinical evaluations. **Neuropsychol Rev.** 2014 Jun;24(2):116-47. DOI: 10.1007/s11065-014-9257-6.

DICKSON, J.M.; GRU¨NEWALD, R.A. Somatic symptom progression in idiopathic Parkinson's disease. **Parkinsonism Relat Disord.** v. 10, p. 487-492. 2004.

EARHART, Gammon M.; CAVANAUGH, Jim T.; ELLIS, Terry; FORD, Matt P.; FOREMAN, K. Bo; DIBBLE, Lee. The 9-hole PEG test of upper extremity function: average values, test-retest reliability, and factors contributing to performance in people with Parkinson disease. **J Neurol Phys Ther.** v. 35, n. 4, p. 157-163. Dec. 2011. DOI: 10.1097/NPT.0b013e318235da08.

EL-WISHY, Abeer Abo Bakr; FAYEZ, Eman. Effect of Locomotor Imagery Training Added to Physical Therapy Program on Gait Performance in Parkinson Patients: A

Randomized Controlled Studyy. **Egypt J Neurol Psychiat Neurosurg.** v. 50, n. 1, p. 31-37. 2013.

ENDO, Takuyuki; YOKOE, Masaru; FUJIMURA, Harutoshi; SAKODA, Saburo. **Novel methods to evaluate symptoms in Parkinson's disease**—rigidity and finger tapping. In: Diagnostics and Rehabilitation of Parkinson's Disease, edited by J. Dushanova. Rijeka: InTech, 2011:91–206. https://doi.org/10.5772/17967

FERREIRA, Renilson Moraes; ALVES, Wilson Mateus Gomes da Costa; DE LIMA, Tiago Alencar; ALVES, Thiago Goçalves Gibson; ALVES FILHO, Pedro Arthur Madureira; PIMENTEL, Clebson Pantoja; SOUSA, Evitom Correa; CORTINHAS-ALVES, Erik Artur. The effect of resistance training on the anxiety symptoms and quality of life in elderly people with Parkinson's disease: a randomized controlled trial. Arq Neuropsiquiatr. v. 76, n. 8, p. 499-506. Aug. 2018. DOI: 10.1590/0004-282X20180071.

FERREIRA-SÁNCHEZ, María del Rosario; MORENO-VERDÚ, Marcos; CANO-DE-LA-CUERDA, Roberto. Quantitative Measurement of Rigidity in Parkinson´s Disease: A Systematic Review. **Sensors (Basel).** v. 6, n. 20(3):880. Feb. 2020. DOI: 10.3390/s20030880.

FÉRY, Yves-Andre. Differentiating visual and kinesthetic imagery in mental practice. **Can J Exp Psychol**. v. 57, n. 1, p. 1-10. 2003. DOI: 10.1037/h0087408.

FISCHER, Petra; POGOSYAN, Alek; CHEERAN, Binith; GREEN, Alexander L.; AZIZ, Tipu Z.; HYAM, Jonathan. et al. Subthalamic nucleus beta and gamma activity is modulated depending on the level of imagined grip force. **Exp Neurol.** v. 293, p. 53-61. Jul. 2017. DOI: 10.1016/j.expneurol.2017.03.015.

FLORIANO, Eduardo Nascimento; ALVES, Jacqueline Frazão; ALMEIDA, isabela Andrelino; SOUZA, Roger burgo; CHRISTOFOLETTI, Gustavo; SANTOS, Suhaila Mahmoud Smaili. Dual task performance: a comparison between healthy elderly individuals and those with Parkinson's disease. **Fisioterapia em Movimento** [online]. v. 28, n. 2, p. 251-258. 2015. DOI: 10.1590/0103-5150.028.002.AO05.

GATTI, R.; TETTAMANTI, A.; GOUGH, P.M.; RIBOLDI, E.; MARINONI, L.; BUCCINO, G. Action observation versus motor imagery in learning a complex motor task: a short review of literature and a kinematics study. **Neurosci Lett.** v. 12, n. 540, p. 37-42. Apr. 2013. DOI: 10.1016/j.neulet.2012.11.039.

GEBHARDT, A.; VANBELLINGEN, T.; BARONTI, F.; KERSTEN, B.; BOHLHALTER, S. Poor dopaminergic response of impaired dexterity in Parkinson's disease: bradykinesia or limb kinetic apraxia? **Mov Disord.** v. 2, p. 1701-1706. 2008.

GOETZ, Chistopher G.; POEWE, Werner; RASCOL, Olivier; SAMPAIO, Cristina; STEBBINS, Glenn T.; COUNSELL, Carl. et al. Movement Disorder Society Task Force report on the Hoehn and Yahr staging scale: status and recommendations. **Mov Disord.** v. 19, n. 9, p. 1020-1028. Sept. 2004. DOI: 10.1002/mds.20213.

HANSON, T.; FULLER, A.; LEBEDEV, M.; TURNER, D.; NICOLELIS, M. Subcortical Neuronal Ensembles: An Analysis of Motor Task Association, Tremor,

Oscillations, and Synchrony in Human Patients. **Journal of Neuroscience**. v. 32, n. 25, p. 8620-8632. 2012. DOI: 10.1523/JNEUROSCI.0750-12.2012.

RANDHAWA, Bubblepreet Kaur; HARRIS, Susan R.; BOYD, Lara A. The kinesthetic and visual imagery questionnaire is a reliable tool for individuals with Parkinson disease. **Journal of Neurologic Physical Therapy.** v. 34, n. 3, p. 161–167. 2010.

HELDMAN, Dustin A.; JANKOVIC, Joseph; VAILLANCOURT, David E.; PRODOEHL, Janey; ELBLE, Rodgre J.; GIUFFRIDA, Joseph P. Essential tremor quantification during activities of daily living. **Parkinsonism Relat. Disord.** v. 17, n. 7, p. 537–542. 2011. DOI: 10.1016/j.parkreldis.2011.04.017.

HELMICH, Rick C.; BLOEM, Bastiaan R.; TONI, Ivan. Motor imagery evokes increased somatosensory activity in Parkinson's disease patients with tremor. **Hum Brain Mapp.** v. 33, n. 8, p. 1763-1779. 2012. DOI: 10.1002/hbm.21318.

HELMICH, Rick C.; HALLETT, Mark; DEUSCHL, Gunther; TONI, Ivan; BLOEM, Bastiaan R. Cerebral causes and consequences of parkinsonian resting tremor: a tale of two circuits? **Brain.** v. 135, p. 3206-3226. Nov. 2012. DOI: 10.1093/brain/aws023.

HEREMANS, Elke; FEYS, Peter; NIEUWBOER, Alice; VERCRUYSSE, Sarah; VANDENBERGHE, Wim; SHARMA, Nikhil; HELSEN, Werner. Motor imagery ability in patients with early- and mid-stage Parkinson disease. **Neurorehabil Neural Repair.** v. 25, n. 2, p. 168-77. Feb. 2011. DOI: 10.1177/1545968310370750.

HEREMANS, Elke; NIEUWBOER, Alice; FEYS, Peter; VERCRUYSSE, Sarah; VANDENBERGHE, Wim; SHARMA, Nikhil; HELSEN, Werner. External cueing improves motor imagery quality in patients with Parkinson disease. **Neurorehabilitation and Neural Repair.** v. 26, n. 1, p. 27–35. 2012. DOI: 10.1177/1545968311411055.

HU, Zixiang; HAO, Manzhao; XU, Shaoqing; XIAO, Qin; LAN, Ning. Evaluation of tremor interference with control of voluntary reaching movements in patients with Parkinson's disease. **J Neuroeng Rehabil.** v. 16, n. 38. Mar. 2019. DOI: 10.1186/s12984-019-0505-0.

KANEKO, Naotsugu; YOKOYAMA, Hikaru; MASUGI, Yohei; WATANABE, Katsumi; NAKAZAWA, Kimitaka. Phase dependent modulation of cortical activity during action observation and motor imagery of walking: An EEG study. **Neuroimage.** 15;225:117486. Jan. 2017. DOI: 10.1016/j.neuroimage.2020.117486.

KANG, Minsoo; RAGAN, Brian G.; PARK, Jae-Hyeon. Issues in outcomes research: an overview of randomization techniques for clinical trials. **J Athlc Train.** v. 43, 2, p. 215-221. 2008. DOI: 10.4085/1062-6050-43.2.215.

KIKUCHI, A.; BABA, T.; HASEGAWA, T.; SUGENO, N.; KONNO, M.; MIURA, E.; OSHIMA, R.; AOKI, M.; TAKEDA, A. Improvement of Freezing of Gait in Patients with Parkinson's Disease by Imagining Bicycling. **Case Rep Neurol.** v. 6, p. 92-95. 2014. DOI: 10.1159/000362119

KOBAYASHI, Eiji; HIMURO, Nobuaki; MITANI, Yuji; TSUNASHIMA, Takuya; NOMURA, Kyouhei; CHIBA, Susumu. Feasibility and informativeness of the Canadian occupational performance measure for identifying priorities in patients with Parkinson's disease. **Physiotherapy Theory and Practice**. v. 6. Jan. 2022. DOI: 10.1080/09593985.2021.2023926.

LAWRENCE, E.S.; COSHALL, C.; DUNDAS, R. et al. Estimates of the prevalence of acute stroke impairments and disability in a multiethnic population. **Stroke.** v. 32, p. 1279-1284. 2001.

LI, M.; LIU, Y.; WU, Y.; LIU, S.; JIA, J.; ZHANG, L. Neurophysiological substrates of stroke patients with motor imagery-based brain-computer Interface training. **Int J Neurosci.** v. 124, p. 403–415, 2014.

MALLING, Anne Sofie Bogh; MORBERG, Bo Mohr; WERMUTH, Lene; GREDAL, Ole; BECH, Per; JENSEN, Bente Rona. The effect of 8 weeks of treatment with transcranial pulsed electromagnetic fields on hand tremor and inter-hand coherence in persons with Parkinson's disease. **J Neuroeng Rehabil.** v. 31, n. 16(1). Jan. 2019. DOI: 10.1186/s12984-019-0491-2.

MALOUIN, Francine; RICHARDS, Carol L.; JACKSON, Philip L.; LAFLEUR, Martin F.; DURAND, Anne; DOYON, Julien. The Kinesthetic and Visual Imagery Questionnaire (KVIQ) for assessing motor imagery in persons with physical disabilities: a reliability and construct validity study. **J Neurol Phys Ther.** v. 31, n. 1, p. 20-29. Mar. 2007. DOI: 10.1097/01.npt.0000260567.24122.64.

MATHIOWETZ, Virgil; WEBER, Karen; KASHMAN, Nancy; VOLLAND, Gloria. Adult Norms for the Nine Hole Peg Test of Finger Dexterity. **The Occupational Therapy Journal of Research.** v. 5, n. 1, p. 24-38. 1985. DOI: 10.1177/153944928500500102

MEHRHOLZ, J.; POHL, M.; PLATZ, T.; KUGLER, J.; ELSNER, B. Electromechanical and robot-assisted arm training for improving activities of daily living, arm function, and arm muscle strength after stroke. **Cochrane Database Syst Rev.** v. 7, n. 11. 2015. DOI: 10.1002/14651858.CD006876.pub4.

MEMAR, Sara; DELROBAEI, Mehdi; PIETERMAN, Marcus; MCISAAC, Kenneth; JOG, Mandar. Quantification of whole-body bradykinesia in Parkinson's disease participants using multiple inertial sensors. **J Neurol Sci.** v. 15, n. 387, p. 157-165. Apr. 2018. DOI: 10.1016/j.jns.2018.02.001.

MEMÓRIA, Cláudia M.; YASSUDA, Mônica S.; NAKANO, Eduardo Y.; FORLENZA, Orestes V. Brief screening for mild cognitive impairment: validation of the Brazilian version of the Montreal cognitive assessment. **Int J Geriatr Psychiatry**. v. 28, n. 1, p. 34-40. Jan. 2013. doi: 10.1002/gps.3787.

MILLER, K.J.; SCHALK, G.; FETZ, E.E.; DEN, N.I.J.S.M.; OJEMANN, J.G.; RAO, R.P. Cortical activity during motor execution, motor imagery, and imagery-based online feedback. **Proc Natl Acad Sci U S A.** v. 107, 2010.

MYERS, Peter S.; MCNEELY, Marie E.; PICKETT, Kristen A.; DUNCAN, Ryan P.; EARHART, Gammon M. Effects of exercise on gait and motor imagery in people with Parkinson disease and freezing of gait. **Parkinsonism Relat Disord.** v. 53, p. 89-95. 2018. DOI: 10.1016/j.parkreldis.2018.05.006.

MONGE-PEREIRA, E.; IBAÑEZ-PEREDA, J.; ALGUACIL-DIEGO, I.M.; SERRANO, J.I.; SPOTTORNO-RUBIO, M.P.; MOLINA-RUEDA, F. Use of Electroencephalography Brain-Computer Interface Systems as a Rehabilitative Approach for Upper Limb Function After a Stroke: A Systematic Review. **PM R.** v. 9, n. 9, p. 918-932. 2017. DOI: 10.1016/j.pmrj.2017.04.016.

NEDELEC, Bernadette; DION, Karyne; CORREA, José A.; DESROSIERS, Johanne. Upper extremity performance test for the elderly (TEMPA): normative data for young adults. **J Hand Ther.** v. 24, n. 1, p. 31-42. Jany-Mar. 2011. DOI: 10.1016/j.jht.2010.09.001.

NIELSEN, Christina; SIERSMA, Volkert; GHAZIANI, Emma; BEYER, Nina; MAGNUSSON, S. Peter; COUPPÉ, Christian. Health- Related Quality of Life and Physical Function in Individuals with Parkinson's Disease after a Multidisciplinary Rehabilitation Regimen-A Prospective Cohort Feasibility Study**. Int J Environ Res Public Health.** v. 17, n. 20. 2020. DOI: 10.3390/ijerph17207668.

OLDFIELD, R.C. The assessment and analysis of handedness: the Edinburgh inventory. **Neuropsychologia.** v. 9, n. 1, p. 97-113. 1971. DOI: 10.1016/0028-3932(71)90067-4.

PAGONABARRAGA, Javier; KULISEVSKY, Jaime; LLEBARIA, Gisela; GARCÍA-SÁNCHEZ, Carmen; PASCUAL-SEDANO, Berta; GIRONELL, Alexandre. Parkinson's disease-cognitive rating scale: a new cognitive scale specific for Parkinson's disease. **Mov Disord.** v. 15, n. 23(7), p. 998-1005. May. 2008. DOI: 10.1002/mds.22007.

PAUL, Serene S.; DIBBLE, Leland E.; OLIVIER, Genevieve N.; WALTER, Christopher; KEVIN, Duff; SCHAEFER, Sydney Y. Dopamine replacement improves motor learning of an upper extremity task in people with Parkinson disease. **Behav Brain Res.** v. 13. n. 377. Jan. 2020. DOI: 10.1016/j.bbr.2019.112213.

PELOSIN, Elisa; AVANZINO, Laura; BOVE, Marco; STRAMESI, Paola; NIEUWBOER, Alice; ABBRUZZESE, Giovanni. Action observation improves freezing of gait in patients with Parkinson's disease. **Neurorehabil Neural Repair.** v. 24, n. 8, p. 746-52. Oct. 2010. DOI: 10.1177/1545968310368685.

PELOSIN, Elisa; BOVE, Marco; RUGGERI, Piero; AVANZINO, Lauro; ABBRUZZESE, Giovanni. Reduction of bradykinesia of finger movements by a single session of action observation in Parkinson disease. **Neurorehabil Neural Repair.** v. 27, p. 552– 560. 2013. DOI: 10.1177/1545968312471905.

PFURTSCHELLER, G.; LOPES DA SILVA, F.H. Event-related EEG/MEG synchronization and desynchronization: basic principles. **Clin Neurophysiol.** v. 110, p. 1842–57. 1999.

PFURTSCHELLER, G.; NEUPER, C. Future prospects of ERD/ERS in the context of brain computer interface (BCI) developments. **Prog Brain Res**. v.159, p. 433–437. 2006.

PONDÉ, Priscilla de Dio Santos; RODRIGUES, Dayane Nunes; CRISTINA, Layane; BASTOS, Marta Ferreira; SANCHES, Iris Callado; GAMA, Eliane Florencio. Chronic responses of physical and imagery training on Parkinson's disease. **Revista Brasileira de Medicina do Esporte** [online]. v. 25, n. 6, p. 503-508. 2019. DOI: 10.1590/1517-869220192506214238.

POWELL, Douglas; HANSON, Nicholas; THRELKELD, A. Joseph; FANG, Xiang; XIA, Ruiping. Enhancement of parkinsonian rigidity with contralateral hand activation. **Clin Neurophysiol.** v. 122, n. 8, p. 1595-1601. 2011. DOI: 10.1016/j.clinph.2011.01.010.

POWELL, Douglas; THRELKELD, A. Joseph; FANG, Xiang; MUTHUMANI, A.; XIA, Ruiping. Amplitude- and velocity-dependency of rigidity measured at the wrist in Parkinson's disease. **Clin Neurophysiol.** v. 123, n. 4, p. 764-773. 2012. DOI: 10.1016/j.clinph.2011.08.004.

PROUD, E.L.; MORRIS, M.E. Skilled hand dexterity in Parkinson's disease: effects of adding a concurrent task. **Arch Phys Med Rehabil.** v. 91, p. 794-799. 2010.

ROSCA, Elena Cecilia; SIMU, Mihaela. Parkinson's Disease-Cognitive Rating Scale for Evaluating Cognitive Impairment in Parkinson's Disease: A Systematic Review. **Brain Sci.** v. 25. Aug. 2020. DOI: 10.3390/brainsci10090588.

ROSSI, F.; SAVI, F.; PRESTIA, A.; MONGARDI, A.; DEMARCHI, D.; BUCCINO, G. Combining Action Observation Treatment with a Brain-Computer Interface System: Perspectives on Neurorehabilitation. **Sensors (Basel).** v. 21, n. 24, 2021. DOI:10.3390/s21248504

SARASSO, Elisabetta; GEMMA, Mariano; AGOSTA, Federica; FILIPPI, Massimo; GATTI, Roberto. Action observation training to improve motor function recovery: a systematic review. **Arch Physiother.** v. 5, n. 14. 2015. DOI: 10.1186/s40945-015-0013-x

SANTIAGO, Lorena Marques de Melo; DE OLIVEIRA, Daniel Antunes; FERREIRA, Louise Gabriella Lopes de Macêdo; PINTO, Hyanne Yasmin de Brito; SPANIOL, Ana Paula. TRIGUEIRO, Larissa Coutinho de Lucena. et al. Immediate effects of adding mental practice to physical practice on the gait of individuals with Parkinson's disease: Randomized clinical trial. **NeuroRehabilitation.** v. 37, n. 2, p. 263-71. 2015. DOI: 10.3233/NRE-151259.

SHAWEN, Nicholas; O'BRIEN, Megan K.; VENKATESAN, Sanjeev; LONINI, Luca; SIMUNI, Tanya; HAMILTON, Jaime L.; GHAFFARI, Roozbeh; ROGERS, John A.; JAYARAMAN, A. Role of data measurement characteristics in the accurate detection of Parkinson's disease symptoms using wearable sensors. **J Neuroeng Rehabil.** v. 17, n. 52. Apr. 2020. DOI: 10.1186/s12984-020-00684-4.

SHULMAN, Joshua M.; JAGER, Philip L.; FEANY, Mel B. Parkinson’s disease: genetics and pathogenesis. **Annu Ver Pathol MechDis.** v. 6, p. 193-222. 2011. DOI: 10.1146/annurev-pathol-011110-130242.

SILVA, Douglas Monteiro; CORIOLANO, Maria das Graças Wanderley de Sales; MACÊDO, João Gabriel Figuêredo; SILVA, Liliane Pereira; LINS, Otávio Gomes. Practice of mental protocols used in rehabilitation of patients with Parkinson’s disease: a systematic review. **Acta Fisiatr**. v. 23, n. 3, p. 155-160. 2016. DOI: 10.5935/0104-7795.20160030

SOUSA, Nariana Mattos Figueiredo; MACEDO, Roberta Correa; BRUCKI, Sonia Maria Dozzi. Cross-sectional associations between cognition and mobility in Parkinson’s disease. **Dementia & Neuropsychologia** [online]. v. 15, n. 1, p. 105-111. 2021. DOI: 10.1590/1980-57642021dn15-010011.

STURKENBOOM, I.; THIJSSEN, M.; GONS-VAN ELSACKER, J. et al. Guidelines for occupational therapy in Parkinson’s disease rehabilitation. Nijmegen (The Netherlands)/Miami: ParkinsonNet/National Parkinson Foundation. 2011.

TAMIR, Ruth; DICKSTEIN, Ruth; HUBERMAN, Moshe. Integration of motor imagery and physical practice in group treatment applied to subjects with Parkinson’s disease. Neurorehabil Neural Repair. v. 21, n. 1, p. 68-75. 2007. DOI: 10.1177/1545968306292608

VAN DEN NOORT, Josien C.; VERHAGEN, Rens; VAN DIJK, Kess J.; VELTINK, Peter H.; VOS, Michelle C.P.M.; DE BIE, Rob M.A.; BOUR, Lo J.; HEIDA, Ciska T. Quantification of Hand Motor Symptoms in Parkinson's Disease: A Proof-of-Principle Study Using Inertial and Force Sensors. **Ann Biomed Eng.** v. 45, n. 10, p. 2423-2436. Oct. 2017. DOI: 10.1007/s10439-017-1881-x.

VÁSQUEZ, Krisly Arguedas; VALVERDE, Erick Miranda; AGUILAR, Daniel Valerio; GABARAIN, Henro-Jacques Hernádez. Montreal Cognitive Assessment scale in patients with Parkinson Disease with normal scores in the Mini-Mental State Examination. **Dement Neuropsychol.** v. 13, n. 1, p. 78-81. 2013. doi:10.1590/1980-57642018dn13-010008.

WOLPAW, J.R.; BIRBAUMER, N.; MCFARLAND, D.J. PFURTSCHELLER, G.; VAUGHAN, T.M. BRAINCOMPUTER interfaces for communication and control. **Clin Neurophysiol.** v. 113, p. 767–791. 2002.

ZHANG, J.J.Q.; FONG, K.N.K.; WELAGE, N.; LIU, K.P.Y. The activation of the mirror neuron system during action observation and action execution with mirror visual feedback in stroke: a systematic review. **Neural Plast.** v. 2018, p. 1-14. 2018. doi:10.1155/2018/2321045.

ZETTERBERG, H.; FRYKBERG, G.E.; GÄVERTH, J.; LINDBERG, P.G. Neural and nonneural contributions to wrist rigidity in Parkinson's disease: an explorative study using the NeuroFlexor**. Biomed Res Int.** v. 2015. 2015. DOI: 10.1155/2015/276182.

APPENDIX 1 – INITIAL ASSESSMENT

Name: ____
Date of Birth: ____________________ Sex: M ( ) F ( )
City of residence: ____
Contact telephone number: ____
Education: ____

Diagnosis Time: ______________________________________
Onset of symptoms (laterality): ________________________________
Associated Diseases:

Medication:
____
____

DP medication
schedules:____

Performs another type of rehabilitation: ( ) yes ( ) no
Which:
Frequency: ( ) 1x a week ( ) 2x a week ( ) 3x a week

Performs physical activity: ( ) yes ( ) no
Which:
Frequency: ( ) 1x a week ( ) 2x a week ( ) 3x a week

Modified Hoehn and Yahr Scale: 1( ) 2( ) 3( ) 4( ) 5( )

Mão Dominante: ( ) esquerda ( ) direita

9HPG - tempo de teste (segundos): ______________________

Mão Dominante: _______________

Perception:
1) How do you consider the severity of your upper limb motor functions (dexterity, speed, precision of movement)?
( ) None ( ) Little ( ) Average ( ) A lot ( ) Extreme

2) How do you consider the severity of your cognitive functions (memory, attention, concentration, organization)?
( ) None ( ) Little ( ) Average ( ) A lot ( ) Extreme

Appraiser: __________________________________
Date: ______________

APPENDIX 2 - FREE INFORMED CONSENT FORM

You are being invited to participate in a study coordinated by professor and researcher Fernanda Cechetti from the Federal University of Health Sciences of Porto Alegre (UFCSPA), called “EFFECTS OF MOTOR IMAGINATION AND ACTION OBSERVATION ON MOTOR CHANGES IN UPPER LIMBS AND COGNITIVE CHANGES IN PARKINSON’S DISEASE”. For those who live in Santa Maria and the region, the study will be carried out in partnership with the Federal University of Santa Maria (UFSM) and for those who live in Porto Alegre and the region, the study will be carried out in partnership with Clínica NeuroGold, for the provision of all materials necessary to carry out the tests and study treatment interventions.

The study aims to investigate the effects of motor imagination and action observation on motor changes in the upper limbs and cognitive changes in Parkinson's disease. The study is necessary because Parkinson's disease is quite common in adults and the elderly and has no cure, causing limitations in carrying out daily activities, as the person feels slower and has difficulty moving, in addition to having problems with memory, such as forgetfulness, and being inattentive when carrying out activities.

To this end, three motor assessments will be carried out: some items from the third part of the Movement Disorders Society Unified Parkinson's Disease Rating Scale (UPDRS-III) which only observe the performance of some movements; the Test d’Évaluation des Membres Supérieurs of Personnes Âgées (TEMPA) which involves performing tasks with one hand and with both hands and the 9-Hole Peg Test (9HPT) which consists only of the task of picking up and inserting pegs; and a cognitive assessment using the Parkinson’s Disease-Cognitive Rating Scale (PD-CRS), which are items for repeating words, naming pictures, saying words and drawing pictures. We will also ask about the activities that are important to you and that you are unable to carry out or have difficulty carrying out, and you will rate the way you carry them out and your satisfaction, with the Canadian Occupational Performance Measure (COPM). And also, how do you think the severity of the symptoms is in the function of the upper limb and cognitive functions (memory, attention, concentration and others). Test application time will be around 30 to 45 minutes.

After taking the tests above, which will be carried out at the beginning and end of the treatment, you will be asked to undergo a treatment lasting 10 days in total, five days from Monday to Friday, with Saturday and Sunday break, and then five days from

Monday to Friday. Each day of treatment lasts 60 minutes. After completing the 10-day treatment, you will be evaluated again, but by telephone, in which we will only ask about your performance and satisfaction in carrying out daily activities and the perception part, which is how you think the severity of the symptoms is. . After four weeks of the end of treatment, we will schedule a return visit to perform the tests again.

The study has five different treatment groups and you will only be in one of them. Let's explain each of them.

In group 1: while sitting, you will watch a video recorded to observe a common everyday activity, such as washing dishes, for 2 minutes. Soon after, with your eyes closed, you will hear a recorded audio describing the same activity as before, and you must imagine that activity for 2 minutes. Then you will carry out an imagined and observed activity. Each service encounter will have five different actions that will be repeated twice. Thus totaling 60 minutes of intervention.

In group 2: while sitting, you will close your eyes, listen to a recorded audio describing a common everyday activity, such as washing dishes, and you will have to imagine that activity for 2 minutes. Immediately afterwards, in a sitting or standing position, depending on the activity to be carried out, you must carry out the same activity that you imagined before. Each service encounter will have five different actions that will be repeated twice. Thus totaling 40 minutes of intervention.

In group 3: while sitting, you will watch a video recorded to observe a common everyday activity, such as washing dishes, for 2 minutes. Immediately afterwards, in a sitting or standing position, depending on the activity to be performed, you must perform the same activity that you watched in the video. Each service encounter will have five different actions that will be repeated twice. Thus totaling 40 minutes of intervention.

In group 4: while sitting, you will close your eyes, listen to a recorded audio describing a common everyday activity, such as washing dishes, and you will have to imagine that activity for 2 minutes. Immediately afterwards, in a sitting or standing position, depending on the activity to be carried out, you must carry out the same activity that you imagined before. Then, you will undergo treatment with the exoskeleton, which consists of placing a cap on your head and a robotic glove on the hand that you have the most difficulty with. You will receive instructions to imagine the same activities, and when you imagine, the glove will move your fingers. The time is 40 minutes. Thus totaling 60 minutes of intervention.

In group 5: while sitting, you will watch a video recorded to observe a common everyday activity, such as washing dishes, for 2 minutes. Immediately afterwards, in a sitting or standing position, depending on the activity to be performed, you must perform the same activity that you watched in the video, for 2 minutes. Then, you will undergo treatment with the exoskeleton, which consists of placing a cap on your head and a robotic glove on the hand that you have the most difficulty with. You will receive instructions to imagine the same activities, and when you imagine, the glove will move your fingers. The time is 40 minutes. Thus totaling 60 minutes of intervention.

The research may involve minimal risks, and the researchers are aware, in addition to being able to conduct the research safely and offer assistance if necessary, being fully responsible for any situation involving the research. The use of the instruments may cause some discomfort or embarrassment or fatigue during application, as well as during the interventions. If any type of situation occurs that causes risk, you can temporarily suspend participation in the research, or withdraw at any stage, without causing any harm and you will be accompanied until the situation is resolved, with compensation being guaranteed for any damages resulting from the research. As benefits, we are betting on a positive possibility of treatment, with improved arm and hand movements, remembering things and being more attentive, improving the way you carry out your daily activities. At the end of the research, you will also receive an opinion on the test results, providing you with knowledge of your motor and cognitive conditions.

The results obtained may be published in scientific journals in the study area, ensuring confidentiality, secrecy and privacy of your identity. Therefore, it is clear that you are free to refuse to participate or withdraw your consent, at any stage of the research without any penalty or personal harm. Any expenses you incur with transportation or food will be covered by the researchers, but you will not receive payment for participating in the research.

This document will be issued in two copies, one intended for you and the other for the researchers. If problems or questions arise about the study, you can contact the researchers using the telephone numbers provided at the end of the document.

I ____ was informed, in a clear and detailed manner, regarding the objectives of the research mentioned above. I received information about the evaluation and interventions that will be carried out and clarified my doubts. I know that at any time I can request new information and change my decision to participate

in the study, if I wish. Those responsible, Fernanda Cechetti, Kátine Marchezan Estivalet, assured me that all research data will be confidential and I will be free to withdraw from participation in this research, if I deem it necessary.

If you have questions and/or complaints about matters related to the research, please contact the researchers on the following telephone numbers: Fernanda Cechetti (51) 9 8230 7733; Kátine Marchezan Estivalet (55) 9 9936 9852. Or, you can contact the Human Research Ethics Committee of the Federal University of Health Sciences of Porto Alegre (51-33038804), located at Rua Sarmento Leite, 245, Porto Alegre-RS. The researcher's address is available: Rua Sarmento Leite, 245, Porto Alegre-RS, room 300B, phone: 51-33038876.

Porto Alegre, de de 202___.

________________________ ______________________________

Responsible Researcher Participante

DECLARATION OF RESPONSIBILITY OF RESEARCH PERFORMERS

I explained the objective, the risks and benefits and the nature of the research. I clarified all the doubts of the research participants. The participant understood and agreed to participate in the research**.**

SIGNATURE OF THE RESPONSIBLE TEACHER:_

____

Fernanda Cechetti

Docente UFCSPA

ANNEX 1 – STAGES OF PARKINSON'S DISEASE ACCORDING TO HOEHN AND YAHR

STAGES	DESCRIPTION
0	No signs of the disease.
1	Unilateral disease.
1,5	Unilateral and axial involvement.
2	Bilateral disease without balance deficit (recovers balance by taking three steps back or less).
2,5	Mild bilateral disease, with recovery in postural stabilization.
3	Mild to moderate bilateral disease; some postural instability; ability to live independently.
4	Severe disability, still able to walk or stand without assistance.
5	Confined to bed or wheelchair unless helped.

Fonte: Goetz e colaboradores (2004).

ANNEX 2 – MONTREAL COGNITIVE ASSESSMENT

MONTREAL COGNITIVE ASSESSMENT (MOCA)
Versão Experimental Brasileira

Nome:________ Data de nascimento: __/__/__
Escolaridade:________ Data de avaliação: __/__/__
Sexo:________ Idade:____

VISUOESPACIAL / EXECUTIVA — Pontos

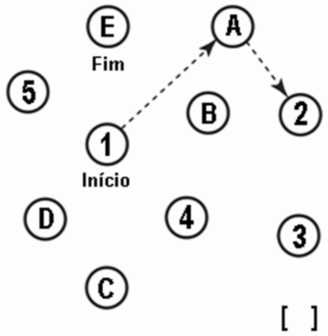

[ ]

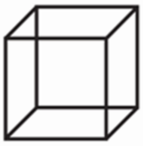

[ ]

Desenhar um RELÓGIO (onze horas e dez minutos) (3 pontos)

[ ] Contorno [ ] Números [ ] Ponteiros ___/5

NOMEAÇÃO

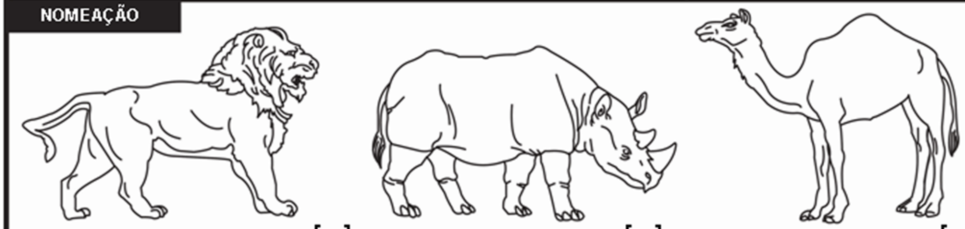

[ ] [ ] [ ] ___/3

MEMÓRIA

Leia a lista de palavras, O sujeito de repeti-la, faça duas tentativas Evocar após 5 minutos

	Rosto	Veludo	Igreja	Margarida	Vermelho
1ª tentativa					
2ª tentativa					

Sem Pontuação

ATENÇÃO

Leia a seqüência de números (1 número por segundo)
O sujeito deve repetir a seqüência em ordem direta [ ] 2 1 8 5 4
O sujeito deve repetir a seqüência em ordem indireta [ ] 7 4 2 ___/2

Leia a série de letras. O sujeito deve bater com a mão (na mesa) cada vez que ouvir a letra "A". Não se atribuem pontos se ≥ 2 erros.
[ ] F B A C M N A A J K L B A F A K D E A A A J A M O F A A B ___/1

Subtração de 7 começando pelo 100 [ ] 93 [ ] 86 [ ] 79 [ ] 72 [ ] 65
4 ou 5 subtrações corretas: 3 pontos; 2 ou 3 corretas 2 pontos; 1 correta 1 ponto; 0 correta 0 ponto ___/3

LINGUAGEM

Repetir: Eu somente sei que é João quem será ajudado hoje. [ ] O gato sempre se esconde embaixo do Sofá quando o cachorro está na sala. [ ] ___/2

Fluência verbal: dizer o maior número possível de palavras que comecem pela letra F (1 minuto). [ ]_____ (N ≥ 11 palavras) ___/1

ABSTRAÇÃO

Semelhança p. ex. entre banana e laranja = fruta [ ] trem - bicicleta [ ] relógio - régua ___/2

EVOCAÇÃO TARDIA

	Rosto	Veludo	Igreja	Margarida	Vermelho	
Deve recordar as palavras SEM PISTAS	[ ]	[ ]	[ ]	[ ]	[ ]	Pontuação apenas para evocação SEM PISTAS
OPCIONAL: Pista de categoria						
OPCIONAL: Pista de múltipla escolha						

___/5

ORIENTAÇÃO

[ ] Dia do mês [ ] Mês [ ] Ano [ ] Dia da semana [ ] Lugar [ ] Cidade ___/6

© Z. Nasreddine MD www.mocatest.org
Versão experimental Brasileira: *Ana Luisa Rosas Sarmento*
Paulo Henrique Ferreira Bertolucci - José Roberto Wajman
(UNIFESP-SP 2007)

TOTAL ___/30
Adicionar 1 pt se ≤ 12 anos de escolaridade

ANNEX 3 – KINESTHETIC AND VISUAL IMAGINATION QUESTIONNAIRE VISUAL

MOVIMENTOS	Flexão de Ombro	Oponência dos Dedos	Flexão anterior do Tronco	Abdução de Quadril	Bater os Pés
VISUAL					
5 = Imagem clara como visão (imagem tão nítida como vendo)					
4 = Imagem clara					
3 = Imagem moderadamente clara					
2 = Imagem borrada (embaçada)					
1 = Sem imagem					
CINESTÉSICA					
5 = Intensa como uma ação (tão intenso como executando a ação)					
4 = Intensa					
3 = Moderadamente intensa					
2 = Levemente intensa					
1 = Sem sensação					

Fonte: Malouin e colaboradores (2007).

ANNEX 4 - AVERAGE TIME TO COMPLETE THE TASK ACCORDING TO THE STAGES OF PARKINSON'S DISEASE

Hoehn e Yahr (estágios)	9-Hole Peg Test (em segundos)	
	mão dominante média ± DP	mão não dominante média ± DP
1	23.5 ± 5.6	23.5 ± 5.2
1.5	23.4 ± 3.2	31.2 ± 10.1
2	26.6 ± 6.6	27.5 ± 6.4
2.5	34.3 ± 22.5	34.4 ± 12.9
3	36.7 ± 16.4	36.8 ± 13.4

Fonte: Earhart e colaboradores (2011).

ANNEX 5 - UNIFIED PARKINSON'S DISEASE ASSESSMENT OF THE MOVEMENT DISORDERS SOCIETY

Parte III: Avaliação Motora

Visão Geral: avaliação dos sinais motores da DP.

Na parte superior do formulário, marque se o participante está utilizando medicação para o tratamento de sintomas da doença de Parkinson e, se estiver sob o uso de levodopa, o tempo desde a última dose.

Se o participante recebe tratamento para os sintomas da doença de Parkinson, marque também o estado clínico usando as seguintes definições:

ON é estado funcional típico de quando recebem medicação e têm uma boa resposta.

OFF é o estado funcional típico de quando os têm uma má resposta apesar de tomarem medicação.

Todos os itens devem ser pontuados com um valor inteiro (sem meios pontos, sem dados em falta). Instruções específicas são fornecidas para testar cada item. O avaliador demonstra enquanto descreve a tarefa que o participante deve realizar e pontua a função imediatamente depois. Para os itens Espontaneidade Global de Movimento e Tremor de Repouso, a pontuação será obtida durante toda a avaliação. No final da pontuação, indicar se discinesia (coreia ou distonia) esteve presente no momento da avaliação, e se assim for, se estes movimentos interferiram com a avaliação motora.

3a O participante usa medicação para o tratamento dos sintomas da doença de Parkinson?

Não (   )   Sim (   )

3b Se o participante recebe medicação para o tratamento dos sintomas da doença de Parkinson, marque o estado clínico do participante usando as seguintes definições:

ON: estado funcional típico de quando os participantes estão a tomar medicação e têm uma boa resposta.

OFF: estado funcional típico de quando os participantes têm uma resposta fraca apesar de tomarem medicação.

3c O participante usa Levodopa ?

Não (   )   Sim (   )

3.c1 Se sim, minutos desde a última dose de levodopa: _____________

RIGIDEZ

Instruções para o avaliador: A rigidez é avaliada usando movimentos passivos lentos das grandes articulações com o participante numa posição relaxada e o avaliador manipulando os membros. Primeiro teste sem a manobra de ativação. Teste e pontue cada membro separadamente. Para os braços, teste as articulações do punho e cotovelos simultaneamente. Se não for detectada rigidez, use uma manobra de ativação tais como bater o primeiro e o segundo dedo, abrir/fechar a mão. Explique ao participante que deve tentar relaxar o máximo possível enquanto é testada a rigidez.

0: Normal: Sem rigidez.

1: Discreto: Rigidez apenas detectada com uma manobra de ativação.

2: Ligeiro: Rigidez detectada sem a manobra de ativação, mas a amplitude total de movimento é facilmente alcançada.

3: Moderado: Rigidez detectada sem a manobra de ativação; amplitude total alcançada com esforço.

4: Grave: Rigidez detectada sem a manobra de ativação e amplitude total de movimento não alcançada.

(   ) MSE   (   ) MSD

BATER DOS DEDOS DA MÃO (PINÇA)

Instruções para o avaliador: Cada mão é testada separadamente. Faça a demonstração da tarefa, mas não realize a tarefa enquanto o participante é testado. Instrua o participante para que toque com o indicador no polegar 10 vezes, o mais rápido e amplo possível. Pontue cada lado separadamente, avaliando velocidade, amplitude, hesitações, interrupções e diminuição da amplitude.

0: Normal: Sem problemas.

1: Discreto: Qualquer dos seguintes: a) o ritmo regular é interrompido com uma ou duas interrupções ou hesitações nos movimentos; b) lentidão mínima; c) a amplitude diminui perto do fim das 10 repetições.

2: Ligeiro: Qualquer um dos seguintes: a) 3 a 5 interrupções durante os movimentos; b) lentidão ligeira; c) a amplitude diminui no meio da sequência das 10 repetições

3: Moderado: Qualquer um dos seguintes: a) mais de 5 interrupções durante os movimentos ou pelo menos uma pausa mais longa (*bloqueio*); b) lentidão moderada; c) a amplitude diminui após o primeiro movimento.

4: Grave: Não consegue ou quase não consegue executar a tarefa devido à lentidão, interrupções ou decrementos.

(   ) MSE   (   ) MSD

MOVIMENTOS DAS MÃOS

Instruções para o avaliador: Cada mão é testada separadamente. Faça a demonstração da tarefa, mas não realize a tarefa enquanto o participante é testado. Instrua o participante a fechar a mão com força com o braço fletido ao nível do cotovelo de forma que a palma da mão esteja virada para o avaliador. Peça ao participante para abrir a mão 10 vezes o mais rápido e amplo possível. Se o participante não fechar a mão firmemente ou não abrir a mão por completo, lembre-o de o fazer. Pontue cada lado separadamente, avaliando velocidade, amplitude, hesitações, interrupções e diminuições da amplitude.

0: Normal: Sem problemas.

1: Discreto: Qualquer dos seguintes: a) o ritmo regular é interrompido com uma ou duas interrupções ou hesitações dos movimentos; b) lentidão mínima; c) a amplitude diminui perto do fim da tarefa.

2: Ligeiro: Qualquer dos seguintes: a) 3 a 5 interrupções durante o movimento; b) lentidão ligeira; c) a amplitude diminui no meio da tarefa.

3: Moderado: Qualquer dos seguintes: a) mais de 5 interrupções durante o movimento ou pelo menos uma pausa mais prolongada (*bloqueio*); b) lentidão moderada; c) a amplitude diminui após a primeira sequência de abrir e fechar.

4: Grave: Não consegue ou quase não consegue executar a tarefa devido à lentidão, interrupções ou decrementos.

(   ) MSE   (   ) MSD

MOVIMENTOS DE PRONAÇÃO-SUPINAÇÃO DAS MÃOS

Instruções para o avaliador: Cada mão é testada separadamente. Faça a demonstração da tarefa, mas não realize a tarefa enquanto o participante é testado. Instrua o participante a estender o braço em frente ao seu corpo com a palma da mão virada para baixo; depois a virar a palma da mão para cima e para baixo alternadamente 10 vezes o mais rápido e amplo possível. Pontue cada lado separadamente, avaliando velocidade, amplitude, hesitações, interrupções e diminuições da amplitude.

0: Normal: Sem problemas.

1: Discreto: Qualquer dos seguintes: a) o ritmo regular é interrompido com uma ou duas interrupções ou hesitações dos movimentos; b) lentidão mínima; c) a amplitude diminui perto do fim da sequência.
2: Ligeiro: Qualquer dos seguintes: a) 3 a 5 interrupções durante o movimento; b) lentidão ligeira; c) a amplitude diminui no meio da sequência.
3: Moderado: Qualquer dos seguintes: a) mais de 5 interrupções durante o movimento ou pelo menos uma pausa mais prolongada (*bloqueio*); b) lentidão moderada; c) a amplitude diminui após a primeira sequência de pronação-supinação.
4: Grave: Não consegue ou quase não consegue executar a tarefa devido à lentidão, interrupções ou decrementos.
( ) MSE ( ) MSD

TREMOR POSTURAL DAS MÃOS

Instruções para o avaliador: Todo o tremor, incluindo o tremor de repouso reemergente, que está presente na postura é incluído nesta pontuação. Pontue cada mão separadamente. Pontue a maior amplitude observada. Instrua o participante a estender os braços em frente do corpo com as palmas das mãos viradas para baixo. O punho deve estar reto e os dedos confortavelmente separados para que não se toquem. Observe esta postura durante 10 segundos.
0: Normal: Sem tremor.
1: Discreto: O tremor está presente mas tem menos de 1 cm de amplitude.
2: Ligeiro: O tremor tem pelo menos 1 cm mas menos de 3 cm de amplitude.
3: Moderado: O tremor tem pelo menos 3 cm, mas menos de 10 cm de amplitude.
4: Grave: O tremor tem pelo menos 10 cm de amplitude.
( ) MSE ( ) MSD

TREMOR CINÉTICO DAS MÃOS

Instruções para o avaliador: Este tremor é testado através da manobra de dedo-nariz. Iniciando com o braço estendido, peça ao participante que execute pelo menos três manobras dedo nariz com cada mão, chegando o mais longe possível para tocar o dedo do avaliador. A manobra dedo-ao-nariz deve ser executada com lentidão suficiente para que o tremor não seja ocultado o que pode acontecer com movimentos muito rápidos do braço. Repetir com a outra mão, pontuando cada mão separadamente. O tremor pode estar

presente durante o movimento ou quando se alcança qualquer um dos alvos (nariz ou dedo). Pontue a maior amplitude observada.

0: Normal: Sem tremor.

1: Discreto: O tremor está presente mas tem menos de 1 cm de amplitude.

2: Ligeiro: O tremor tem pelo menos 1 cm mas menos de 3 cm de amplitude.

3: Moderado: O tremor tem pelo menos 3 cm mas menos de 10 cm de amplitude.

4: Grave: O tremor tem pelo pelo menos 10 cm de amplitude.

(   ) MSE   (   ) MSD

AMPLITUDE DO TREMOR DE REPOUSO

Instruções para o avaliador: Este e o próximo item foram colocados deliberadamente no final da avaliação para permitir ao avaliador reunir observações sobre o tremor de repouso que podem ter surgido a qualquer momento da avaliação, incluindo quando o participante está calmamente sentado, durante a marcha e durante as atividades em que algumas partes do corpo estão em movimento, mas outras estão em repouso. Pontue a amplitude máxima observada em qualquer momento, como a pontuação final. Pontue apenas a amplitude e não a persistência ou a intermitência do tremor. Como parte desta pontuação, o participante deve sentar-se calmamente numa cadeira, com as mãos colocadas nos braços da cadeira (e não no colo) e os pés confortavelmente apoiados no chão durante 10 segundos sem nenhuma outra instrução. O tremor de repouso é avaliado separadamente, mas será considerado para os membros superiores. Pontue apenas a amplitude máxima observada a qualquer momento, sendo essa a pontuação final.

Extremidades

0: Normal: Sem tremor.

1: Discreto.: < 1 cm de amplitude máxima.

2: Ligeiro: = 1 cm mas < 3 cm de amplitude máxima.

3: Moderado: = 3 cm mas < 10 cm de amplitude máxima.

4: Grave: = 10 cm de amplitude máxima.

(   ) MSE   (   ) MSD

PERSISTÊNCIA DO TREMOR DE REPOUSO

Instruções para o avaliador: Este item recebe uma pontuação única para todo o tremor de repouso foca-se na persistência do tremor de repouso durante o período de avaliação quando diferentes partes do corpo estão em repouso. Este item é pontuado

deliberadamente no final da avaliação para que vários minutos de informação possam ser reunidos em uma única pontuação.

0: Normal: Sem tremor.

1: Discreto: Tremor de repouso presente durante = 25% do tempo de avaliação.

2: Ligeiro: Tremor de repouso presente durante 26-50% do tempo de avaliação.

3: Moderado: Tremor de repouso presente durante 51-75% do tempo de avaliação.

4: Grave: Tremor de repouso presente durante > 75% do tempo de avaliação.

( ) MSE ( ) MSD

IMPACTO DAS DISCINESIAS NAS PONTUAÇÕES DA PARTE III

A. Estiveram presentes discinesias (coreia ou distonia) durante a avaliação?

Não ( ) Sim ( )

B. Se sim, estes movimentos interferiram com as suas pontuações?

Não ( ) Sim ( )

ANNEX 6 - TEST D’ÉVALUATION OF SENIOR MEMBERS OF PERSONNES AGÉES

TEMPA – versão brasileira

**Nome:**			**Idade:**
**Diagnóstico:**	**Dominância:**	**Data:**	**Amplitude de movimento Passiva:**

**Alteração Visual:** ( ) Sim ( ) Não	**Uso de óculos:** ( ) Sim ( ) Não
**Prejuízo perceptual ou cognitivo:** ( ) Sim ( ) Não	Não avaliado ( )

	Análise das Tarefas														
	Velocidade de execução		Graduação Funcional		Amplitude ativa de movimento		Força		Precisão movimentos amplos		Preensão		Precisão movimentos finos		Observações / Comentários
Tarefas	D	E	D	E	D	E	D	E	D	E	D	E	D	E	
1. Pegar e transportar um pote													xxxx	xxxxx	
2. Abrir um pote, tirar uma colher cheia de café															
3. Pegar uma jarra e servir água em um copo													xxxxxxxxxxxxx		
4. Destrancar fechadura e abrir um recipiente contendo pílulas															
5. Escrever em um envelope e colar um selo							xxxxxxxxx								
6. Embaralhar e distribuir cartas de jogo							xxxxxxxxx								
7. Manusear moedas							xxxxxxxxx								
8.Pegar e mover pequenos objetos							xxxxxxxxx								
															Análise Total das Tarefas
Escore Total Tarefas Unilaterais															
Escore Total Tarefas Bilaterais															
Escore Total Combinado															

	Direito	Esquerdo
**Força de Preensão (Kg)**		
**Resistência (seg)**		

ANNEX 7 – PARKINSON'S DISEASE-COGNITIVE RATING SCALE

1. MEMÓRIA VERBAL COM EVOCAÇÃO LIVRE IMEDIATA

INSTRUÇÕES

Leia para o sujeito, em voz alta, as palavras na lista abaixo (ritmo de uma palavra por segundo). Três tentativas são realizadas, e pede-se ao sujeito que se lembre do maior número possível de palavras após cada tentativa. Leia para o sujeito: **"Eu vou ler uma lista de palavras e, quando finalizar, quero que você repita o maior número de palavras que puder se recordar. Não precisa ser na mesma ordem da minha leitura. Alguma dúvida? Podemos começar?".** (Leia a primeira lista, no ritmo de uma palavra por segundo. Ao concluir, aguarde as respostas do sujeito. Ao terminar, continue com a instrução da 2a tentativa).

	PONTOS			PONTOS	
LUZ	0	1	QUADRO	0	1
SEDA	0	1	BICICLETA	0	1
AREIA	0	1	ESTRELA	0	1
CÍLIO	0	1	LEÃO	0	1
ARROZ	0	1	ANEL	0	1
GRAVATA	0	1	PERFUME	0	1

PONTUAÇÃO (1a. TENTATIVA): ____/12

"Agora vou ler as mesmas palavras e quero que se lembre da maior quantidade que puder se lembrar, inclusive as palavras que já foram ditas na primeira tentativa. Não importa a ordem das palavras, procure dizer todas elas incluindo aquelas que já foram recordadas na vez anterior. Pronto?". (Leia as palavras da 2a tentativa, no ritmo de uma palavra por segundo e aguarde as respostas do sujeito).

	PONTOS			PONTOS	
LUZ	0	1	QUADRO	0	1
SEDA	0	1	BICICLETA	0	1
AREIA	0	1	ESTRELA	0	1
CÍLIO	0	1	LEÃO	0	1
ARROZ	0	1	ANEL	0	1
GRAVATA	0	1	PERFUME	0	1

PONTUAÇÃO (2a. TENTATIVA): ____/12

(continuação)

"Agora, vou ler as mesmas palavras pela terceira vez e quero que se lembre da maior quantidade que puder se lembrar, inclusive as palavras que já foram ditas nas tentativas anteriores. Não importa a ordem das palavras, procure dizer todas elas incluindo aquelas que já foram recordadas na vez anterior. Pronto?".

	PONTOS			PONTOS	
LUZ	0	1	QUADRO	0	1
SEDA	0	1	BICICLETA	0	1
AREIA	0	1	ESTRELA	0	1
CÍLIO	0	1	LEÃO	0	1
ARROZ	0	1	ANEL	0	1
GRAVATA	0	1	PERFUME	0	1

PONTUAÇÃO (3a. TENTATIVA): _____/12

MAIOR PONTUAÇÃO ENTRE AS TRÊS TENTATIVAS: _____/12

2. NOMEAÇÃO POR CONFRONTO VISUAL

INSTRUÇÕES

Pede-se ao sujeito que nomeie os desenhos mostrados nos 20 cartões consecutivos. Não há tempo limite para a resposta, e apenas uma única tentativa é permitida. Nenhuma pista semântica ou fonêmica é oferecida. Quando os objetos são incluídos dentro de seu contexto (babador, fivela, crina, anzol, sino e casco), o avaliador pode indicar a parte do desenho a ser nomeado. Qualquer uma das palavras (sinônimas) descritas no quadro podem ser pontuadas.

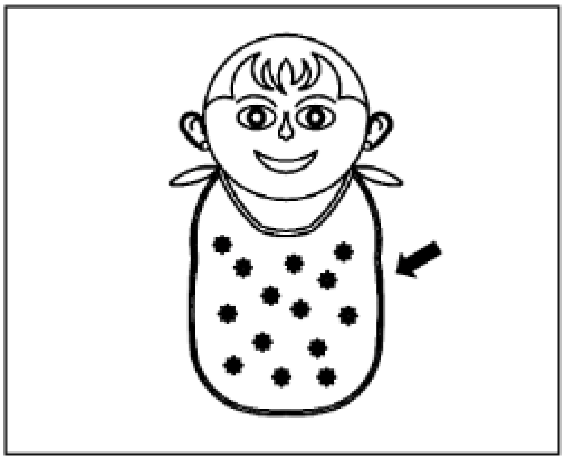

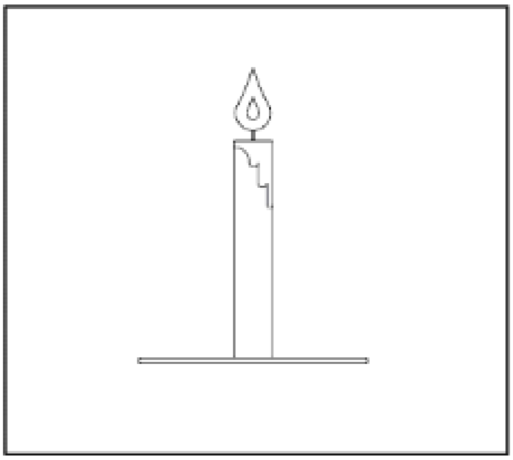

(continuação)

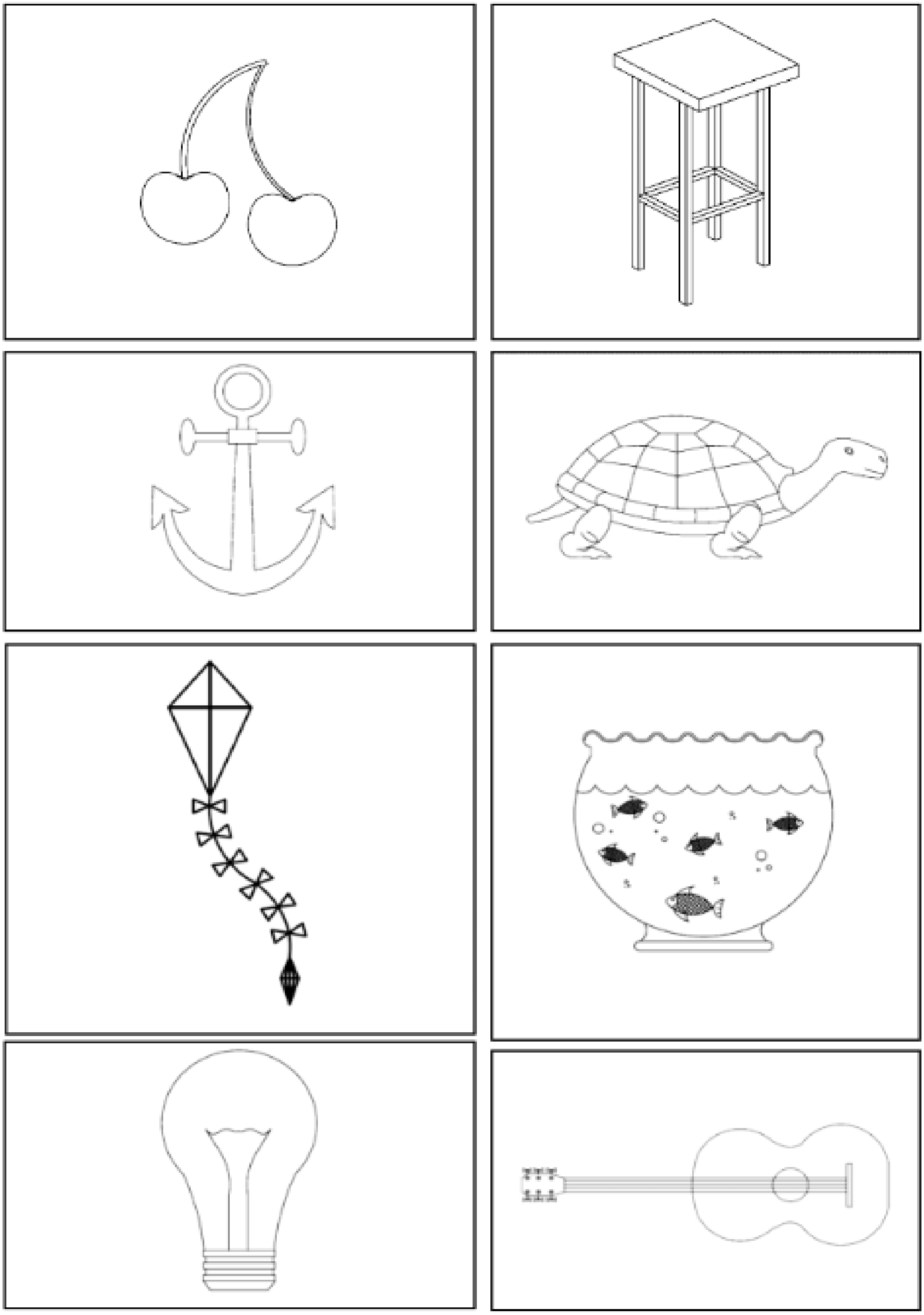

(continuação)

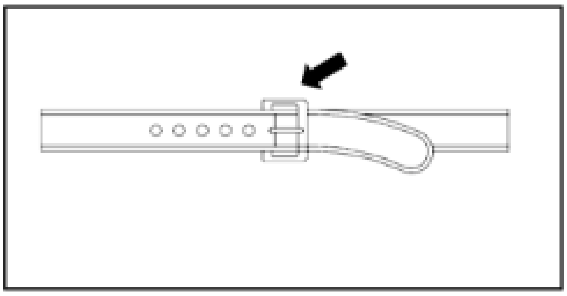
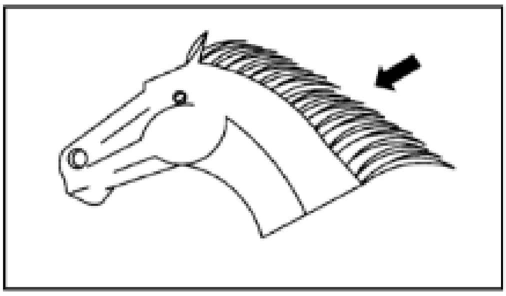
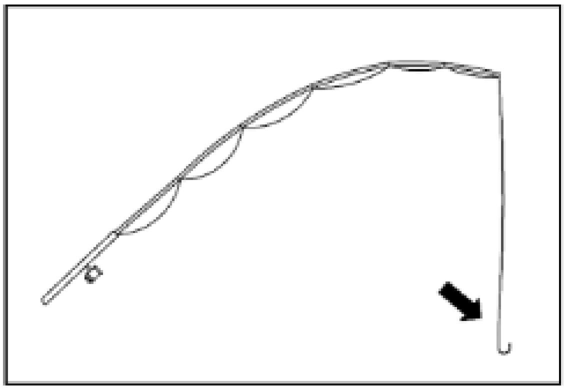
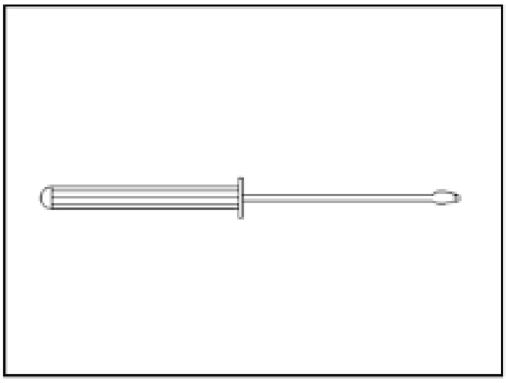
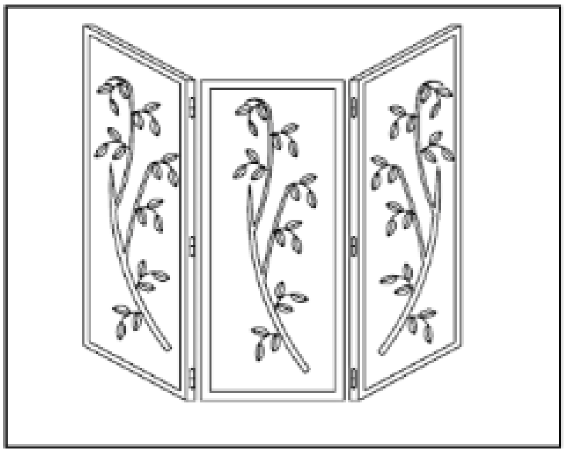
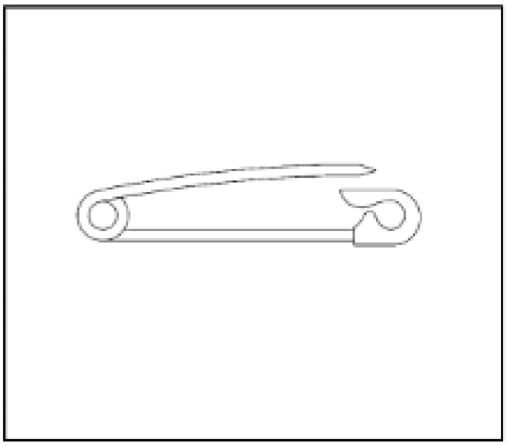
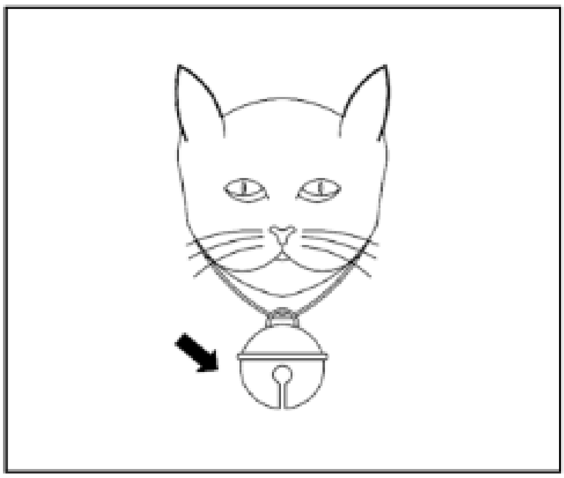
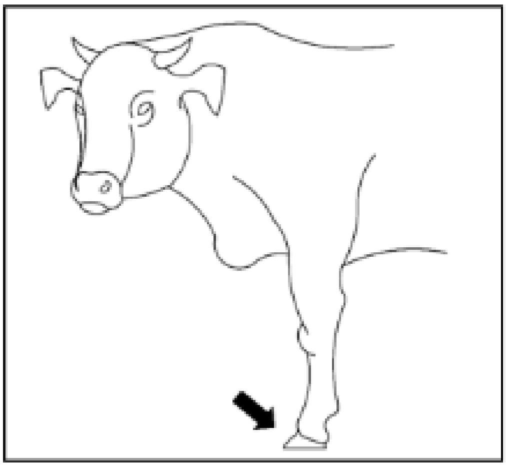

(continuação)

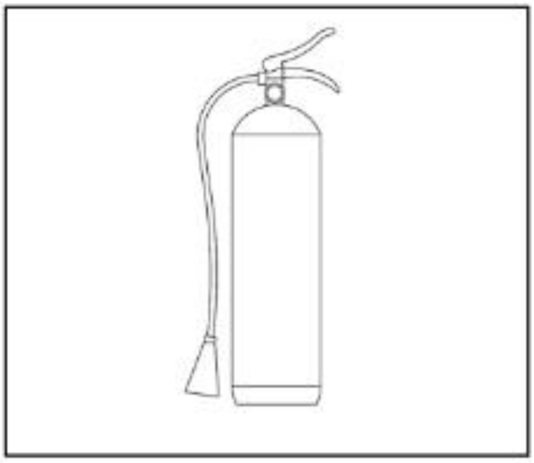

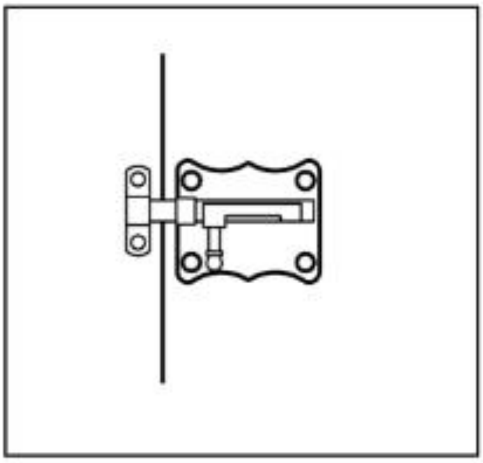

	PONTOS	
BABADOR	0	1
VELA	0	1
CEREJAS / MAÇÃS	0	1
BANQUETA / BANQUINHO / TAMBORETE / BANCO	0	1
ÂNCORA	0	1
TARTARUGA / JABOTI / CÁGADO / TRACAJÁ	0	1
PIPA / PAPAGAIO / ARRAIA	0	1
AQUÁRIO	0	1
LÂMPADA	0	1
VIOLÃO / VIOLA / GUITARRA	0	1
FIVELA	0	1
CRINA / JUBA	0	1
ANZOL	0	1
CHAVE DE FENDA	0	1
ANTEPARO / DIVISÓRIA / BIOMBO	0	1
ALFINETE	0	1
SINO / SININHO / GUIZO / MEDALHA DE IDENTIFICAÇÃO	0	1
CASCO / PATA	0	1
EXTINTOR	0	1
FECHADURA / TRINCO / FERROLHO	0	1

PONTUAÇÃO TOTAL: ____/20

(continuação)

3. ATENÇÃO SUSTENTADA

INSTRUÇÕES

Uma série ascendente de letras e números é lida para o sujeito. Pede-se que sujeito relate a quantidade de letras na sequência lida. Dez séries de letras e números são apresentadas, divididas em cinco níveis de complexidade crescente. Duas séries serão usadas, no início do teste, como treino. Diga ao sujeito: **"Eu vou ler uma sequência de números e letras. Quando eu acabar, quero que me diga quantas letras você ouviu. Preste bastante atenção, pois não posso repetir. Por exemplo, vou dizer 2 - L - T. Quantas letras tem?** (o sujeito responde 2 letras). **Muito bem! Se eu disser 8 - A - 9, quantas letras tem?** (1 letra). **Ok! Vamos começar?"**.

TREINO (EXEMPLO)

		RESPOSTA CORRETA
2 L T	→	2 LETRAS
8 A 9	→	1 LETRA

TESTE

	RESPOSTA CORRETA	PONTOS
2 P 6 5 4	1 LETRA	0 1
3 A 6 K L	3 LETRAS	0 1
B 9 0 4 L T	3 LETRAS	0 1
3 C P 5 7 3	2 LETRAS	0 1
3 9 5 L 4 Z A	3 LETRAS	0 1
I 1 A S Q 4 1	4 LETRAS	0 1
7 5 D A 4 T B 2	4 LETRAS	0 1
9 6 8 4 3 7 L C	2 LETRAS	0 1
Z 4 9 A T D 3 8 4	4 LETRAS	0 1
9 5 M D 4 S C 3 E	5 LETRAS	0 1

PONTUAÇÃO TOTAL: ____/10

(continuação)

4. MEMÓRIA OPERACIONAL

INSTRUÇÕES

O examinador lê em voz alta uma lista randomizada de números e letras, variando em extensão de 2 a 6 letras e números. Após cada série, pede-se que o sujeito repita os números, em primeiro lugar e, então, as letras. Este teste termina quando o sujeito não for mais capaz de dar a resposta correta em duas séries consecutivas. Duas séries serão apresentadas, no início do teste, como treino. Diga ao sujeito: **"Vou ler uma lista de números e letras. Quando terminar, quero que você repita primeiro os números e depois as letras. Por exemplo, vou dizer L - 2 - T e o que você responde?** (o sujeito diz: 2 - L - T). **Muito bem! Mais uma vez: 8 - A - 9.** (o sujeito diz: 8 - 9 - A). **Muito bem! Podemos começar?"**

TREINO (EXEMPLO)

	RESPOSTA CORRETA
L 2 T →	2 L T
8 A 9 →	8 9 A

TESTE

	RESPOSTA CORRETA	PONTOS
M 3	3 M	0 1
7 P	7 P	0 1
G 8 M	8 G M	0 1
9 I 6	9 6 I	0 1
T 0 4 A	0 4 T A	0 1
7 V 6 J	7 6 V J	0 1
M 6 4 N I	6 4 M N I	0 1
3 5 S G C	3 5 S G C	0 1
1 R 9 V B 3	1 9 3 R V B	0 1
M 2 7 4 Z 9	2 7 4 9 M Z	0 1

PONTUAÇÃO TOTAL: ____/10

(continuação)

5.DESENHO DO RELÓGIO (ESPONTÂNEO)

INSTRUÇÕES

Pede-se que sujeito desenhe um relógio numa folha de papel em branco e colocar os ponteiros marcando "dez horas e vinte e cinco minutos" (0 a 10). Diga ao sujeito: "**Quero que você desenhe um relógio com todos os números, com os ponteiros marcando dez horas e vinte e cinco minutos. O relógio deve ter um contorno e os dois ponteiros devem ser desenhados na forma de setas. Pode começar.**".

(continuação)

5.DESENHO DO RELÓGIO (ESPONTÂNEO)

INSTRUÇÕES

Pede-se que sujeito desenhe um relógio numa folha de papel em branco e colocar os ponteiros marcando "dez horas e vinte e cinco minutos" (0 a 10). Diga ao sujeito: "**Quero que você desenhe um relógio com todos os números, com os ponteiros marcando dez horas e vinte e cinco minutos. O relógio deve ter um contorno e os dois ponteiros devem ser desenhados na forma de setas. Pode começar.**".

(continuação)

DESENHO ESPONTÂNEO DO RELÓGIO	PONTOS	
A figura parece um relógio.	0	1
O relógio não é dividido por linhas ou setores.	0	1
Há uma disposição simétrica dos números.	0	1
Somente são escritos números no intervalo de 1 a 12.	0	1
Os números das horas estão corretamente sequenciados.	0	1
Somente dois ponteiros são desenhados.	0	1
Os ponteiros são representados como setas.	0	1
O ponteiro das horas é menor que o ponteiro dos minutos.	0	1
Nenhuma palavra foi escrita.	0	1
O número "25" não foi desenhado.	0	1

PONTUAÇÃO TOTAL: _____/10

CÓPIA DO DESENHO DO RELÓGIO	PONTOS	
A figura parece um relógio.	0	1
O relógio não é dividido por linhas ou setores.	0	1
Há uma disposição simétrica dos números.	0	1
Somente são escritos números no intervalo de 1 a 12.	0	1
Os números das horas estão corretamente sequenciados.	0	1
Somente dois ponteiros são desenhados.	0	1
Os ponteiros são representados como setas.	0	1
O ponteiro das horas é menor que o ponteiro dos minutos.	0	1
Nenhuma palavra foi escrita.	0	1
O número "25" não foi desenhado.	0	1

PONTUAÇÃO TOTAL: _____/10

(continuação)

7. MEMÓRIA VERBAL DE EVOCAÇÃO LIVRE TARDIA

INSTRUÇÕES

Pede-se ao sujeito para se lembrar do maior número de palavras que puder da lista de palavras apresentadas no início do teste. Diga ao sujeito: **"Quero que você tente se lembrar daquela lista de palavras que foi lida três vezes para você no início deste teste. Tente se lembrar da maior quantidade de palavras que puder, em qualquer ordem".** Não forneça pistas aos pacientes.

	PONTOS			PONTOS	
LUZ	0	1	QUADRO	0	1
SEDA	0	1	BICICLETA	0	1
AREIA	0	1	ESTRELA	0	1
CÍLIO	0	1	LEÃO	0	1
ARROZ	0	1	ANEL	0	1
GRAVATA	0	1	PERFUME	0	1

PONTUAÇÃO: ____/12

7. FLUÊNCIA VERBAL ALTERNADA

INSTRUÇÕES

Pede-se ao sujeito para gerar o maior número de palavras possíveis que comecem com a letra 'S' de maneira alternada com a maior quantidade possível de "itens do vestuário" durante 60 segundos. Os participantes são instruídos a não usarem nomes próprios ou repetir a mesma palavra com terminação diferente (ex.: sapo, sapa, sapinho). Diga ao sujeito: **"Durante um minuto, quero que me diga a maior quantidade de palavras que comecem com a letra 'S', de maneira alternada com a maior quantidade possível de itens do vestuário. Você irá dizer algo que comece com a letra 'S' e em seguida uma vestimenta. Depois outra palavra com 'S' e outra vestimenta. Lembre-se que as vestimentas podem começar com qualquer letra. Não use nomes próprios. Fará isto o mais rapidamente que puder por um minuto. Podemos começar?".** (Marque 60 segundos no relógio).

REGRA DE PONTUAÇÃO:

1 ponto para cada resposta correta que mantenha a alternância entre as palavras iniciadas com 'S' e um "item do vestuário" (0 a 20).

(continuação)

PALAVRAS INICIADAS POR "S"	VESTIMENTA

PONTUAÇÃO: _____/20

7. FLUÊNCIA VERBAL DE AÇÕES

INSTRUÇÕES

Leia para o sujeito: **"Durante 60 segundos, quero que me diga o maior número de coisas diferentes que você puder pensar que as pessoas fazem. Eu não quero que você use a mesma palavra com diferentes terminações, como "comer, comendo, comido". Também, me diga apenas palavras como "comer", ou "cheirar", no lugar de uma frase ou sentença (ex. indo comer). Quero somente as palavras, entendeu? Podemos começar?".**

REGRA DE PONTUAÇÃO:

1 ponto para cada resposta correta (0 a 30).

PALAVRAS QUE REPRESENTAM "AÇÕES" ("COISAS QUE AS PESSOAS FAZEM")

PONTUAÇÃO: _____/30

(continuação)

TABELA DE CORREÇÃO DOS TESTES	PONTUAÇÃO
MEMÓRIA VERBAL IMEDIATA	____/12
NOMEAÇÃO POR CONFRONTO VISUAL	____/20
ATENÇÃO SUSTENTADA	____/10
MEMÓRIA OPERACIONAL	____/10
DESENHO DO RELÓGIO	____/10
CÓPIA DO RELÓGIO	____/10
MEMÓRIA VERBAL TARDIA	____/12
FLUÊNCIA VERBAL ALTERNADA	____/20
FLUÊNCIA VERBAL DE AÇÕES	____/30
**FRONTAL-SUBCORTICAL**	____/104
**CORTICAL POSTERIOR**	____/30
**TOTAL PD-CRS**	____/134

Observação:
O escore **Cortical Posterior** é composto pela soma dos itens sublinhados.
O escore F**rontal-Subcortical** é composto pela soma dos demais itens.

ANEXO 8 - MEDIDA CANADENSE DE TERAPIA OCUPACIONAL

MEDIDA CANADENSE DE DESEMPENHO OCUPACIONAL (COPM)[1]

Segunda Edição

Autores: Mary Law, Sue Baptiste, Anne Carswell, Mary Ann McColl, Helene Polatajko, Nancy Pollock[2]

Nome do cliente: ______________________ Idade: ________ Sexo: ________

Entrevistado: ______________________ Registro nº: ________

(se não for o cliente)

Data da avaliação: ________

Terapeuta: ______________________

Data prevista para reavaliação: ________

Clínica/Hospital: ____________ Programa: ____________

Data da reavaliação: ________

PASSO 1: IDENTIFICAÇÃO DE QUESTÕES NO DESEMPENHO OCUPACIONAL

Para identificar problemas, preocupações e questões relativas ao desempenho ocupacional, entreviste o cliente questionando sobre as atividades do dia-a-dia no que se refere às atividades produtivas, de autocuidado e de lazer. Solicite ao cliente que identifique as atividades do dia-a-dia que quer realizar, que necessita realizar ou que é esperado que ele realize, encorajando-o a pensar num dia típico. Em seguida, peça que identifique quais dessas atividades atualmente são difíceis de realizar, de forma satisfatória. Registre estas atividades problemáticas nos Passos 1A, 1B ou 1C.

PASSO 2: CLASSIFICAÇÃO DO GRAU DE IMPORTÂNCIA

Usando os cartões de pontuação, peça ao cliente que classifique, numa escala de 1 a 10, a importância de cada atividade. Coloque as pontuações nos respectivos quadrados nos Passos 1A, 1B e 1C.

A. Autocuidado		Importância
**Cuidados pessoais**	______	☐
(ex.: vestuário, banho,	______	☐
alimentação, higiene)	______	☐
**Mobilidade funcional:**	______	☐
(ex.: transferências, mobilidade	______	☐
dentro e fora de casa)	______	☐
**Independência fora de casa:**	______	☐
(ex.: transportes, compras, finanças)	______	☐
	______	☐

B. Produtividade		Importância
**Trabalho (remunerado/não-remunerado)**	______	☐
(ex.: procurar/manter um emprego,	______	☐
atividades voluntárias)	______	☐
**Tarefas domésticas**	______	☐
(ex.: limpezas, lavagem de roupas,	______	☐
preparação de refeições)	______	☐
**Brincar/Escola**	______	☐
(ex.: habilidade para brincar,	______	☐
fazer o dever de casa)	______	☐

C. Lazer		Importância
**Recreação tranqüila**	______	☐
(ex.: *hobbies*, leitura, artesanato)	______	☐
	______	☐
**Recreação ativa**	______	☐
(ex.: esportes, passeios, viagens)	______	☐
	______	☐
**Socialização**	______	☐
(ex.: visitas, telefonemas,	______	☐
festas, escrever cartas)	______	☐

[1] *Canadian Occupational Performance Measure* (COPM). Versão brasileira traduzida por Lívia C. Magalhães, Lilian V. Magalhães e Ana Amélia Cardoso.

[2] Publicado pela CAOT Publications ACE © M. Law, S. Baptiste, A. Carswell, M. A. McColl, H. Polatajko, N. Pollock, 2000

(continuação)

PASSO 3: PONTUAÇÃO – AVALIAÇÃO INICIAL

Confirme com o cliente os 5 problemas mais importantes e registre-os abaixo. Usando os cartões de pontuação, peça ao cliente para classificar cada problema no que diz respeito ao Desempenho e Satisfação, depois calcule a pontuação total. Para calcular a pontuação total some a pontuação do desempenho ocupacional ou da satisfação de todos os problemas e divida pelo número de problemas.

PASSO 4: REAVALIAÇÃO

No intervalo de tempo apropriado para reavaliação, o cliente classifica novamente cada problema, no que se refere ao Desempenho e à Satisfação.

Problemas de Desempenho Ocupacional	Avaliação Inicial		Reavaliação	
	Desempenho 1	Satisfação 1	Desempenho 2	Satisfação 2
1.				
2.				
3.				
4.				
5.				
Problemas de Desempenho Ocupacional	Pontuação do Desempenho 1	Pontuação da Satisfação 1	Pontuação do Desempenho 2	Pontuação da Satisfação 2
Pontuação Total = Pontuação Total do Desempenho ou da Satisfação / Nº de Problemas	___ / ___ = ___	___ / ___ = ___	___ / ___ = ___	___ / ___ = ___

PASSO 5: COMPUTANDO OS ESCORES DE MUDANÇA

Calcule as mudanças, subtraindo a pontuação obtida na avaliação da obtida na reavaliação.

Mudança no Desempenho = Pontuação do Desempenho 2 ___ – Pontuação do Desempenho 1 ___ = ___

Mudança na Satisfação = Pontuação da Satisfação 2 ___ – Pontuação da Satisfação 1 ___ = ___

ANOTAÇÕES ADICIONAIS E OBSERVAÇÕES

Avaliação inicial:

Reavaliação:

[1] *Canadian Occupational Performance Measure* (COPM). Versão brasileira traduzida por Lívia C. Magalhães, Lilian V. Magalhães e Ana Amélia Cardoso.

[2] Publicado pela CAOT Publications ACE © M. Law, S. Baptiste, A. Carswell, M. A. McColl, H. Polatajko, N. Pollock, 2000
