# Supplementary material for: The use of brain-machine interface, motor imagery, and action observation in the rehabilitation of individuals with Parkinson’s disease: A protocol study for a randomized clinical trial
Source: PLoS One. 2025 Apr 7;20(4):e0315148. doi: 10.1371/journal.pone.0315148 (PMC11975075; doi:10.1371/journal.pone.0315148)
Supplement: S5 File — (PDF) [file pone.0315148.s010.pdf]

UNIVERSIDADE FEDERAL DE CIÊNCIAS DA SAÚDE DE PORTO ALEGRE
PÓS-GRADUAÇÃO EM CIÊNCIAS DA REABILITAÇÃO

Kátine Marchezan Estivalet

EFEITOS DA IMAGINAÇÃO MOTORA E DA OBSERVAÇÃO DA AÇÃO NAS ALTERAÇÕES MOTORAS EM MEMBROS SUPERIORES E NAS ALTERAÇÕES COGNITIVAS NA DOENÇA DE PARKINSON: ENSAIO CLÍNICO RANDOMIZADO

Equipe de Pesquisa: Fisioterapeutas Rafael Goldani e Tatiana Salayaran de Aguiar Pettenuzzo

Porto Alegre
2022

Kátine Marchezan Estivalet

EFEITOS DA IMAGINAÇÃO MOTORA E DA OBSERVAÇÃO DA AÇÃO NAS ALTERAÇÕES MOTORAS EM MEMBROS SUPERIORES E NAS ALTERAÇÕES COGNITIVAS NA DOENÇA DE PARKINSON: ENSAIO CLÍNICO RANDOMIZADO

Projeto de Pesquisa de Doutorado submetido ao Programa de Pós-Graduação em Ciências da Reabilitação da Universidade Federal de Ciências da Saúde de Porto Alegre, para qualificação de tese.

Orientadora: Profa. Dra. Fernanda Cechetti

Porto Alegre

2022

RESUMO

A doença de Parkinson é degenerativa, progressiva e crônica. Considerada potencialmente incapacitante, em vista das alterações motoras, como bradicinesia, rigidez e tremor nos membros superiores, e alterações não-motoras, como as cognitivas envolvendo dificuldades de atenção e concentração e de memória. Assim, tem-se apostado nas modalidades de neuroreabilitação, como a imaginação motora e a observação da ação. O objetivo da pesquisa é investigar os efeitos da imaginação motora e da observação da ação nas alterações motoras em membros superiores e nas alterações cognitivas na doença de Parkinson. Trata-se de um estudo do tipo ensaio clínico controlado randomizado. A população do estudo envolve pessoas com doença de Parkinson no estágio 1-3 na escala Hoehn e Yahr, na faixa etária entre 20 até 59 anos, e deve estar fazer uso estável de medicamentos, não apresentar alteração cognitiva com risco de demência, e ser capaz de imaginar atividades motoras, e apresentar alteração motora em membro superior. Os grupos do estudo serão: a) imaginação motora, observação da ação e execução motora; b) imaginação motora e execução motora; c) observação da ação e execução motora; d) imaginação motora e execução motora e exoesqueleto; e) observação da ação e execução motora e exoesqueleto. As intervenções de todos grupos serão de uma abordagem intensivista de 10 sessões contínuas, com intervalo de dois dias na metade da intervenção, totalizando duas semanas, sendo cada sessão de 60 minutos por dia. As etapas para coleta de dados do estudo envolverão o pré-teste, as intervenções, o pós-teste imediato e o teste após um período de quatro semanas sem intervenção. Os instrumentos que serão usados para as avaliações: a) parte da Escala de Avaliação da Doença de Parkinson Unificada (UPDRS-III); b) *Test D'évaluation Des Membres Supérieurs Des Personnes Âgées* (TEMPA); c) *9-Hole Peg Test* para avaliar a função da extremidade superior; d) Escala de Avaliação Cognitiva da Doença de Parkinson; e) Medida Canadense de Desempenho Ocupacional para identificar o desempenho e a satisfação na realização das atividades-problema que considera importante nas áreas de autocuidado, produtividade e lazer.

Palavras-chave: Cognição. Doença de Parkinson. Imaginação. Reabilitação.

LISTA DE ABREVIATURAS E SIGLAS

9HPT	9-Hole Peg Test
BCI	*Brain Computer Interface*
CEP	Comitê de Ética em Pesquisa
COPM	Medida Canadense de Desempenho Ocupacional
DP	Doença de Parkinson
EA	Execução Da Ação
EEG	Eletroencefalografia
ICM	Interface Cérebro-Máquina
IM	Imaginação Motora
fNIRS	Espectroscopia Funcional de Infra-vermelho
GE	Grupos Experimentais
GR	Grupo Referência
KVIQ-10	Questionário de Imagens Cinestésicas e Visuais
MDS-UPDRS	Escala de Avaliação da Doença de Parkinson Unificada da Sociedade de Distúrbios do Movimento
MEG	Magnetoencefalografia
MoCA	*Montreal Cognitive Assessment*
OA	Observação De Ação
PD-CRS	Escala de Avaliação Cognitiva da Doença de Parkinson
RS	Rio Grande do Sul
TEMPA	*Test d'Évaluation des Membres Supérieurs of Personnes Âgées*
TCLE	Termo de Consentimento Livre e Esclarecido
UFCSPA	Universidade Federal de Ciências da Saúde de Porto Alegre
UFSM	Universidade Federal de Santa Maria

SUMÁRIO

1 INTRODUÇÃO E JUSTIFICATIVA DO ESTUDO 6
2 REFERENCIAL TEÓRICO 7
3 PROBLEMA DE PESQUISA 20
4 HIPÓTESES 20
4.1 Hipótese Nula 20
4.2 Hipótese Alternativa 20
5 OBJETIVOS 20
5.1 Objetivo Geral 20
5.2 Objetivos Específicos 20
6 METODOLOGIA DO ESTUDO 21
6.1 Delineamento 21
6.2 Área de Abrangência 21
6.3 Local da pesquisa 21
6.4 População e amostra 22
6.5 Critérios de Elegibilidade 22
6.5.1 Critérios de Inclusão 22
6.5.2 Critérios de exclusão 22
6.6 Instrumentos de avaliação 23
6.6.1 Escala de Hoehn e Yahr 23
6.6.3 Questionário de Imagens Cinestésicas e Visuais 23
6.6.4 *9-Hole Peg Test* 24
6.6.5 Escala de Avaliação da Doença de Parkinson Unificada da Sociedade de Distúrbios do Movimento 25
6.6.6 *Test D'évaluation Des Membres Supérieurs Des Personnes Âgées* 25
6.6.7 Parkinson's Disease-Cognitive Rating Scale 26
6.6.8 Medida Canadense de Desempenho Ocupacional 27
6.7 Desfechos 27
6.7.1 Desfecho Principal 27
6.7.2 Desfecho Secundário 28
6.8 Procedimento de coleta de dados 28
6.8.1 Etapas do Estudo 28
6.8.2 Proposta de Intervenção 28
6.9 Análise de Dados 30
6.10 Cálculo Amostral 31
6.11 Procedimentos éticos e legais 31
6.12 Riscos e Benefícios 31
7 CRONOGRAMA DE ATIVIDADES 33
8 ORÇAMENTO 34
REFERÊNCIAS 35
APÊNDICE 1 – AVALIAÇÃO INICIAL 44
APÊNDICE 2 - TERMO DE CONSENTIMENTO LIVRE ESCLARECIDO 45
ANEXO 1 – ESTÁGIOS DA DOENÇA DE PARKINSON CONFORME HOEHN E YAHR 45
ANEXO 2 – MONTREAL COGNITIVE ASSESSMENT 50
ANEXO 3 – QUESTIONÁRIO DE IMAGINAÇÃO CINESTÉSICAS E VISUAL 51
ANEXO 4 - TEMPO MÉDIO DE REALIZAÇÃO DA TAREFA CONFORME OS ESTÁGIOS DA DOENÇA DE PARKINSON 52

ANEXO 5 - AVALIAÇÃO DA DOENÇA DE PARKINSON UNIFICADA DA SOCIEDADE DE DISTÚRBIOS DO MOVIMENTO 53
ANEXO 6 - TEST D'ÉVALUATION DES MEMBRES SUPÉRIERS DE PERSONNES AGÉES 59
ANEXO 7 – PARKINSON'S DISEASE-COGNITIVE RATING SCALE 60
ANEXO 8 - MEDIDA CANADENSE DE TERAPIA OCUPACIONAL 73

1 INTRODUÇÃO E JUSTIFICATIVA DO ESTUDO

Sabendo que é progressiva e degenerativa, a doença de Parkinson (DP) torna a pessoa incapacitante por suas complicações motoras e não motoras (NIELSEN et al., 2020). Os seus sintomas têm reflexos negativos no controle motor e no planejamento motor, interferido no uso das extremidades superiores para realização das atividades diárias (BEK et al., 2016). Lembrando, também, que sintomas não motores, especialmente o cognitivo, também interfere na realização das atividades diárias que são envolvidas por múltiplos estímulos, incapacitando a pessoa com DP (KOBAYASHI et al., 2022).

Mesmo com a existência de tratamento farmacológico, tem-se o problema da regulação das dosagens e do tempo de uso em combinação com a progressão da doença (ABRAMI et al., 2020). Sabe-se que há outras possibilidades de tratamento, sendo algumas invasivas, como a cirurgia de estimulação cerebral profunda. Já a reabilitação busca complementações usando diferentes recursos, além do uso de abordagens alternativas associados com exercícios físicos (ABBRUZZESE et al., 2015).

No entanto, considerando a interação entre componentes cognitivos e componentes motores na DP, também é importante considerar tais alterações desde a avaliação até a reabilitação. Mesmo sendo uma possibilidade recomendada e promissora na reabilitação da DP, a imaginação motora ainda é pouco pesquisada, tornando as evidências fracas e os protocolos limitados. No mesmo sentido, a observação da ação segue sendo pouco explorada na reabilitação, especialmente nos membros superiores na DP. Uma vez que ambas as abordagens para a DP são mais direcionadas para as alterações na marcha e na estabilidade postural. E mesmo sendo uma doença que causa dificuldades nos movimentos das mãos, são poucos os estudos envolvendo uma abordagem terapêutica nos membros superiores (BEK et al., 2021).

Sendo assim, a elaboração do projeto de pesquisa se justifica pela originalidade em investigar os efeitos da imaginação motora e da observação da ação nas alterações motoras em membros superiores e cognitivas na DP como possibilidade de uso na prática também aplicada na reabilitação de pessoas com DP, podendo-se inspirar a elaboração de protocolos de intervenção. Não obstante, não se encontra estudos abordando sobre o uso isolado da prática da imaginação motora, sendo a aplicabilidade associada com outros tipos de intervenção, inclusive na DP, tampouco sobre a associação dos efeitos com a observação da ação em membros superiores, especialmente na função das mãos. Ressaltando, então, que nos estudos anteriores o treinamento é realizado separadamente (BEK et al., 2021).

Além do mais, tem-se apostado nas tecnologias de forma acessiva associada com a reabilitação, como o caso da interface entre cérebro e computador. Quanto ao uso do exoesqueleto na extremidade do membro superior, tem-se a aplicação voltada para outras complicações neurológicas, sendo um diferencial do estudo a interferência nos sintomas da DP. Logo, o exoesqueleto também é uma possibilidade promissora na DP para ativação dos neurônios e, consequentemente, recuperação do movimento.

Assim, o tema do projeto de pesquisa envolve os efeitos da imaginação motora e da observação da ação nas alterações motoras em membros superiores e cognitivas na DP, bem como o uso do exoesqueleto, como possibilidades para reabilitação.

2 REFERENCIAL TEÓRICO

A doença de Parkinson

Dentre os males crônicos potencialmente incapacitantes, a doença de Parkinson (DP) se configura como uma das principais causas neurológicas. Como se sabe, a DP é degenerativa, ocorre de forma lenta e progressiva, sendo idiopática e envolve um conjunto de fatores (CIKAJLO; PETERLIN, 2019; NIELSEN et al., 2020). Afeta, aproximadamente, 7 a 10 milhões de pessoas em todo o mundo, iniciando, geralmente, na idade adulta entre 35 e 60 anos (CIKAJLO; PETERLIN, 2019). É a segunda doença neurodegenerativa mais comum que afeta os idosos (PONDÉ et al., 2019): entre 0,5% e 1% da população com idades entre 65 e 69 anos e de 1% a 3% da população com mais de 80 anos (FLORIANO et al., 2015).

Em condição normal, o mesencéfalo tem a função de processar informações visual e auditiva, bem como emitir respostas somáticas voluntárias. Na substância negra, que tem células pigmentadas, os neurônios excitadores estão inativos pela ação dos efeitos da dopamina (PAUL et al., 2020). A dopamina é um neurotransmissor que facilita e modula a atividade dos neurônios (ABRAMI et al., 2020), tendo como função a regulação da eferência motora dos núcleos de base – responsável pela coordenação e controle motor, retroalimentação e contrações musculares. Na DP ocorre a degeneração dos neurônios produtores de dopamina (neurônios dopaminérgicos) na substancia negra, dentro dos gânglios da base (PAUL et al., 2020). Ocorrendo, então, a ativação dos neurônios excitadores, manifestando-se através da desregulação do controle motor (ABRAMI et al., 2020).

A DP é caracterizada por sintomas não motores e motores (ABBRUZZESE et al., 2015), sendo causas de implicações negativas na qualidade de vida, refletindo diretamente em várias

situações cotidianas da pessoa com DP. Os sintomas não motores são considerados como sintomas adicionais e também são frequentes (FERREIRA et al., 2018). Os mesmos são percebidos como: diminuição de expressão facial, disfagia e disartria, além de alterações emocionais como ansiedade e depressão (SOUSA; MACEDO; BRUCKI, 2021). Dentre os sintomas não motores, tem-se também a prevalência de demência e de comprometimento cognitivo leve (ROSCA; SIMU, 2020). Os problemas cognitivos mais comumente observados na DP são nos seguintes domínios: concentração e na atenção, memória de trabalho (NIELSEN et al., 2020) e para fatos recentes, bem como dificuldades para cálculos e atividades de orientação espacial, e funções executivas (SOUSA; MACEDO; BRUCKI, 2021). Os sintomas não-motores têm reflexo negativo no desempenho ocupacional, podendo apresentar dificuldades em atividades como dirigir, fazer compras, manutenção doméstica; assim como no autocuidado: vestir-se e tomar banho (KOBAYASHI et al., 2022).

Os principais sintomas motores são (ABRAMI et al., 2020): a) tremor de repouso - ocorre em repouso e diminui quando se tenta o movimento voluntário; b) rigidez - aumento simultâneo do tônus muscular, podendo apresentar uma resistência constante ao longo da amplitude de movimento quando o movimento é lento e progressivo ou um movimento ritmicamente resistido ao longo da amplitude de movimento; c) acinesia - dificuldade para iniciar o movimento; d) bradicinesia - lentidão para manter o movimento.

NA DP tem-se a manifestação de diferentes tipos de tremor que estão acordo com as circunstâncias, a parte do corpo envolvida e a frequência com que o tremor ocorre (HELMICH et al., 2012). O tremor nesta patologia é um movimento rítmico involuntário, tipicamente caracterizado por tremor de repouso unilateral, que acontece nas extremidades superiores, especialmente nas mãos (MALLING et al., 2019). Ressalta-se, que o tremor é um marcador importante da DP, pois há indicações de que o tremor dominante sugere uma progressão da doença relativamente lenta, pela menor disfunção dopaminérgica (HELMICH et al., 2012).

Mesmo durante os movimentos lentos, observa-se um movimento de tremor (de cerca de 7 Hz) nos membros superiores (VAN DEN NOORT et al., 2017). No entanto, também pode ocorrer na cabeça, membros inferiores e até mesmo tronco, causando problemas de desempenho (HU et al., 2019). Identifica-se que pessoas com DP com tremor têm significativamente mais tremor de repouso e postural, mas as pessoas com DP sem tremor têm significativamente mais sintomas axiais e de marcha, bem como sintomas relacionados à fala e hipomímia (HELMICH; BLOEM; TONI, 2012).

O tremor de repouso é inibido durante o movimento e pode reaparecer com a mesma frequência ao adotar uma postura ou mesmo ao se movimentar (HELDMAN et al., 2011). Com

uma frequência um pouco mais alta, mas sendo menos comum entre os casos (maior que 1,5 Hz), tem-se o tremor essencial. O tremor essencial ocorre mais nos antebraços e nas mãos, sendo mais problemático durante o movimento voluntário ou ao manter a postura contra a gravidade, o que afeta a realização das atividades da vida diária, sendo que as mais afetadas são: escrever à mão, comer, vestir-se e cuidar de si (HELDMAN et al., 2011). Há, também, os tremores posturais e cinéticos isolados, com uma frequência ainda maior (maior que 4 Hz) (HELMICH et al., 2012).

Tem-se uma sinalização de que o movimento devido ao tremor deve ser discriminado do movimento voluntário antes que a quantificação do tremor seja possível, sendo que o movimento voluntário ocorre em frequências baixas (<3Hz), enquanto o tremor ocorre em frequências mais altas (>4Hz) (HELDMAN et al., 2011). Assim, em relação à condição de tarefa dinâmica, permanece desconhecido como o tremor pode interferir no desempenho do movimento voluntário, como o alcance rápido, o que impede a elaboração de estratégias de reabilitação não invasivas para pessoas com DP com tremor com base na supressão do tremor em repouso (HU et al., 2019). Pensando em um exemplo cotidiano, a atividade de dobrar a roupa é um movimento de forma livre que não segue um padrão rítmico, mas o tremor ainda pode ser distinguido do movimento voluntário devido à nitidez do pico espectral do tremor, em que os movimentos contêm componentes de frequência múltipla (HELDMAN et al., 2011).

Por sua vez, a rigidez é um dos sintomas cardinais da DP e inclui informações da gravidade, da distribuição e se está presente em repouso e em estado não medicamentoso (ZETTERBERG et al., 2015). A mesma depende da velocidade angular e da amplitude articular da mobilização aplicada (FERREIRA-SÁNCHEZ; MORENO-VERDÚ; CANO-DE-LA-CUERDA, 2020), ou seja, com uma excursão articular aumentada e maior estiramento muscular, o reflexo de estiramento domina a reação de encurtamento, resultando em maior rigidez (POWELL et al., 2011). O que também ocorre na DP, é a dificuldade em manter o relaxamento dos músculos não utilizados em uma tarefa, aumentando, assim, a rigidez durante a manobra de ativação contralateral (ZETTERBERG et al., 2015).

As pessoas com DP têm maiores valores de rigidez, tanto em repouso quanto durante a mobilização passiva, pois existe o aumento do tônus nos músculos em termos de atividade eletromiográfica, resposta à deformação biomecânica e resistência ao estiramento, que pode ser detectada com ferramentas instrumentalizadas (FERREIRA-SÁNCHEZ; MORENO-VERDÚ; CANO-DE-LA-CUERDA, 2020). Em um estudo, houve a identificação, pelo uso do modelo biomecânico, do aumento do componente neural da resistência passiva ao movimento nos músculos flexores da mão e dos dedos em indivíduos com DP (ZETTERBERG et al., 2015).

O efeito da amplitude de movimento é muito menos compreendido e raramente investigado na rigidez parkinsoniana. No entanto, em conjunto com as contribuições segmentares para a rigidez, obteve-se que os mecanismos supraespinhais provavelmente são responsáveis pela natureza dependente da amplitude de movimento da rigidez na DP (POWELL et al., 2011). Tanto a amplitude do movimento quanto a velocidade do movimento são moduladoras da rigidez parkinsoniana, servindo como parâmetros para avaliar a rigidez usando movimentos articulares passivos aplicados manualmente (POWELL et al., 2012), sendo que pessoas com DP têm maior resistência ao alongamento passivo (ZETTERBERG et al., 2015). Lembrando, assim, que a redução da amplitude de movimento pode ser uma consequência debilitante da DP, afetando atividades cotidianas, como escrever (BEK et al., 2018).

O componente neural é o maior contribuinte para a resistência passiva ao movimento na DP. Em relação à mão menos afetada, a DP apresenta-se com aumento da resistência total e do componente neural em condição dinâmica; já em relação à mão mais afetada, a DP apresenta-se com aumento da resistência total e componente neural nas condições passivas e dinâmicas (ZETTERBERG et al., 2015).

Em relação à bradicinesia, a mesma se refere à dificuldade para ajustar a posição do corpo, iniciar e executar o movimento e realizar movimentos sequenciais e simultâneos. Logo, a bradicinesia envolve o comprometimento global da mobilidade multiarticular durante a realização de uma tarefa que envolve muitos segmentos do corpo, e não como movimentos isolados em algumas articulações (MEMAR et al., 2018). A fadiga também é comum na DP, causando sérios efeitos negativos na qualidade de vida e pode interferir na mobilidade (ABBRUZZESE et al., 2015).

As pessoas com DP geralmente têm dificuldade em iniciar os movimentos, sendo os movimentos mais lentos e reduzidos em tamanho (BEK et al., 2016). As alterações da DP nos membros superiores causam perturbações na destreza, motricidade grossa e fina (NIELSEN et al., 2020). Ocorre, também, dificuldades no planejamento das características temporais dos movimentos sequenciais dos dedos quando o intervalo interestímulo entre dois movimentos consecutivos está na faixa supra-segundo, e que as dificuldades no planejamento motor se refletem no desempenho motor anormal (AVANZINO et al., 2013). Com a redução do balanço do membro superior e perda de movimentos desassociados do membro superior e do tronco, ocorre a instabilidade na marcha (BARBOSA et al., 2016). Há também o reflexo dos sintomas motores causando distúrbios da marcha, principalmente a marcha festinante, em que o comprimento da passada é menor e arrastada com velocidade aumentada, e também a marcha congelada em que ocorre congelamento do andar, podendo também ter instabilidade postural –

o que aumenta o risco de quedas (SHULMAN; JAGER; FEANY, 2011; ABBRUZZESE et al., 2015).

Há indicações de que as alterações nos sintomas motores na DP não estão diretamente relacionadas às alterações na rede somatomotora, indicando que a DP não é apenas um distúrbio do movimento, afetando também várias redes neurais (MYERS et al., 2018). O que implica na qualidade de vida em todas as fases da doença (FERREIRA et al., 2018; CIKAJLO, PETERLIN, 2019), diante do registro da dificuldade na realização da maioria das atividades cotidianas: cuidados pessoais, vestir-se, atividades de trabalho e atividades de lazer e afazeres domésticos, pela relação com a coordenação motora fina e a destreza, bem como coordenação bimanual, hipocinesia e iniciação (BEK et al., 2016).

Os tratamentos para a doença de Parkinson

Atualmente, não há tratamento aprovado que altere a taxa de progressão da DP (ABRAMI et al., 2020), sendo que as possibilidades estão concentradas na atenuação dos sintomas, uma vez que não existe cura conhecida. Logo, o manejo da DP é tradicionalmente com base no tratamento sintomático (ABBRUZZESE et al., 2015).

A degeneração dos neurônios dopaminérgicos, desencadeantes das alterações na rede dos gânglios da base, é tratada principalmente com medicação, como a levodopa ou antagonista dopaminérgico (CIKAJLO, PETERLIN, 2019), sendo uma terapia de reposição de dopamina, que compensa a falta da mesma produzida endogenamente (ABRAMI et al., 2020). A prescrição da quantidade adequada do medicamento é importante, pois a dosagem não suficiente leva a um controle insuficiente dos sintomas; já o excesso do medicamento leva a uma habituação mais rápida e a discinesias potencialmente incapacitantes (ABRAMI et al., 2020). Em contra partida, com a progressão da doença, a ação da levadopa diminui, tendo uma menor resposta medicamentosa com o tempo (CIKAJLO, PETERLIN, 2019). Lembrando, assim, que a falta de dopamina resulta em movimentos mais lentos, sendo que na DP com tremor se tem uma capacidade reduzida de controle motor (HU et al., 2019).

Como a DP é progressiva, há necessidade de alterações no tratamento e de monitoramento contínuo de longa duração, envolvendo um plano personalizado para manter o controle dos sintomas, bem como evitando os efeitos colaterais dos medicamentos (SHAWEN et al., 2020). Com o uso da medicação, percebe-se uma melhora em todos os parâmetros quanto aos movimentos de abertura e fechamento da mão, como o aumento da velocidade e amplitude de movimento (VAN DEN NOORT et al., 2017). Já na condição sem a medicação, há uma

irregularidade do movimento, como nos movimentos de pronação e supinação, dificultando a realização em vista da pequena amplitude de movimento (VAN DEN NOORT et al., 2017).

A manipulação de objetos depende da coordenação fina da mão e dos dedos e não pode ser restaurada pelos níveis de dopamina, uma vez que a destreza dos dedos é insensível ao tratamento dopaminérgico (CIKAJLO, PETERLIN, 2019). Assim sendo, a dopamina exógena - medicação de reposição - pode ter efeitos positivos nas habilidades motoras das extremidades superiores, existentes com base no tipo de tarefa e recursos de prática, na DP leve a moderada. Porém, tem-se efeitos potencialmente prejudiciais na (re) aprendizagem de habilidades motoras no contexto da reabilitação (PAUL et al., 2020).

Os sintomas motores têm respostas diretas à medicação dopaminérgica e à estimulação cerebral profunda e, portanto, são frequentemente usados para julgar os efeitos de tais terapias (VAN DEN NOORT et al., 2017). Tanto que a avaliação do toque com os dedos, incluindo velocidade, amplitude e ritmo, são contribuintes para a discriminação da DP (ENDO et al., 2011).

A reabilitação convencional, associada com outras possibilidades de intervenção, bem como a prática de exercícios físicos, são importantes para a manutenção das alterações motoras e não motoras, complementando o tratamento farmacológico (ABBRUZZESE et al., 2015). Há também o uso de estratégias não convencionais, como musicoterapia e dança e artes marciais (ABBRUZZESE et al., 2015). No entanto, mesmo a reabilitação induzindo benefícios de curta duração, clinicamente importantes, principalmente para marcha e equilíbrio, as intervenções são amplamente heterogêneas, e ainda não há consenso sobre a abordagem ideal (ABBRUZZESE et al., 2015). Mas se sabe que, mesmo considerando a recuperação motora, a maioria das abordagens não envolve a organização do sistema motor (BUCCINO, 2014).

Assim, a reabilitação deve estar direcionada à prática e ao aprendizado de atividades específicas das áreas centrais que são prejudicadas, como as atividades manuais, levando a um melhor desempenho nas atividades da vida diária (ABBRUZZESE et al., 2015). Logo, as terapias para DP devem ser direcionadas para melhorar ou manter o controle do movimento, como facilitar o início e aumentar a amplitude ou velocidade (BEK et al., 2016). Como a neuroplasticidade é amplamente dependente da intensidade, repetição, especificidade, dificuldade e complexidade da prática, lembra-se que o tempo para alcançar um aprendizado e automatização eficazes é maior na DP (ABBRUZZESE et al., 2015).

Imaginação Motora e Observação da Ação

A imaginação motora (IM) e a observação de ação (OA) são duas abordagens inovadoras de reabilitação que são viáveis em diversas condições patológicas, mesmo existindo estudos há muito tempo com pessoas saudáveis (CALIGIORE et al., 2017). Assim, tem-se o investimento de aplicação de ambas como uma ferramenta na neurorreabilitação (BUCCINO, 2014), sendo na DP potencialmente capaz de induzir benefícios significativos (ABBRUZZESE et al., 2015).

No entanto, a maioria dos estudos realizados são do uso das abordagens de maneira isolada ou associadas com outras práticas de reabilitação. No caso da IM, tem-se estudos voltados para o tratamento de sintomas motores, especialmente com o acidente vascular cerebral nas fases agudas e crônicas (CALIGIORE et al., 2017). Em uma revisão sistemática da literatura, para investigar os protocolos de prática mental na reabilitação motora na DP, houve a identificação de apenas quatro estudos (BRAUN et al., 2011; TAMIR; DICKSTEIN; HUBERMAN, 2007; EL-WISHY, FAYEZ, 2013; SANTIAGO et al., 2015) com o uso associado da imaginação motora com a reabilitação convencional, sendo utilizada pela maioria a imaginação motora visual ou visual e cinestésica, tendo como principal objetivo a melhora da mobilidade e marcha (SILVA et al., 2016). Já no caso da OA, há o envolvimento dos estudos com outras patologias neurológicas como o acidente vascular cerebral (BUCCINO, 2014) e paralisia cerebral, bem como patologias ortopédicas pós-cirurgicas (CALIGIORE et al., 2017; SARASSO et al., 2015).

A partir de uma revisão sistemática, dos 25 artigos selecionados, não houve o registro de nenhum estudo envolvendo a investigação da ação conjunta da IM e da OA de longo prazo, e apenas um comparando o efeito de ambas em sessão única, mas o restante se tratava do efeito da OA ou da IM, isoladas, em um experimento de sessão única, ou o efeito apenas da OA ou apenas da IM como tratamento de longo prazo (CALIGIORE et al., 2017). Outro apontamento relevante é que o uso das abordagens na DP estão mais direcionados para os sintomas motores em membros inferiores, como alterações de equilíbrio e marcha (PELOSIN et al., 2010; KIKUCHI et al., 2014).

O acionamento da OA ocorre por estímulos visuais de origem externa e na IM por estímulos internos - reativação de uma representação motora armazenada na memória (CALIGIORE et al., 2017). O treinamento de IM é tão eficaz quanto o de execução motora na primeira fase de aprendizagem, ou seja, pode induzir melhorias no desempenho motor e, portanto, nos processos de aprendizagem motora (ABBRUZZESE et al., 2015). Porém, para que ocorra a consolidação e retenção das habilidades motoras de forma eficaz, sugere-se o fornecimento de um estímulo sensorial externo (BONASSI et al., 2020), como a OA. Assim,

pode-se mencionar que existe um compartilhamento entre a OA e a IM como uma reprodução interna do comportamento, que melhora o aprendizado e os traços neurais das ações motoras (CALIGIORE et al., 2017).

Aponta-se, então, que pode ocorrer um aumento dos efeitos comportamentais e neurais da OA com a IM, por ativar o sistema motor, influenciando o movimento e aumentando o aprendizado, demonstrando que a IM melhora a velocidade e o tempo de movimento em pessoas com DP (BEK et al., 2018) e a OA a velocidade e precisão das ações (ABBRUZZESE et al., 2015). A atividade cortical relacionada à execução de movimentos observados e imaginados são induzidas por simulações motoras através da OA e da IM (KANEKO et al., 2021), recrutando processos cerebrais de alto nível envolvidos no comportamento motor (CALIGIORE et al., 2017).

Existe um apontamento de que OA é melhor do que a IM como estratégia para aprender uma nova tarefa motora complexa, pelo menos no início da fase rápida de aprendizagem motora (GATTI et al., 2013; BUCCINO, 2014). No entanto, a combinação da OA e IM aumenta a imitação na DP, sendo uma abordagem terapêutica promissora (BEK et al., 2018), por ajudar as pessoas com DP nas suas atividades da vida diária e no manejo dos sintomas (BEK et al., 2016), uma vez que as ações são apresentadas no contexto da vida cotidiana (GATTI et al., 2013). Assim, a IM e a OA usadas como programas terapêuticos podem melhorar as habilidades motoras, aumentando os sinais proprioceptivos normalmente gerados durante os movimentos (ABBRUZZESE et al., 2015), ou retardar a deterioração das capacidades motoras na DP (CALIGIORE et al., 2017).

A Imaginação Motora

A imaginação motora (IM) é um processo cognitivo que envolve a capacidade de realizar uma ação mentalmente, sem necessidade da realização do movimento em si (ABBRUZZESE et al., 2015; BEK et al., 2016). É definida como a possibilidade de imaginar uma ação motora sem executá-la fisicamente (SILVA et al., 2016), envolvendo o processo de criação de experiências visuais, auditivas ou cinestésicas na mente (ABRAHAM et al., 2018). Sendo que a capacidade de simular mentalmente um determinado movimento está estritamente ligada à correspondência do movimento com o repertório motor pessoal (AVANZINO et al., 2013).

Mesmo quando ainda inconclusiva, especialmente quanto ao desconhecimento da base neural do efeito reabilitador na DP, a IM já era considerada promissora (FISCHER et al., 2017).

A IM usa as mesmas representações motoras internas que os movimentos realizados em um nível neural específico (PONDÉ et al., 2019): parte ventral e dorsal do córtex pré-motor, área motora suplementar, córtex cingulado anterior, lóbulo parietal superior e lóbulo parietal inferior, gânglios da base e cerebelo. Assim, com uma melhor compreensão da atividade de rede subjacente às imagens motoras, houve a possibilidade de informar a melhor forma de aproveitamento do potencial terapêutico da IM adjuvante à reabilitação em pessoas parkinsonianas (FISCHER et al., 2017).

Na IM têm experimentos explícitos e implícitos com relações divergentes com a cognição motora (DI RIENZO et al., 2014). A perspectiva que a pessoa usa para imaginar pode ser: perspectiva interna (primeira pessoa - imagina ela mesma), a qual se relaciona com a visão da pessoa sobre o conteúdo das imagens ou com a sua sensação cinestésica - a pessoa imagina o movimento sendo realizado, como se sentisse o movimento da ação; ou perspectiva externa (terceira pessoa - imagina outra pessoa), a qual se relaciona com a imaginação visual de cenas fora da pessoa (ABBRUZZESE et al., 2015; SILVA et al., 2016).

Sobre a perspectiva interna, é interessante mencionar que as imagens visuais são consideradas mais fáceis do que as imagens cinestésicas, pois envolve a prática de imagens mentais (HEREMANS et al., 2012). Inclusive, houve a identificação do uso em 85% dos estudos de uma revisão sistemática da IM visual em primeira pessoa, às vezes combinada com informações cinestésicas (DI RIENZO et al., 2014). Logo, sugere-se o aprendizado primeiramente da IM visual por envolver tarefas que enfatizam forma, e depois incorporar a IM cinestésica para as tarefas que enfatizam tempo ou coordenação das mãos (FÉRY, 2003; SILVA et al., 2016). A partir da IM realizada visualmente ou cinestesicamente, tem-se uma preparação para as próximas sequências de movimento (HEREMANS et al., 2012), facilitando efetivamente o aprendizado de habilidades (BONASSI et al., 2020).

Como a execução motora, o treinamento de IM pode induzir melhorias no desempenho motor e, portanto, nos processos de aprendizagem motora na DP (ABBRUZZESE et al., 2015). Logo, a IM está sendo vista como um novo método de reabilitação promissor para pessoas com distúrbios neurológicos, principalmente em doenças cerebrovasculares, tendo relevância e potencialidade para a DP (ABRAHAM et al., 2018), ajustando-se às limitações motoras, como diminuição da amplitude ou velocidade do movimento (HEREMANS et al., 2012). Apesar dos poucos estudos na DP, há evidências que a prática mental pode reduzir a bradicinesia, melhorar a mobilidade e a velocidade da marcha (SILVA et al., 2016), bem como melhorar a estabilidade dinâmica (SANTIAGO et al., 2015), além de não apresentar tremor na condição com medicação durante o repouso e durante a tarefa mental (VAN DEN NOORT et al., 2017).

A capacidade das pessoas com DP de imaginar movimentos de forma eficiente ainda é controverso. Embora o uso da levodopa tem sido sugerido para normalizar a atividade cerebral em vários áreas corticais (incluindo a área motora suplementar), pessoas com DP são capazes de imaginar de forma semelhante aos adultos mais velhos tanto sob efeito ou não do medicamento (ABBRUZZESE et al., 2015). Lembra-se que a realização de tarefas de imagens é mais lenta na DP, mas a nitidez e a precisão das imagens motoras estão preservadas (HEREMANS et al., 2012). Ressalta-se, também, uma vantagem importante da IM é usá-la para praticar movimentos potencialmente perigosos de maneira segura (HEREMANS et al., 2011; HEREMANS et al., 2012).

A Observação da Ação

A observação da ação consiste na observação de uma outra pessoa realizando uma ação, ou tarefa motora, em vídeo ou em tempo real (SARASSO et al., 2015). Existe o reconhecimento de que, ao observar a realização de uma ação por outra pessoa, ocorre uma ativação no cérebro nas mesmas estruturas neurais usadas para a execução real das mesmas ações, sendo recrutadas no cérebro de quem observa como se realmente realizasse a ação observada (BUCCINO, 2014; ABBRUZZESE et al., 2015). Assim, a OA desperta um mecanismo de espelho que atende à capacidade do cérebro para acoplar uma ação observada com sua contraparte motora no cérebro de quem observa (GATTI et al., 2013).

Na OA ocorre o recrutando de áreas específicas nos lobos frontal e parietal de forma semelhante ao que acontece durante execução motora: córtex pré-motor ventral, parte posterior do giro frontal inferior, parte rostral do lóbulo parietal inferior e sulco temporal superior posterior (ABBRUZZESE et al., 2015). Assim, o que antes era uma preocupação de que a OA não causava um reparo dos circuitos neurais subjacentes, já tem-se um outro panorama de que usada como abordagem de neurorreabilitação tem a possibilidade de restaurar as estruturas neurais ou ativar vias suplementares ou relacionadas para realizar as funções originais (BUCCINO, 2014).

Na OA pode-se mostrar ações diárias, fornecendo informações para realização das mesmas em contextos da vida (BUCCINO, 2014). A OA é uma maneira eficaz de aprender ou melhorar o desempenho de uma habilidade motora, modificando a velocidade e precisão das ações na DP (ABBRUZZESE et al., 2015). Sabe-se que pessoas com DP tem a possibilidade de ajustar a amplitude dos movimentos das mãos em resposta às ações observadas, indicando o potencial de intervenções baseadas na OA para aumentar a amplitude assim como a velocidade

dos movimentos (BEK et al., 2018). A observação de movimentos rápidos e rítmicos dos dedos induz implicitamente um aumento da taxa de movimento espontâneo dos dedos em pessoas com DP, melhorando assim a bradicinesia (PELOSIN et al., 2013). O uso de OA na DP melhora a taxa de movimento espontâneo dos movimentos dos dedos auto-ritmados, o que reflete na melhora na realização das atividades da vida diária (SARASSO et al., 2015).

A Interface Cérebro Máquina

O termo Interface Cérebro–Máquina (ICM), que em inglês é chamado *Brain Computer Interface* (BCI), refere-se a sistemas que capturam os sinais de atividades cerebrais do indivíduo, traduzindo-os em comandos computadorizados para controlar dispositivos externos, que podem ser dispositivos de comunicação (SELLERS; DONCHIN, 2006), estimulação elétrica funcional (FES) (LI et al., 2014) ou exoesqueletos robóticos (ANG et al., 2015), entre outros. A tecnologia ICM é relativamente nova, e permite a interação da pessoa com o ambiente através de sinais cerebrais e possam restaurar a função motora por induzir a plasticidade cerebral (ANG et al., 2011).

Para a captura destes sinais cerebrais, estratégias invasivas e não invasivas podem ser usadas. Nas ICM invasivas podem-se adquirir os sinais espaço-temporais e ter uma maior capacidade de distinguir as dimensões de intenção do indivíduo através de eletrodos posicionados na superfície do cérebro (eletrocorticografia ou ECoG) ou implantados dentro do córtex cerebral (microeletrodos) (MILLER et al., 2010; ALIMARDANI; NISHIO; ISHIGURO, 2016; PFURTSCHELLER; NEUPER, 2006; BUCH et al., 2008). Nos sistemas não-invasivos, os eletrodos são posicionados na calota craniana, usando sinais coletados por eletroencefalografia (EEG), magnetoencefalografia (MEG), espectroscopia funcional de infra-vermelho (fNIRS). No entanto, as interfaces cérebro-máquina não invasivas podem ser mais promissoras que estratégias invasivas devido à segurança e por questões éticas (BIRBAUMER; COHEN, 2007).

Entre as citadas acima, a ICM baseada nos sinais cerebrais captados através do EEG é o sistema mais comumente utilizado por ser um equipamento mais simples e barato (WOLPAW et al., 2002). Na ICM típica, através de EEG, a intenção do movimento do indivíduo (imagética motora ou execução) é decodificada em tempo-real por meio da atividade elétrica cerebral em andamento, extraindo desta as características relevantes. E a detecção de intenção de movimento seria desencadear um feedback sensorial contingente para o usuário

(ALIMARDANI; NISHIO; ISHIGURO, 2016; PFURTSCHELLER; NEUPER, 2006; BUCH et al., 2008)

As ICM são atualmente utilizadas principalmente em duas aplicações, a primeira em tecnologias assistivas, que visam restaurar as funções perdidas como, por exemplo, para a comunicação na Síndrome do encarceramento (PFURTSCHELLER; LOPES DA SILVA, 1999) ou na paralisia dos movimentos, em tetraplegias, usando atuadores robóticos e/ou sistema de estimulação elétrica funcional para a tarefa cotidiana de alimentação (ZHANG et al., 2018). A segunda, em tecnologias de reabilitação, também chamada Interface Cérebro-Máquina reabilitativa ou neurofeedback (BANIQUED et al., 2021) que visa promover a neuroplasticidade através manipulação ou autorregulação da atividade neurofisiológica para facilitar a recuperação motora.

Nos últimos cinco anos, diversas pesquisas foram desenvolvidas na área da reabilitação neurológica, sendo a maioria dos estudos voltados para a Interface Cérebro-Máquina e acidente vascular cerebral (AVC), com interesse em promover a recuperação funcional do membro superior, por ser frequentemente e amplamente acometido e gerar uma grande limitação em atividades de vida diária (LAWRENCE et al., 2001; MONGE-PEREIRA et al., 2017). Uma revisão de Mehrhols e colaboradores em 2018 selecionou ensaios clínicos randomizados que utilizaram o treinamento eletromecânico do membro superior assistido por robô para melhorar as atividades da vida diária, função e força muscular do braço em pacientes após sofrerem AVC (MEHRHOLZ et al., 2018). O resultado encontrado incluiu 45 estudos envolvendo 1.619 participantes, comparando treinamento de braço eletromecânico assistido por robô para recuperação da função do braço com outras intervenções de reabilitação ou placebo, ou nenhum tratamento. O treinamento com o braço eletromecânico assistido por robô promoveu melhora significativa nas atividades de vida diária, na função e na força muscular de braço. No entanto, os resultados devem ser interpretados com cautela, embora a qualidade da evidência tenha sido alta, pois precisam ser observadas variações entre os ensaios nos aspectos de intensidade, duração e quantidade de treinamento; tipo de tratamento; características dos participantes.

A assistência de tecnologias robóticas no processo de recuperação funcional está proporcionando um avanço em pesquisas com a ICM, principalmente nas correlacionadas ao AVC. Contudo, nota-se que em relação às demais patologias neurológicas há poucos estudos relacionados a esta tecnologia da reabilitação. Dentre inúmeras disfunções neurofuncionais, a doença de Parkinson (DP) poderia se beneficiar mais com esta tecnologia, pois distúrbios do movimento dos membros superiores podem estar presentes desde o diagnóstico, apresentando muitas vezes como sintoma inicial da doença a micrografia ou tremor de repouso (DICKSON;

GRU¨NEWALD, 2004), dificuldade da velocidade e destreza na manipulação de objetos (GEBHARDT et al., 2008; PROUD; MORRIS, 2010; MANSON; CAIRD, 1985). À medida que a doença progride, esses distúrbios dos membros superiores podem levar a limitações maiores no trabalho, na recreação e nas tarefas diárias como vestir-se e comer (STURKENBOOM et al., 2011), impactando a qualidade de vida e funcionalidade desses indivíduos.

Yongbin e colaboradores em 2015, investigaram os padrões de conectividade funcional em estado de repouso de todo o cérebro de pacientes com doença de Parkinson (DP) através de técnicas de reconhecimento de padrões e de neuroimagem que resultaram no fornecimento de informações adicionais para o diagnóstico clínico e avaliação do tratamento da doença. Outro interessante estudo foi o de Hanson e colaboradores em 2012, com a aplicação de ICM invasiva, em que 25 pacientes que apresentavam tremor essencial ou doença de Parkinson foram submetidos a neurocirurgia para colocação de implantes terapêuticos de Estimulação Cerebral Profunda em um conjunto de neurônios na região subcortical, sendo as atividades destes registradas durante a realização de uma tarefa motora de rastreamento de alvos usando um cursor controlado por uma luva háptica. Foi observado que as modulações na taxa de disparo de um número substancial de neurônios representaram o início do alvo, o início/direção do movimento e o tremor da mão. Notavelmente, todos os neurônios associados ao tremor exibiram sincronia dentro do conjunto, sendo que o conjunto de neurônios subcorticais são praticamente inexplorados, mas com potencial para avançar tanto a neurociência quanto a neurorreabilitação.

O uso da ICM, através do EEG com a Estimulação Elétrica Funcional, com o objetivo de estimular os músculos do membro superior durante a fase de execução da OA diante da execução de um ato motor observado, pode apresentar vantagens, como a melhora do desempenho, qualquer que seja a gravidade de seu comprometimento neurológico, como a doença de Parkinson grave (ROSSI et al., 2021).

A literatura apresenta diferentes ICM empregadas a pessoa com DP e, até o momento, não há estudo avaliando o treinamento de imaginação motora e observação da ação para melhora da atividade do córtex sensoriomotor (SM1) com EEG associando a uma luva háptica robótica. Sendo uma ICM não é invasiva, capaz de mostrar um *neurofeedback* rápido e efetivo sobre a ativação da região de interesse, torna-se promissora para melhorar as atividades diária e funcionais do membro superior comprometido.

3 PROBLEMA DE PESQUISA

A imaginação motora e a observação da ação quando aplicadas isoladamente ou combinadas interferem positivamente nas alterações motoras em membros superiores e nas alterações cognitivas na doença de Parkinson?

4 HIPÓTESES

4.1 Hipótese Nula

H0 - Não haverá melhora nos sintomas motores de membros superiores após o tratamento.
H0 – Não haverá melhora nos sintomas cognitivos após o tratamento.
H0 – Não haverá melhora do desempenho e da satisfação na realização das ocupações após o tratamento.

4.2 Hipótese Alternativa

H1 - Haverá melhora nos sintomas motores de membros superiores após o tratamento.
H1 – Haverá melhora nos sintomas cognitivos após o tratamento.
H1 – Haverá melhora do desempenho e da satisfação na realização das ocupações após o tratamento.

5 OBJETIVOS

5.1 Objetivo Geral

Investigar os efeitos da imaginação motora e da observação da ação nas alterações motoras em membros superiores e nas alterações cognitivas na doença de Parkinson.

5.2 Objetivos Específicos

1. Analisar os efeitos isolados da imaginação motora e da observação da ação nas alterações motoras em membros superiores, através do *Test d'Évaluation des Membres Supérieurs of Personnes Âgées* (TEMPA) e do *9-Hole Peg Test* (9HPT) e alterações cognitivas, através do *Parkinson's Disease-Cognitive Rating Scale* (PD-CRS).

2. Analisar os efeitos da combinação da imaginação motora e da observação da ação nas alterações motoras em membros superiores, através do *Test d'Évaluation des Membres Supérieurs of Personnes Âgées* (TEMPA) e do *9-Hole Peg Test* (9HPT) e alterações cognitivas, através do *Parkinson's Disease-Cognitive Rating Scale* (PD-CRS).
3. Comparar os efeitos entre a imaginação motora e execução da ação com a observação da ação e a execução da ação.
4. Comparar os efeitos entre o uso do exoesqueleto combinado com imaginação motora e execução da ação e com observação da ação e execução da ação.
5. Avaliar o reflexo do efeito da imaginação motora e da observação da ação no desempenho ocupacional na realização de atividades relacionadas com as áreas de autocuidado, produtividade e lazer, através da Medida Canadense de Desempenho Ocupacional (COPM).

6 METODOLOGIA DO ESTUDO

6.1 Delineamento

Trata-se de um estudo do tipo ensaio clínico controlado randomizado, simples-cego, uma vez que os participantes serão distribuídos aleatoriamente em grupos para aplicação das intervenções por um período de tempo, analisando posteriormente os desfechos do estudo (KANG; RANG; PARK 2008). O tipo de estudo se justifica pela possibilidade de evidências para a prática clínica.

6.2 Área de Abrangência

Grande área: Ciências da Saúde.
Área: Fisioterapia e Terapia Ocupacional.

6.3 Local da pesquisa

A realização da pesquisa será em dois locais diferentes. Na cidade de Santa Maria a pesquisa ocorrerá nas dependências da Universidade Federal de Santa Maria, no prédio vinculado ao Departamento de Terapia Ocupacional. Na cidade de Porto Alegre a pesquisa ocorrerá na NeuroGold, uma clínica referência na reabilitação funcional de indivíduos com patologias neurológicas.

6.4 População e amostra

A população alvo do estudo envolve pessoas com doença de Parkinson, considerando os critérios de inclusão e exclusão para seleção. A amostragem não probabilística do estudo aponta para o recrutamento por conveniência da população, a partir da divulgação em mídia e contato com secretaria de saúde dos municípios e associações

6.5 Critérios de Elegibilidade

6.5.1 Critérios de Inclusão

Como critérios de inclusão, o participante do estudo tem que:

1) apresentar diagnóstico de doença de Parkinson e estar no estágio 1-3 na escala Hoehn e Yahr, condizentes com incapacidade leve e moderada, com o intuito de homogeneizar a amostra em estágios menos avançados da DP.
2) ter idade entre 20 anos até 59 anos de idade;
3) fazer uso estável de medicamentos;
4) não apresentar comprometimento cognitivo ou demência, tendo pontuação superior a 26 pontos na *Montreal Cognitive Assessment* (MoCA);
5) ser minimamente capaz de imaginar atividades motoras, apresentando uma pontuação mínima de 20 pontos pelo Questionário de Imagens Cinestésicas e Visuais (KVIQ-10) (MALOUIN et al., 2007);
6) apresentar alteração motora no membro superior dominante, verificado com o *9-Hole Peg Test* (9HPT) acima do tempo médio conforme o sexo e a dominância manual, sendo: homens 21.1 segundos para mão dominante e 22.3 segundos para a mão não dominante; para mulheres com DP 19.9 segundos com a mão dominante e 21.4 segundos com a mão não dominante (EARHART et al., 2011);
7) Ter assinado o Termo de Consentimento Livre e Esclarecido.

6.5.2 Critérios de exclusão

Como critérios de exclusão, o participante do estudo:

1) não apresentar distúrbios adicionais do sistema nervoso central ou outras condições que podem afetar a função da extremidade superior e inferior;

2) apresentar outras condições crônicas não controladas, podendo interferir na segurança do participante.

6.6 Instrumentos de avaliação

Os quatro instrumentos mencionados abaixo serão aplicados para os critérios de inclusão dos participantes no estudo.

6.6.1 Escala de Hoehn e Yahr

A Escala de Hoehn e Yahr aborda cinco estágios para avaliar a gravidade da DP. Tendo como vantagem a facilidade de aplicação e a simplicidade, também é utilizada principalmente para definir critérios de inclusão e exclusão (GOETZ et al., 2004). A escala avalia: instabilidade postural, rigidez, tremor e bradicinesia. A classificação 1, 2 e 3 são referentes a incapacidade leve à moderada e nos estágios 4 e 5 são incapacidades mais severas. Para o estudo, serão considerados os estágios 1-3 na escala Hoehn e Yahr para os critérios de inclusão (Anexo 1).

6.6.2 *Montreal Cognitive Assessment*

O *Montreal Cognitive Assessment* (MoCA) é considerado o mais adequado instrumento para triagem de comprometimento cognitivo leve e demência na DP, uma vez que em pessoas com DP é sensível a pequenas alterações cognitivas (VÁZQUEZ et al., 2019). O MoCA é composto por oito domínios cognitivos pontuados de zero até 30 pontos e tem um tempo de aplicação em torno de dez minutos. Os domínios são: habilidades visuais e espaciais, função executiva, linguagem, memória, atenção e orientação, cálculo e abstração (MEMÓRIA et al., 2012) (Anexo 2). O MoCA tem um bom efeito teste-reteste, baixa variabilidade interavaliador, sensibilidade de 82% e especificidade de 75% em relação a outros testes para detecção de comprometimento cognitivo leve e demência usando um corte de 26 pontos.

6.6.3 Questionário de Imagens Cinestésicas e Visuais

O Questionário de Imagens Cinestésicas e Visuais (KVIQ-10) é um teste confiável e válido para identificar a capacidade de imaginação em pessoas com DP (DEMANBORO et al., 2018). É fácil de administrar e os movimentos, tanto reais como imaginários, são apropriados

para indivíduos com neuropatologia (RANDHAWA; HARRIS; BOYD, 2010; HEREMANS et al., 2011). A versão KVIQ-10 se trata de um questionário de 10 itens que avalia a capacidade de imaginação visual (5 itens) e cinestésica (5 itens) (MALOUIN et al., 2007).

Primeiro se descreve o movimento, depois o demonstra e, em seguida, o participante é solicitado a realizar o movimento, imaginá-lo (usando uma perspectiva de primeira pessoa) e, em seguida, classificar a clareza das imagens visuais ou a intensidade das sensações associadas uma imagem de movimento. Os movimentos são: flexão de ombro, oponência do polegar com os outros dedos, flexão de tronco para frente, abdução de quadril, batidas de pé. Utiliza-se uma escala visual variando de 1 (“sem imagem / sensação”) a 5 (“imagem tão clara quanto ver / tão intensa quanto executar a ação”) (Anexo 3).

A pontuação total é de 50 pontos. Quanto ao resultado tem-se que: pontuações mais altas, maior capacidade de imaginação (RANDHAWA; HARRIS; BOYD, 2010; HEREMANS et al., 2011). Assim, o KVIQ-10 será utilizado como instrumento de critério de inclusão, considerando a pontuação mínima de 20 pontos, pois se espera uma capacidade mínima de imaginação para participação no estudo. Durante o teste, será registrado se o lado é dominante ou não dominante.

6.6.4 *9-Hole Peg Test*

O 9-Hole Peg Test (9HPT) envolve uma tarefa de inserção de pinos que permite a correlação com a gravidade e com a duração da DP por avaliar a função da extremidade superior (EARHART et al., 2011). A tarefa exige movimentos dos dedos precisos e coordenados, sendo que o participante deve pegar rapidamente nove pinos pequenos de dentro de um recipiente, um de cada vez, colocá-los em buracos em um tabuleiro e, em seguida, move-los de volta ao recipiente (MATHIOWETZ et al., 1985).

O tempo médio que se considera para concluir o 9HPT varia conforme o estágio da DP (Anexo 4), considerando a escala Hoehn e Yahr, a sendo que para cada estágio tem uma média de tempo para realização da tarefa (EARHART et al., 2011). No entanto, para o estudo, considerando como critérios de inclusão, tem-se o escore de acordo com o sexo e a dominância manual, devendo apresentar um tempo maior do que o indicado, justamente para identificar que exista comprometimento na destreza manual.

Logo, para homens com DP tem-se: 21.1 segundos para mão dominante e 22.3 segundos para a mão não dominante; para mulheres com DP 19.9 segundos com a mão dominante e 21.4 segundos com a mão não dominante (EARHART et al., 2011). Para critério de seleção, o teste

será realizado para verificar se tem comprometimento motor no lado dominante. Além do uso para os critérios de seleção, o 9HPT também será usado no pré e no pós teste no lado dominante.

Os quatro instrumentos abaixo serão usados pré e pós intervenção do estudo para avaliação dos sintomas motores e não motores. Haverá, também, o preenchimento de uma avaliação inicial, elaborada para a própria pesquisa, com informações pessoais dos participantes e sobre a DP dos mesmos (Apêndice 1). Na avaliação inicial consta indicadores da percepção dos sintomas motores no membro superior dominante e da cognição pelos próprios participantes que serão questionados nas três fases de teste.

6.6.5 Escala de Avaliação da Doença de Parkinson Unificada da Sociedade de Distúrbios do Movimento

O instrumento padrão ouro para avaliar a DP é o teste da Escala de Avaliação da Doença de Parkinson Unificada da Sociedade de Distúrbios do Movimento (MDS-UPDRS) que contém quatro partes: 1) aspectos não motores de experiências da vida diária; 2) aspectos motores de experiências da vida diária; 3) avaliação motora; e 4) complicações motoras (GOETZ et al., 2004). Para o estudo, será considerada a terceira parte (UPDRS-III), que envolve a avaliação motora, mas não serão considerados os seguintes itens fala e expressão facial, bem como os itens correspondentes aos sintomas motores em membros inferiores.

Sendo assim, serão considerados os seguintes itens: tremor postural e tremor cinético nas mãos, rigidez, bater os dedos continuamente, movimento das mãos e movimentos rápidos alternados das mãos; além da amplitude do tremor de repouso e da persistência do tremor de repouso para os membros superiores. O que permite avaliar: velocidade, amplitude, hesitações, interrupções e diminuições da amplitude, além do tremor postural e cinético das mãos e amplitude e persistência do tremor em repouso (GOETZ et al., 2004). Para cada um dos oito itens que serão considerados, pode ser atribuído um valor entre 0 à 4 pontos. Para a pontuação máxima possível, quanto maior a pontuação, pior são os sintomas. Como são oito itens totais, tem-se a pontuação total na parte III o MDS-UPDRS 32 pontos (Anexo 5).

6.6.6 *Test D'évaluation Des Membres Supérieurs Des Personnes Âgées*

Considerando a importância de avaliar as limitações dos membros superiores com testes ou tarefas representativas da vida diária, incluindo o uso de objetos, houve a escolha do "*Test*

d'Évaluation des Membres Supérieurs of Personnes Âgées" (TEMPA) (DE FREITAS et al., 2017). O TEMPA envolve tarefas unilaterais e bilaterais e uma variedade de objetos reais para avaliação de parâmetros quantitativos (cotação da velocidade de execução em décimos de segundo) e qualitativos (pontuação funcional e de análise de tarefa).

As tarefas funcionais são oito, sendo quatro tarefas são bilaterais: abrir um pote e e tirar uma colher de café cheia, destravar uma fechadura e abrir um recipiente de comprimidos, escrever em um envelope e colar um selo nele, amarrar um lenço no pescoço, e embaralhar e distribuir cartas de baralho; e quatro tarefas são unilaterais: pegar e carregar uma jarra; pegar uma jarra e despejar água em um copo, manusear moedas e pegar e mover pequenos objetos (NEDELEC et al., 2011).

A pontuação funcional está de acordo com uma escala de quatro níveis: 0, a tarefa foi concluída com sucesso sem hesitação ou dificuldade; 1, alguma dificuldade ou hesitação em completar a tarefa; 2, a tarefa foi parcialmente executada ou certas etapas realizadas com dificuldade significativa; parte da tarefa modificada ou necessidade de assistência; e 3, não conseguiu completar a tarefa, mesmo com assistência. Assim, o valor do escore funcional total representa a soma das tarefas unilaterais direita (0 a 12), esquerda (0 a 12) e tarefas bilaterais (0 a 12) e, portanto, pode variar de 0 a 36 pontos (DE FREITAS et al., 2017).

A análise da tarefa quantifica a dificuldade encontrada de acordo com cinco itens referentes às habilidades sensoriais e motoras do membro superior: força, amplitude de movimento, precisão de movimentos amplos, agarrar e precisão de movimentos finos (NEDELEC et al., 2011). Assim, a soma é realizada para as cinco dimensões da análise de tarefas, variando de 0 a 150 pontos. Lembra-se que a precisão do movimento fino não cotada para tarefas 1-3, e a força não é cotada para tarefas 5-8. O escore total representa a soma da graduação funcional e da análise de tarefas, totalizando 186 pontos (Anexo 6). A avaliação será realizada pré e pós intervenção, sendo que as tarefas unilaterais serão realizadas somente com o membro superior dominante. Para uma melhor interpretação da avaliação, especialmente na análise da tarefa, a mesma será gravada para análise posterior.

6.6.7 Parkinson's Disease-Cognitive Rating Scale

A Escala de Avaliação Cognitiva da Doença de Parkinson (PD-CRS) surge diante da necessidade de uma abordagem mais abrangente para a avaliação cognitivas de funções fronto-subcorticais e corticais prejudicadas ao longo da DP (PAGONABARRAGA et al., 2008). O PD-CRS pode auxiliar na identificação de pessoas com DP com necessidade de mais avaliações

e cuidados específicos, diferenciando quem tem DP com comprometimento cognitivo de quem tem DP e está cognitivamente intacto (ROSCA; SIMU, 2020).

Na PD-CRS são nove funções avaliadas, sendo sete envolvendo a "frontal-subcortical": atenção sustentada, memória operacional, evocação imediata e tardia de memória verbal (lista de palavras), fluência verbal alternada, fluência verbal de ações e desenho do relógio espontâneo; e duas envolvendo a "cortical posterior": nomeação de vinte figuras por confronto visual, somada à cópia do desenho do relógio (Anexo 7).

O tempo de administração é de aproximadamente 20 minutos e é de domínio público, estando o treinamento disponível. O escore total da PD-CRS é de 134 pontos, sendo a pontuação de corte ideal de 81 pontos, e que a pontuação menor ou igual a 64 pontos indica DP com demência (ROSCA; SIMU, 2020).

6.6.8 Medida Canadense de Desempenho Ocupacional

A Medida Canadense de Desempenho Ocupacional (COPM) é uma entrevista semiestruturada que permite a identificação das atividades que são importantes, mas que existe uma dificuldade em realizá-las ou não consegue realizá-las, denominadas de atividades-problema (DEDDING et al., 2004). A COPM considera as áreas do desempenho ocupacional: autocuidado, produtividade e lazer. A pontuação da importância varia de 1 à 10 pontos. As atividades-problema mais importantes (até cinco delas) serão pontuadas também quanto ao desempenho: sendo 1 ponto como “incapaz de fazer” até 10 pontos como “capaz de fazer extremamente bem”; e também quanto à satisfação: sendo 1 ponto para “nada satisfeito” e 10 pontos para “extremamente satisfeito”. Ao final, tem-se uma média do valor do desempenho e da satisfação. Para o estudo, serão considerados os valores da primeira avaliação (antes da intervenção) e da segunda avaliação (depois da intervenção), tendo-se as médias das somas das pontuações pela quantidade de atividades-problema mencionadas (Anexo 8).

6.7 Desfechos

6.7.1 Desfecho Principal

Como desfecho principal do estudo, espera-se para o apontamento de evidência clínica de que as intervenções de observação da ação e de imaginação motora, refletindo positivamente

nos sintomas motores dos membros superiores, especialmente na melhora na pontuação do TEMPA.

6.7.2 Desfecho Secundário

Como desfecho secundário do estudo, espera-me melhora nos sintomas motores da DP através da UPDRS-III, e cognitivos da DP pela Escala de Avaliação Cognitiva da Doença de Parkinson (PD-CRS), e do reflexo das intervenções no desempenho ocupacional, com a melhora do desempenho e da satisfação na realização das atividades diárias através da COPM.

6.8 Procedimento de coleta de dados

6.8.1 Etapas do Estudo

Após a seleção dos participantes, haverá a randomização para alocação dos mesmos nos grupos. A alocação será por meio do programa Randomizer considerando uma atribuição aleatória em blocos para uma chance igual a todos participantes, tendo a atribuição em cada uma das cinco possibilidades da condição experimental, separando-os aleatoriamente em cinco grupos experimentais (GE).

As etapas para coleta de dados do estudo envolverão o pré-teste (T0), as 10 sessões de intervenção, o pós-teste imediato (T1) e o teste após um período de quatro semanas sem intervenção para acompanhamento – *follow-up* (T2). Todos os GE serão avaliados nas três etapas correspondentes aos testes do estudo (T0, T1 e T2). O tempo de aplicação dos testes será em torno de 30 a 45 minutos. O T2 será realizado por meio de contato telefônico para questionar sobre a percepção em relação ao membro superior dominante e a cognição, bem como a reavaliação da COPM em relação ao desempenho e a satisfação.

O estudo é simples-cego, uma vez que a pesquisadora realizará o protocolo de intervenção com os participantes, mas não participará das etapas de testes T0, T1 e T2. Tais etapas serão realizadas por um grupo de participantes do projeto de pesquisa que serão treinados pela pesquisadora para aplicação dos instrumentos.

6.8.2 Proposta de Intervenção

Os GE serão formados de acordo com a intervenção proposta: imaginação motora, observação da ação e execução motora (GE1), imaginação motora e execução da ação (GE2),

observação da ação e execução da ação (GE3), imaginação motora, execução da ação e exoesqueleto (GE4) e observação da ação, execução da ação e exoesqueleto (GE5).

No que envolve as intervenções, haverá a elaboração de um protocolo para que a aplicabilidade ocorra igualmente para todos os participantes, tanto da imaginação motora (IM) quanto da observação da ação (OA), além da execução da ação (EA), bem como do exoesqueleto. A escolha das ações imaginadas, observadas e executadas será a partir das atividades de três áreas do desempenho ocupacional: autocuidado, produtividade e lazer. Assim, a aplicabilidade do protocolo envolverá uma abordagem a partir de ocupações realizadas de acordo com a COPM como: cuidados pessoais, mobilidade independência para autocuidado; trabalho e tarefas domésticas para produtividade; e recreação tranquila, recreação ativa e socialização para lazer. A seleção de tais ocupações torna o protocolo centralizado nas necessidades de cada pessoa a partir do que ela relata que é importante, sendo de uma modalidade mais singularizada.

Para a IM serão gravados áudios descrevendo as ações. Para a OA serão gravados vídeos das ações. A execução da ação será das mesmas atividades imaginadas e observadas. A orientação para a realização da IM, OA e EX é na posição sentada, sendo que para a EA, dependendo da ação a executar, pode ser sentado ou em pé. Durante a IM o participante deverá permanecer com os olhos fechados. As ações para a imaginação serão conduzidas para acontecer em primeira pessoa, em que se imagina realizando a ação (imagem cinestésica) (SILVA et al., 2016). É importante que o ambiente não tenha qualquer tipo de interferência que cause interrupções. Há a possibilidade de aplicabilidade das intervenções em grupo ou individualmente. As intervenções de todos grupos serão de uma abordagem intensivista de 10 sessões contínuas, com intervalo de dois dias na metade da intervenção, totalizando duas semanas, sendo cada sessão de 60 minutos por dia. Cabe ressaltar que, para entendimento de todas as intervenções, será realizado no T0 uma experimentação das mesmas para que não ocorra nenhuma dúvida.

a) imaginação motora, observação da ação e execução da ação (GE1): na posição sentada, o participante assistirá o vídeo gravado para realizar a observação da ação, com duração de 10 minutos. Logo após, fechará os seus olhos, e receberá a descrição da ação, através de áudio gravado da mesma ação anterior, para imaginá-la, com duração de 10 minutos. Na posição sentada ou em pé, conforme a ação a ser realizada, o participante deverá executar a mesma ação imaginada anteriormente, durante o tempo de 10 minutos. Cada sessão de atendimento terá duas ações diferentes, totalizando, assim, os 60 minutos de intervenção.

b) imaginação motora e execução da ação (GE2): na posição sentada, o participante fechará os seus olhos, e receberá a descrição da ação, através de áudio gravado, para imaginá-la, com duração de 10 minutos. Logo, na posição sentada ou em pé, conforme a ação a ser realizada, o participante deverá executar a mesma ação imaginada anteriormente, durante o tempo de 10 minutos. Cada sessão de atendimento terá três ações diferentes, totalizando, assim, os 60 minutos de intervenção.

c) observação da ação e execução da ação (GE3): na posição sentada, o participante assistirá o vídeo gravado para realizar a observação da ação, com duração de 10 minutos. Logo, na posição sentada ou em pé, conforme a ação a ser realizada, o participante deverá executar a mesma ação observada anteriormente, durante o tempo de 10 minutos. Cada sessão de atendimento terá três ações diferentes, totalizando, assim, os 60 minutos de intervenção.

d) imaginação motora, execução da ação e exoesqueleto (GE4): na posição sentada, o participante fechará os seus olhos, e receberá a descrição da ação, através de áudio gravado, para imaginá-la, com duração de 10 minutos. Na posição sentada ou em pé, conforme a ação a ser realizada, o participante deverá executar a mesma ação imaginada anteriormente, durante o tempo de 10 minutos. Em seguida, realizará o protocolo do exoesqueleto, pelo tempo de 40 minutos. Totalizando, assim, os 60 minutos de intervenção.

e) observação da ação, execução da ação e exoesqueleto (GE5): na posição sentada, o participante assistirá o vídeo gravado para realizar a observação da ação, com duração de 10 minutos. Logo, na posição sentada ou em pé, conforme a ação a ser realizada, o participante deverá executar a mesma ação observada anteriormente, durante o tempo de 10 minutos. Em seguida, realizará o protocolo do exoesqueleto, pelo tempo de 40 minutos. Totalizando, assim, os 60 minutos de intervenção.

Como consta nos critérios, os (as) participantes estarão fazendo uso da medicação dopaminérgica normalmente, realizando, assim, as etapas da coleta de dados no período "*on*" da medicação. Logo, os (as) participantes serão convidados a manter também todas as atividades realizadas habitualmente durante o estudo.

6.9 Análise de Dados

Os resultados das variáveis qualitativas serão apresentados através de frequência e percentual, das quantitativas simétricas em média e desvio-padrão e das assimétricas em mediana e amplitude interquartílica [P25 - P75]. A normalidade será verificada pelo teste

Shapiro-Wilk. Os grupos serão comparados quanto às características via teste Qui-Quadrado, ANOVA e/ou teste Kruskal-Wallis com teste de Tukey para comparações múltiplas.

Será utilizada ANOVA mista de medidas repetidas ou, na ausência de normalidade e/ou presença de *missings*, serão utilizados modelos de equações de estimações generalizadas (GEE) para avaliar os efeitos principais de grupo e momento e da interação grupo*momento, com matriz de correlação não estruturada. Serão avaliadas as distribuições que melhor se ajustam aos dados (normal ou gama) pelo critério AIK, com as funções de ligação identidade ou logarítimica, respectivamente. O teste Sidak será utilizado para as comparações múltiplas. Os resultados serão apresentados em média e IC95%. As análises serão realizadas no *software* estatístico SPSS (IBM SPSS *Statistics for Windows, Version* 25.0. Armonk, NY: IBM Corp.). O nível de significância adotado será de 0,05.

6.10 Cálculo Amostral

A amostra foi estimada para encontrar um tamanho de efeito não negligenciável e útil no desfecho principal TEMPA, correspondente a d de Cohen=0,4 (f=0,2), quando aplicada ANOVA, com efeito significativo da interação (momento*intervenção), sugerido por Brysbaert (2019). Considerando duas avaliações, cinco grupos, nível de significância de 0,05 com poder de 80% e adicionados 19% para perdas, serão necessários 95 participantes, sendo 19 por grupo.

6.11 Procedimentos éticos e legais

Com base nas recomendações da Resolução n. 466 do Conselho Nacional de Saúde (BRASIL, 2012), envolvendo pesquisas com seres humanos, o projeto de pesquisa será submetido à apreciação do Comitê de Ética em Pesquisa (CEP-UFCSPA). Os pesquisadores se comprometerão a manter o sigilo e o anonimato de todos os participantes que serão incluídos no estudo. Cada participante da pesquisa receberá o Termo de Consentimento Livre e Esclarecido (TCLE) (Apêndice 2), que informará os propósitos da mesma e riscos associados à sua participação, com linguagem fácil e acessível, além de esclarecer que o estudo é voluntário, podendo ser abandonado a qualquer momento sem lhe cause qualquer prejuízo. A coleta de dados iniciará somente após a aprovação do projeto junto ao CEP.

6.12 Riscos e Benefícios

A pesquisa envolve riscos mínimos aos participantes. O uso dos instrumentos pode causar algum desconforto ou constrangimento ou cansaço durante a aplicabilidade, bem como durante a realização das intervenções. Se acontecer qualquer tipo de situação que cause risco ao participante, o mesmo pode suspender temporariamente a participação na pesquisa, ou desistir da mesma em qualquer etapa, sem provocar-lhe algum prejuízo e serão acompanhados até a resolução.

Como benefícios, a pesquisa pretende investigar a associação dos efeitos da imaginação motora e da observação da ação nas alterações motoras em membros superiores e cognitivas da DP. Caso as hipóteses do estudo aponte para uma possibilidade positiva de ambas as intervenções, tem-se uma condição de melhora de sintomas motores e cognitivos, beneficiando os participantes na condição de saúde. Como reflexo, pode-se melhorar o desempenho ocupacional em relação a realização das atividades diárias. O participante também receberá um parecer dos resultados dos testes, tendo um conhecimento de suas condições motoras e cognitivas.

7 CRONOGRAMA DE ATIVIDADES

**2022**												
**ATIVIDADE / MÊS**	**J**	**F**	**M**	**A**	**M**	**J**	**J**	**A**	**S**	**O**	**N**	**D**
Revisão bibliográfica	X	X	X									
Elaboração do projeto de pesquisa	X	X	X	X	X	X						
Qualificação da Tese							X					
Submissão ao CEP-UFCSPA							X	X				
Treinamento dos (as) colaboradores (as) da pesquisa									X	X	X	X
**2023**												
**ATIVIDADE / MÊS**	**J**	**F**	**M**	**A**	**M**	**J**	**J**	**A**	**S**	**O**	**N**	**D**
Recrutamento dos (as) participantes	X	X										
Coletas de dados	X	X	X	X	X	X	X	X	X	X	X	X
**2024**												
**ATIVIDADE / MÊS**	**J**	**F**	**M**	**A**	**M**	**J**	**J**	**A**	**S**	**O**	**N**	**D**
Análise de dados	X	X										
Desenvolvimento de artigos			X	X	X	X	X	X	X	X	X	X
Entrega do Relatório Parcial									X			
**2025**												
**ATIVIDADE / MÊS**	**J**	**F**	**M**	**A**	**M**	**J**	**J**	**A**	**S**	**O**	**N**	**D**
Elaboração da Tese	X	X	X	X	X							
Defesa da Tese						X						
**2026**												
Entrega do Relatório Final									X			

8 ORÇAMENTO

Os materiais de uso do para realização da pesquisa são de financiamento próprio, sendo as despesas de responsabilidade das pesquisadoras.

ELEMENTO DE DESPESA	QUANTIDADE (unidade)	VALOR (em reais)
MATERIAIS DE CONSUMO		
PAPEL OFÍCIO A 4 BRANCO 75G COM 100 FOLHAS	10	150,00
caneta esferográfica 1.0mm cristal azul	10	10,00
grampeador pequeno de mesa gp1000 26/6 para 20fls	1	12,00
grampo 26/6 galvanizado 5.000 unidades	1	3,00
EQUIPAMENTOS E MATERIAIS PERMANENTES		
Notebook Inspiron 13	1	5.749,00
Equipamento Exoesqueleto	1	10.000,00
TOTAL		15.924,00

REFERÊNCIAS

ABBRUZZESE, Giovanni; AVANZINO, Laura; MARCHESE, Roberta; PELOSIN, Elisa. Action Observation and Motor Imagery: Innovative Cognitive Tools in the Rehabilitation of Parkinson's Disease. **Parkinsons Dis**. v. 2015, n. 124214. 2015. doi: 10.1155/2015/124214.

ABBRUZZESE, Giovanni; MARCHESE, Roberta; AVANZINO, Laura; PELOSIN, Elisa. Rehabilitation for Parkinson's disease: Current outlook and future challenges. **Parkinsonism Relat Disord.** v. 22, supl. 1, p. S60-S64, Jan. 2016. doi: 10.1016/j.parkreldis.2015.09.005.

ABRAHAM, Amit; HART, Ariel; ANDRADE, Isaac; HACKNEY, Madeleine E. Dynamic Neuro-Cognitive Imagery Improves Mental Imagery Ability, Disease Severity, and Motor and Cognitive Functions in People with Parkinson's Disease. **Neural Plast.** v. 2018, 6168507. Mar. 2018. doi: 10.1155/2018/6168507.

ABRAMI, Avner; HEISIG, Stephen; RAMOS, Vesper; THOMAS, Kevin C; HO, Bryan K.; CAGGIANO, Vittorio. Using an unbiased symbolic movement representation to characterize Parkinson's disease states. **Scientific Reports**. v. 10, n. 7377, Apr. 2020. doi: 10.1038/s41598-020-64181-3.

ALIMARDANI, M.; NISHIO, S.; ISHIGURO, H. The importance of visual feedback design in BCIs; from embodiment to motor imagery learning. **PLoS ONE.** v. 11, n. e0161945, 2016.

ALMEIDA, Kelson James; CARVALHO, Larissa Clementino Leite de Sá; MONTEIRO, Tomásia Henrique Oliveira de Holanda; JÚNIOR, Paulo Cesar de Jesus Gonçalves; CAMPOS-SOUSA, Raimundo Nonato. Cut-off points of the Portuguese version of the Montreal Cognitive Assessment for cognitive evaluation in Parkinson’s disease. **Dementia & Neuropsychologia** [online]. v. 13, n. 2, p. 210-215. 2019. doi: 10.1590/1980-57642018dn13-020010.

ANG, K.K.; CHUA, K.S.; PHUA, K.S.; WANG, C.; CHIN, Z.Y.; KUAH, C.W.; LOW, W.; GUAN, C. A randomized controlled trial of EEG-based motor imagery brain-computer interface robotic rehabilitation for stroke. **Clin EEG Neurosci.** v. 46, p. 310–320. 2015.

ANG, K.K.; GUAN, C.; CHUA, K.S.; ANG, B.T.; KUAH, C.W.; WANG, C.; PHUA, K.S.; CHIN, Z.Y.; ZHANG, H. A large clinical study on the ability of stroke patients to use an EEG based moto imagery brain-computer interface. **Clinical EEG and Neuroscience.** v. 42, n. 4, p. 253–258. 2011.

AVANZINO, Laura; PELOSIN, Elisa; MARTINO, Davide; ABBRUZZESE, Giovanni. Motor timing deficits in sequential movements in Parkinson disease are related to action planning: a motor imagery study. **PLoS One.** v. 8, n. 9, Sept. 2013. DOI: 10.1371/journal.pone.0075454.

BANIQUED, P.D.E.; STANYER, E.C.; AWAIS, M.; ALAZMANI, A.; JACKSON, A.E.; MON-WILLIAMS, M.A.; MUSHTAQ, F.; HOLT, R.J. Brain-computer interface

robotics for hand rehabilitation after stroke: a systematic review. **J Neuroeng Rehabil.** v. 23, n. 8(1) 2021. doi: 10.1186/s12984-021-00820-8.

BARBOSA, Alessandra Ferreira; CHEN, Janini; FREITAG, Fernanda; VALENTE, Debora; SOUZA, Carolina de Oliveira; VOOS, Mariana Callil; CHIEN, Hsin Fen. Gait, posture and cognition in Parkinson's disease. **Dement Neuropsychol.** v. 10, n. 4, p. 280-286, Dec. 2016. DOI: 10.1590/S1980-5764-2016DN1004005.

BEK, Judith; GOWEN, Emma; VOGT, Stefan; CRAWFORD, Trevor J.; POLIAKOFF, Ellen. Combined action observation and motor imagery influences hand movement amplitude in Parkinson's disease. **Parkinsonism & Related Disorders**. v. 61, p. 126-131. 2018. DOI: 10.1016/j.parkreldis.2018.11.001.

BEK, Judith; WEBB, Jordan; GOWEN, Emma; VOGT, Stefan; CRAWFORD, Trevor J.; SULLIVAN, Matthew S.; POLIAKOFF, Ellen. Patients' Views on a Combined Action Observation and Motor Imagery Intervention for Parkinson's Disease. **Parkinsons Dis.** v. 2016:7047910. 2016. DOI: 10.1155/2016/7047910.

BEK, Judith; HOLMES, Paul S.; CRAIG, Chesney E.; FRANKLIN, Zoe C.; SULLIVAN, Matthew; WEBB, Jordan, CRAWFORD, Trevor J.; VOGT, Stefan; GOWEN, Emma; POLIAKOFF, Ellen. Action Imagery and Observation in Neurorehabilitation for Parkinson's Disease (ACTION-PD): Development of a User-Informed Home Training Intervention to Improve Functional Hand Movements. **Parkinsons Dis.** v. 23:4559519, Jul. 2021. DOI: 10.1155/2021/4559519.

BIRBAUMER, N.; COHEN, L.G. Brain-computer interfaces: communication and restoration of movement in paralysis. **J Physiol**. v. 579, p. 621–636. 2007.

BONASSI, Gaia; LAGRAVINESE, Giovanna; BISIO, Ambra; RUGGERI, Piero; PELOSIN, Elisa; BOVE, Marco; AVANZINO, Laura. Consolidation and retention of motor skill after motor imagery training. **Neuropsychologia.** v. 143: 107472. 2020 DOI.org/10.1016/j.neuropsychologia.2020.107472.

BRASIL. Ministério da Saúde. Conselho Nacional de Saúde. Resolução n. 466, de 12 de dezembro de 2012.

BRAUN, Susy; BEURSKENS, Anna; KLEYNEN, Melanie; SCHOLS, Jos; WADE, Derick. Rehabilitation with mental practice has similar effects on mobility as rehabilitation with relaxation in people with Parkinson's disease: a multicentre randomised trial. **J Physiother.** v. 57, n. 1, p. 27-34. 2011. DOI: 10.1016/S1836-9553(11)70004-2

BRYSBAERT, M. How Many Participants Do We Have to Include in Properly Powered Experiments? A Tutorial of Power Analysis with Reference Tables. **Journal of Cognition.** v. 2, n. 1:16, p. 1-38. Jul. 2019. DOI: 10.5334/joc.72. PMID: 31517234; PMCID: PMC6640316.

BUCCINO, Giovanni. Action observation treatment: a novel tool in neurorehabilitation. **Philosophical Transactions of the Royal Society**. v. 369(1644). 2014. DOI: 10.1098/rstb.2013.0185

BUCH, E.; WEBER, C.; COHEN, L.G.; et al. Think to move: a neuromagnetic brain-computer interface (BCI) system for chronic stroke. **Stroke**. v. 39, p. 910–917. 2008.

CALIGIORE, Daniele; MUSTILE, Magda; SPALLETTA, Gianfranco; BALDASSARRE, Gianluca. Action observation and motor imagery for rehabilitation in Parkinson's disease: A systematic review and an integrative hypothesis. **Neurosci Biobehav Rev.** v. 72, p. 210-222. 2017. DOI: 10.1016/j.neubiorev.2016.11.005.

CHEN, Y.; YANG, W.; LONG, J.; ZHANG, Y.; FENG, J. et al. Discriminative Analysis of Parkinson’s Disease Based on Whole-Brain Functional Connectivity. PLOS ONE v. 10, n. 4, 2015. DOI: 10.1371/journal.pone.0124153

CIKAJLO, Imre; POTISK, Karmen Peterlin. Advantages of using 3D virtual reality based training in persons with Parkinson's disease: a parallel study. **J Neuroeng Rehabil.** v. 16. Oct. 2019. DOI: 10.1186/s12984-019-0601-1.

DEDDING, Christine; CARDOL, Mieke; EYSSEN, Isaline C.J.M.; DEKKER, Joost; BEELEN, Anita. Validity of the Canadian Occupational Performance Measure: a client-centred outcome measurement. **Clin Rehabil.** v. 18, n. 6, p. 660-667. Sept. 2004. DOI: 10.1191/0269215504cr746oa.

DE FREITAS, Paula Ruiz; LEMOS, Ana Elisa; SANTOS, Mariana Palla; MICHAELSEN, Stella Maris; CORRÊA, Clynton Lourenço; SWAROWSKY, Alessandra. "Test D'évaluation Des Membres Supérieurs Des Personnes Âgées" (TEMPA) to assess upper limb activity in Parkinson's disease. **J Hand Ther.** v. 30, n. 3, p. 320-327, Jul-Sept. 2017. DOI: 10.1016/j.jht.2016.07.003.

DEMANBORO, Alan; STERR, Annette; DOS ANJOS, Sarah Monteiro; CONFORTO, Adriana Bastos. A Brazilian-Portuguese version of the Kinesthetic and Visual Motor Imagery Questionnaire. **Arquivos de Neuro-Psiquiatria.** v. 76, n. 1, p. 26-31. 2018. DOI: 10.1590/0004-282X20170181.

DI RIENZO, Franck; COLLET, Chhristian; HOYEK, Nady; GUILLOT, Aymeric. Impact of neurologic deficits on motor imagery: a systematic review of clinical evaluations. **Neuropsychol Rev.** 2014 Jun;24(2):116-47. DOI: 10.1007/s11065-014-9257-6.

DICKSON, J.M.; GRU¨NEWALD, R.A. Somatic symptom progression in idiopathic Parkinson’s disease. **Parkinsonism Relat Disord.** v. 10, p. 487-492. 2004.

EARHART, Gammon M.; CAVANAUGH, Jim T.; ELLIS, Terry; FORD, Matt P.; FOREMAN, K. Bo; DIBBLE, Lee. The 9-hole PEG test of upper extremity function: average values, test-retest reliability, and factors contributing to performance in people with Parkinson disease. **J Neurol Phys Ther.** v. 35, n. 4, p. 157-163. Dec. 2011. DOI: 10.1097/NPT.0b013e318235da08.

EL-WISHY, Abeer Abo Bakr; FAYEZ, Eman. Effect of Locomotor Imagery Training Added to Physical Therapy Program on Gait Performance in Parkinson Patients: A

Randomized Controlled Studyy. **Egypt J Neurol Psychiat Neurosurg.** v. 50, n. 1, p. 31-37. 2013.

ENDO, Takuyuki; YOKOE, Masaru; FUJIMURA, Harutoshi; SAKODA, Saburo. **Novel methods to evaluate symptoms in Parkinson's disease**—rigidity and finger tapping. In: Diagnostics and Rehabilitation of Parkinson's Disease, edited by J. Dushanova. Rijeka: InTech, 2011:91–206. https://doi.org/10.5772/17967

FERREIRA, Renilson Moraes; ALVES, Wilson Mateus Gomes da Costa; DE LIMA, Tiago Alencar; ALVES, Thiago Goçalves Gibson; ALVES FILHO, Pedro Arthur Madureira; PIMENTEL, Clebson Pantoja; SOUSA, Evitom Correa; CORTINHAS-ALVES, Erik Artur. The effect of resistance training on the anxiety symptoms and quality of life in elderly people with Parkinson's disease: a randomized controlled trial. Arq Neuropsiquiatr. v. 76, n. 8, p. 499-506. Aug. 2018. DOI: 10.1590/0004-282X20180071.

FERREIRA-SÁNCHEZ, María del Rosario; MORENO-VERDÚ, Marcos; CANO-DE-LA-CUERDA, Roberto. Quantitative Measurement of Rigidity in Parkinson´s Disease: A Systematic Review. **Sensors (Basel).** v. 6, n. 20(3):880. Feb. 2020. DOI: 10.3390/s20030880.

FÉRY, Yves-Andre. Differentiating visual and kinesthetic imagery in mental practice. **Can J Exp Psychol**. v. 57, n. 1, p. 1-10. 2003. DOI: 10.1037/h0087408.

FISCHER, Petra; POGOSYAN, Alek; CHEERAN, Binith; GREEN, Alexander L.; AZIZ, Tipu Z.; HYAM, Jonathan. et al. Subthalamic nucleus beta and gamma activity is modulated depending on the level of imagined grip force. **Exp Neurol.** v. 293, p. 53-61. Jul. 2017. DOI: 10.1016/j.expneurol.2017.03.015.

FLORIANO, Eduardo Nascimento; ALVES, Jacqueline Frazão; ALMEIDA, isabela Andrelino; SOUZA, Roger burgo; CHRISTOFOLETTI, Gustavo; SANTOS, Suhaila Mahmoud Smaili. Dual task performance: a comparison between healthy elderly individuals and those with Parkinson's disease. **Fisioterapia em Movimento** [online]. v. 28, n. 2, p. 251-258. 2015. DOI: 10.1590/0103-5150.028.002.AO05.

GATTI, R.; TETTAMANTI, A.; GOUGH, P.M.; RIBOLDI, E.; MARINONI, L.; BUCCINO, G. Action observation versus motor imagery in learning a complex motor task: a short review of literature and a kinematics study. **Neurosci Lett.** v. 12, n. 540, p. 37-42. Apr. 2013. DOI: 10.1016/j.neulet.2012.11.039.

GEBHARDT, A.; VANBELLINGEN, T.; BARONTI, F.; KERSTEN, B.; BOHLHALTER, S. Poor dopaminergic response of impaired dexterity in Parkinson's disease: bradykinesia or limb kinetic apraxia? **Mov Disord.** v. 2, p. 1701-1706. 2008.

GOETZ, Chistopher G.; POEWE, Werner; RASCOL, Olivier; SAMPAIO, Cristina; STEBBINS, Glenn T.; COUNSELL, Carl. et al. Movement Disorder Society Task Force report on the Hoehn and Yahr staging scale: status and recommendations. **Mov Disord.** v. 19, n. 9, p. 1020-1028. Sept. 2004. DOI: 10.1002/mds.20213.

HANSON, T.; FULLER, A.; LEBEDEV, M.; TURNER, D.; NICOLELIS, M. Subcortical Neuronal Ensembles: An Analysis of Motor Task Association, Tremor,

Oscillations, and Synchrony in Human Patients. **Journal of Neuroscience**. v. 32, n. 25, p. 8620-8632. 2012. DOI: 10.1523/JNEUROSCI.0750-12.2012.

RANDHAWA, Bubblepreet Kaur; HARRIS, Susan R.; BOYD, Lara A. The kinesthetic and visual imagery questionnaire is a reliable tool for individuals with Parkinson disease. **Journal of Neurologic Physical Therapy.** v. 34, n. 3, p. 161–167. 2010.

HELDMAN, Dustin A.; JANKOVIC, Joseph; VAILLANCOURT, David E.; PRODOEHL, Janey; ELBLE, Rodgre J.; GIUFFRIDA, Joseph P. Essential tremor quantification during activities of daily living. **Parkinsonism Relat. Disord.** v. 17, n. 7, p. 537–542. 2011. DOI: 10.1016/j.parkreldis.2011.04.017.

HELMICH, Rick C.; BLOEM, Bastiaan R.; TONI, Ivan. Motor imagery evokes increased somatosensory activity in Parkinson's disease patients with tremor. **Hum Brain Mapp.** v. 33, n. 8, p. 1763-1779. 2012. DOI: 10.1002/hbm.21318.

HELMICH, Rick C.; HALLETT, Mark; DEUSCHL, Gunther; TONI, Ivan; BLOEM, Bastiaan R. Cerebral causes and consequences of parkinsonian resting tremor: a tale of two circuits? **Brain.** v. 135, p. 3206-3226. Nov. 2012. DOI: 10.1093/brain/aws023.

HEREMANS, Elke; FEYS, Peter; NIEUWBOER, Alice; VERCRUYSSE, Sarah; VANDENBERGHE, Wim; SHARMA, Nikhil; HELSEN, Werner. Motor imagery ability in patients with early- and mid-stage Parkinson disease. **Neurorehabil Neural Repair.** v. 25, n. 2, p. 168-77. Feb. 2011. DOI: 10.1177/1545968310370750.

HEREMANS, Elke; NIEUWBOER, Alice; FEYS, Peter; VERCRUYSSE, Sarah; VANDENBERGHE, Wim; SHARMA, Nikhil; HELSEN, Werner. External cueing improves motor imagery quality in patients with Parkinson disease. **Neurorehabilitation and Neural Repair.** v. 26, n. 1, p. 27–35. 2012. DOI: 10.1177/1545968311411055.

HU, Zixiang; HAO, Manzhao; XU, Shaoqing; XIAO, Qin; LAN, Ning. Evaluation of tremor interference with control of voluntary reaching movements in patients with Parkinson's disease. **J Neuroeng Rehabil.** v. 16, n. 38. Mar. 2019. DOI: 10.1186/s12984-019-0505-0.

KANEKO, Naotsugu; YOKOYAMA, Hikaru; MASUGI, Yohei; WATANABE, Katsumi; NAKAZAWA, Kimitaka. Phase dependent modulation of cortical activity during action observation and motor imagery of walking: An EEG study. **Neuroimage.** 15;225:117486. Jan. 2017. DOI: 10.1016/j.neuroimage.2020.117486.

KANG, Minsoo; RAGAN, Brian G.; PARK, Jae-Hyeon. Issues in outcomes research: an overview of randomization techniques for clinical trials. **J Athlc Train.** v. 43, 2, p. 215-221. 2008. DOI: 10.4085/1062-6050-43.2.215.

KIKUCHI, A.; BABA, T.; HASEGAWA, T.; SUGENO, N.; KONNO, M.; MIURA, E.; OSHIMA, R.; AOKI, M.; TAKEDA, A. Improvement of Freezing of Gait in Patients with Parkinson's Disease by Imagining Bicycling. **Case Rep Neurol.** v. 6, p. 92-95. 2014. DOI: 10.1159/000362119

KOBAYASHI, Eiji; HIMURO, Nobuaki; MITANI, Yuji; TSUNASHIMA, Takuya; NOMURA, Kyouhei; CHIBA, Susumu. Feasibility and informativeness of the Canadian occupational performance measure for identifying priorities in patients with Parkinson's disease. **Physiotherapy Theory and Practice**. v. 6. Jan. 2022. DOI: 10.1080/09593985.2021.2023926.

LAWRENCE, E.S.; COSHALL, C.; DUNDAS, R. et al. Estimates of the prevalence of acute stroke impairments and disability in a multiethnic population. **Stroke.** v. 32, p. 1279-1284. 2001.

LI, M.; LIU, Y.; WU, Y.; LIU, S.; JIA, J.; ZHANG, L. Neurophysiological substrates of stroke patients with motor imagery-based brain-computer Interface training. **Int J Neurosci.** v. 124, p. 403–415, 2014.

MALLING, Anne Sofie Bogh; MORBERG, Bo Mohr; WERMUTH, Lene; GREDAL, Ole; BECH, Per; JENSEN, Bente Rona. The effect of 8 weeks of treatment with transcranial pulsed electromagnetic fields on hand tremor and inter-hand coherence in persons with Parkinson's disease. **J Neuroeng Rehabil.** v. 31, n. 16(1). Jan. 2019. DOI: 10.1186/s12984-019-0491-2.

MALOUIN, Francine; RICHARDS, Carol L.; JACKSON, Philip L.; LAFLEUR, Martin F.; DURAND, Anne; DOYON, Julien. The Kinesthetic and Visual Imagery Questionnaire (KVIQ) for assessing motor imagery in persons with physical disabilities: a reliability and construct validity study. **J Neurol Phys Ther.** v. 31, n. 1, p. 20-29. Mar. 2007. DOI: 10.1097/01.npt.0000260567.24122.64.

MATHIOWETZ, Virgil; WEBER, Karen; KASHMAN, Nancy; VOLLAND, Gloria. Adult Norms for the Nine Hole Peg Test of Finger Dexterity. **The Occupational Therapy Journal of Research.** v. 5, n. 1, p. 24-38. 1985. DOI: 10.1177/153944928500500102

MEHRHOLZ, J.; POHL, M.; PLATZ, T.; KUGLER, J.; ELSNER, B. Electromechanical and robot-assisted arm training for improving activities of daily living, arm function, and arm muscle strength after stroke. **Cochrane Database Syst Rev.** v. 7, n. 11. 2015. DOI: 10.1002/14651858.CD006876.pub4.

MEMAR, Sara; DELROBAEI, Mehdi; PIETERMAN, Marcus; MCISAAC, Kenneth; JOG, Mandar. Quantification of whole-body bradykinesia in Parkinson's disease participants using multiple inertial sensors. **J Neurol Sci.** v. 15, n. 387, p. 157-165. Apr. 2018. DOI: 10.1016/j.jns.2018.02.001.

MEMÓRIA, Cláudia M.; YASSUDA, Mônica S.; NAKANO, Eduardo Y.; FORLENZA, Orestes V. Brief screening for mild cognitive impairment: validation of the Brazilian version of the Montreal cognitive assessment. **Int J Geriatr Psychiatry**. v. 28, n. 1, p. 34-40. Jan. 2013. doi: 10.1002/gps.3787.

MILLER, K.J.; SCHALK, G.; FETZ, E.E.; DEN, N.I.J.S.M.; OJEMANN, J.G.; RAO, R.P. Cortical activity during motor execution, motor imagery, and imagery-based online feedback. **Proc Natl Acad Sci U S A.** v. 107, 2010.

MYERS, Peter S.; MCNEELY, Marie E.; PICKETT, Kristen A.; DUNCAN, Ryan P.; EARHART, Gammon M. Effects of exercise on gait and motor imagery in people with Parkinson disease and freezing of gait. **Parkinsonism Relat Disord.** v. 53, p. 89-95. 2018. DOI: 10.1016/j.parkreldis.2018.05.006.

MONGE-PEREIRA, E.; IBAÑEZ-PEREDA, J.; ALGUACIL-DIEGO, I.M.; SERRANO, J.I.; SPOTTORNO-RUBIO, M.P.; MOLINA-RUEDA, F. Use of Electroencephalography Brain-Computer Interface Systems as a Rehabilitative Approach for Upper Limb Function After a Stroke: A Systematic Review. **PM R.** v. 9, n. 9, p. 918-932. 2017. DOI: 10.1016/j.pmrj.2017.04.016.

NEDELEC, Bernadette; DION, Karyne; CORREA, José A.; DESROSIERS, Johanne. Upper extremity performance test for the elderly (TEMPA): normative data for young adults. **J Hand Ther.** v. 24, n. 1, p. 31-42. Jany-Mar. 2011. DOI: 10.1016/j.jht.2010.09.001.

NIELSEN, Christina; SIERSMA, Volkert; GHAZIANI, Emma; BEYER, Nina; MAGNUSSON, S. Peter; COUPPÉ, Christian. Health- Related Quality of Life and Physical Function in Individuals with Parkinson's Disease after a Multidisciplinary Rehabilitation Regimen-A Prospective Cohort Feasibility Study**. Int J Environ Res Public Health.** v. 17, n. 20. 2020. DOI: 10.3390/ijerph17207668.

OLDFIELD, R.C. The assessment and analysis of handedness: the Edinburgh inventory. **Neuropsychologia.** v. 9, n. 1, p. 97-113. 1971. DOI: 10.1016/0028-3932(71)90067-4.

PAGONABARRAGA, Javier; KULISEVSKY, Jaime; LLEBARIA, Gisela; GARCÍA-SÁNCHEZ, Carmen; PASCUAL-SEDANO, Berta; GIRONELL, Alexandre. Parkinson's disease-cognitive rating scale: a new cognitive scale specific for Parkinson's disease. **Mov Disord.** v. 15, n. 23(7), p. 998-1005. May. 2008. DOI: 10.1002/mds.22007.

PAUL, Serene S.; DIBBLE, Leland E.; OLIVIER, Genevieve N.; WALTER, Christopher; KEVIN, Duff; SCHAEFER, Sydney Y. Dopamine replacement improves motor learning of an upper extremity task in people with Parkinson disease. **Behav Brain Res.** v. 13. n. 377. Jan. 2020. DOI: 10.1016/j.bbr.2019.112213.

PELOSIN, Elisa; AVANZINO, Laura; BOVE, Marco; STRAMESI, Paola; NIEUWBOER, Alice; ABBRUZZESE, Giovanni. Action observation improves freezing of gait in patients with Parkinson's disease. **Neurorehabil Neural Repair.** v. 24, n. 8, p. 746-52. Oct. 2010. DOI: 10.1177/1545968310368685.

PELOSIN, Elisa; BOVE, Marco; RUGGERI, Piero; AVANZINO, Lauro; ABBRUZZESE, Giovanni. Reduction of bradykinesia of finger movements by a single session of action observation in Parkinson disease. **Neurorehabil Neural Repair.** v. 27, p. 552– 560. 2013. DOI: 10.1177/1545968312471905.

PFURTSCHELLER, G.; LOPES DA SILVA, F.H. Event-related EEG/MEG synchronization and desynchronization: basic principles. **Clin Neurophysiol.** v. 110, p. 1842–57. 1999.

PFURTSCHELLER, G.; NEUPER, C. Future prospects of ERD/ERS in the context of brain computer interface (BCI) developments. **Prog Brain Res**. v.159, p. 433–437. 2006.

PONDÉ, Priscilla de Dio Santos; RODRIGUES, Dayane Nunes; CRISTINA, Layane; BASTOS, Marta Ferreira; SANCHES, Iris Callado; GAMA, Eliane Florencio. Chronic responses of physical and imagery training on Parkinson's disease. **Revista Brasileira de Medicina do Esporte** [online]. v. 25, n. 6, p. 503-508. 2019. DOI: 10.1590/1517-869220192506214238.

POWELL, Douglas; HANSON, Nicholas; THRELKELD, A. Joseph; FANG, Xiang; XIA, Ruiping. Enhancement of parkinsonian rigidity with contralateral hand activation. **Clin Neurophysiol.** v. 122, n. 8, p. 1595-1601. 2011. DOI: 10.1016/j.clinph.2011.01.010.

POWELL, Douglas; THRELKELD, A. Joseph; FANG, Xiang; MUTHUMANI, A.; XIA, Ruiping. Amplitude- and velocity-dependency of rigidity measured at the wrist in Parkinson's disease. **Clin Neurophysiol.** v. 123, n. 4, p. 764-773. 2012. DOI: 10.1016/j.clinph.2011.08.004.

PROUD, E.L.; MORRIS, M.E. Skilled hand dexterity in Parkinson's disease: effects of adding a concurrent task. **Arch Phys Med Rehabil.** v. 91, p. 794-799. 2010.

ROSCA, Elena Cecilia; SIMU, Mihaela. Parkinson's Disease-Cognitive Rating Scale for Evaluating Cognitive Impairment in Parkinson's Disease: A Systematic Review. **Brain Sci.** v. 25. Aug. 2020. DOI: 10.3390/brainsci10090588.

ROSSI, F.; SAVI, F.; PRESTIA, A.; MONGARDI, A.; DEMARCHI, D.; BUCCINO, G. Combining Action Observation Treatment with a Brain-Computer Interface System: Perspectives on Neurorehabilitation. **Sensors (Basel).** v. 21, n. 24, 2021. DOI:10.3390/s21248504

SARASSO, Elisabetta; GEMMA, Mariano; AGOSTA, Federica; FILIPPI, Massimo; GATTI, Roberto. Action observation training to improve motor function recovery: a systematic review. **Arch Physiother.** v. 5, n. 14. 2015. DOI: 10.1186/s40945-015-0013-x

SANTIAGO, Lorena Marques de Melo; DE OLIVEIRA, Daniel Antunes; FERREIRA, Louise Gabriella Lopes de Macêdo; PINTO, Hyanne Yasmin de Brito; SPANIOL, Ana Paula. TRIGUEIRO, Larissa Coutinho de Lucena. et al. Immediate effects of adding mental practice to physical practice on the gait of individuals with Parkinson's disease: Randomized clinical trial. **NeuroRehabilitation.** v. 37, n. 2, p. 263-71. 2015. DOI: 10.3233/NRE-151259.

SHAWEN, Nicholas; O'BRIEN, Megan K.; VENKATESAN, Sanjeev; LONINI, Luca; SIMUNI, Tanya; HAMILTON, Jaime L.; GHAFFARI, Roozbeh; ROGERS, John A.; JAYARAMAN, A. Role of data measurement characteristics in the accurate detection of Parkinson's disease symptoms using wearable sensors. **J Neuroeng Rehabil.** v. 17, n. 52. Apr. 2020. DOI: 10.1186/s12984-020-00684-4.

SHULMAN, Joshua M.; JAGER, Philip L.; FEANY, Mel B. Parkinson’s disease: genetics and pathogenesis. **Annu Ver Pathol MechDis.** v. 6, p. 193-222. 2011. DOI: 10.1146/annurev-pathol-011110-130242.

SILVA, Douglas Monteiro; CORIOLANO, Maria das Graças Wanderley de Sales; MACÊDO, João Gabriel Figuêredo; SILVA, Liliane Pereira; LINS, Otávio Gomes. Practice of mental protocols used in rehabilitation of patients with Parkinson’s disease: a systematic review. **Acta Fisiatr**. v. 23, n. 3, p. 155-160. 2016. DOI: 10.5935/0104-7795.20160030

SOUSA, Nariana Mattos Figueiredo; MACEDO, Roberta Correa; BRUCKI, Sonia Maria Dozzi. Cross-sectional associations between cognition and mobility in Parkinson’s disease. **Dementia & Neuropsychologia** [online]. v. 15, n. 1, p. 105-111. 2021. DOI: 10.1590/1980-57642021dn15-010011.

STURKENBOOM, I.; THIJSSEN, M.; GONS-VAN ELSACKER, J. et al. Guidelines for occupational therapy in Parkinson’s disease rehabilitation. Nijmegen (The Netherlands)/Miami: ParkinsonNet/National Parkinson Foundation. 2011.

TAMIR, Ruth; DICKSTEIN, Ruth; HUBERMAN, Moshe. Integration of motor imagery and physical practice in group treatment applied to subjects with Parkinson’s disease. Neurorehabil Neural Repair. v. 21, n. 1, p. 68-75. 2007. DOI: 10.1177/1545968306292608

VAN DEN NOORT, Josien C.; VERHAGEN, Rens; VAN DIJK, Kess J.; VELTINK, Peter H.; VOS, Michelle C.P.M.; DE BIE, Rob M.A.; BOUR, Lo J.; HEIDA, Ciska T. Quantification of Hand Motor Symptoms in Parkinson's Disease: A Proof-of-Principle Study Using Inertial and Force Sensors. **Ann Biomed Eng.** v. 45, n. 10, p. 2423-2436. Oct. 2017. DOI: 10.1007/s10439-017-1881-x.

VÁSQUEZ, Krisly Arguedas; VALVERDE, Erick Miranda; AGUILAR, Daniel Valerio; GABARAIN, Henro-Jacques Hernádez. Montreal Cognitive Assessment scale in patients with Parkinson Disease with normal scores in the Mini-Mental State Examination. **Dement Neuropsychol.** v. 13, n. 1, p. 78-81. 2013. doi:10.1590/1980-57642018dn13-010008.

WOLPAW, J.R.; BIRBAUMER, N.; MCFARLAND, D.J. PFURTSCHELLER, G.; VAUGHAN, T.M. BRAINCOMPUTER interfaces for communication and control. **Clin Neurophysiol.** v. 113, p. 767–791. 2002.

ZHANG, J.J.Q.; FONG, K.N.K.; WELAGE, N.; LIU, K.P.Y. The activation of the mirror neuron system during action observation and action execution with mirror visual feedback in stroke: a systematic review. **Neural Plast.** v. 2018, p. 1-14. 2018. doi:10.1155/2018/2321045.

ZETTERBERG, H.; FRYKBERG, G.E.; GÄVERTH, J.; LINDBERG, P.G. Neural and nonneural contributions to wrist rigidity in Parkinson's disease: an explorative study using the NeuroFlexor**. Biomed Res Int.** v. 2015. 2015. DOI: 10.1155/2015/276182.

APÊNDICE 1 – AVALIAÇÃO INICIAL

Nome: ____
Data de Nascimento: ______________________ Sexo: M ( ) F ( )
Cidade que reside: ____
Telefone para contato: ____
Escolaridade: ____

Tempo de Diagnóstico: ____
Início dos sintomas (lateralidade): ___________________________________
Doenças Associadas:
____
____

Medicação:
____
____

Horários da medicação DP:
____

Realiza outro tipo de reabilitação: ( ) sim ( ) não
Qual: ____
Frequência: ( ) 1x na semana ( ) 2x na semana ( ) 3x na semana

Realiza atividade física: ( ) sim ( ) não
Qual: ____
Frequência: ( ) 1x na semana ( ) 2x na semana ( ) 3x na semana

Escala de Hoehn e Yahr Modificada: 1( ) 2( ) 3( ) 4( ) 5( )

Mão Dominante: ( ) esquerda ( ) direita

9HPG - tempo de teste (segundos): ________________________

Mão Dominante: ________________

Percepção:

1) Como você considera a gravidade das suas funções motoras do membro superior (destreza, velocidade, precisão do movimento)?

( ) Nenhuma ( ) Pouca ( ) Mediana ( ) Muita ( ) Extrema

2) Como você considera a gravidade das suas funções cognitivas (memória, atenção, concentração, organização)?

( ) Nenhuma ( ) Pouca ( ) Mediana ( ) Muita ( ) Extrema

Avaliador: ____________________________________
Data: _______________

APÊNDICE 2 - TERMO DE CONSENTIMENTO LIVRE ESCLARECIDO

Você está sendo convidado (a) a participar de um estudo coordenado pela professora e pesquisadora Fernanda Cechetti da Universidade Federal de Ciências da Saúde de Porto Alegre (UFCSPA), chamado "**EFEITOS DA IMAGINAÇÃO MOTORA E DA OBSERVAÇÃO DA AÇÃO NAS ALTERAÇÕES MOTORAS EM MEMBROS SUPERIORES E NAS ALTERAÇÕES COGNITIVAS NA DOENÇA DE PARKINSON**". Para quem mora em Santa Maria e região, o estudo será realizado em parceria com a Universidade Federal de Santa Maria (UFSM) e quem mora em Porto Alegre e região, o estudo será realizado em parceria com a Clínica NeuroGold, pela disponibilização de todos os materiais necessários para realização dos testes e das intervenções do tratamento do estudo.

O estudo tem como objetivo investigar os efeitos da imaginação motora e da observação da ação nas alterações motoras em membros superiores e nas alterações cognitivas na doença de Parkinson. O estudo se faz necessário por que a doença de Parkinson é bastante comum em adultos e idosos e não tem cura, causando limitações na realização das atividades diárias, pois a pessoa se sente mais lenta e com dificuldade de se movimentar, além de ter problemas com a memória, como esquecimentos, e ficar desatenta para realizar suas atividades.

Para tanto, serão realizadas três avaliações motoras: alguns itens da terceira parte da Escala de Avaliação da Doença de Parkinson Unificada da Sociedade de Distúrbios do Movimento (UPDRS-III) que são apenas observados a realização de alguns movimentos; o *Test d'Évaluation des Membres Supérieurs of Personnes Âgées* (TEMPA) que envolve a realização de tarefas com uma mão e com as duas mãos e o *9-Hole Peg Test* (9HPT) que consiste apenas na tarefa de pegar e inserir pinos; e uma avalição cognitiva através do *Parkinson's Disease-Cognitive Rating Scale* (PD-CRS), que são itens de repetir palavras, nomear figuras, dizer palavras e fazer desenhos. Vamos perguntar também sobre as atividades que são importantes para você e que não está conseguindo realizar ou com dificuldade em realizar, e você pontuará sobre a forma como as realiza e sobre a satisfação, com a Medida Canadense de Desempenho Ocupacional (COPM). E, também, sobre como você acha que está a gravidade dos sintomas na função do membro superior e das funções cognitivas (memória, atenção, concentração e outras). O tempo de aplicação dos testes será em torno de 30 a 45 minutos.

Após você fazer os testes acima, que serão realizados no início e no final do tratamento, você será solicitado a realizar um tratamento com duração de 10 dias ao total, sendo cinco dias de segunda-feira à sexta-feira, tendo sábado e domingo de intervalo, e depois cinco dias de segunda-feira à sexta-feira. Cada dia de tratamento tem duração de 60 minutos. Após realizar o tratamento de 10 dias, você será avaliado novamente, mas por contato telefônico, em que vamos perguntar somente sobre seu desempenho e sua satisfação na realização das atividades diárias e a parte da percepção que é como você acha que está a gravidade dos sintomas. Depois de quatro semanas do fim do tratamento, vamos marcar um retorno para realização dos testes novamente.

O estudo tem cinco grupos diferentes de tratamento e você ficará em um deles apenas. Vamos explicar cada um deles.

No grupo 1: estando sentado, você assistirá um vídeo gravado para observar uma atividade comum do dia-a-dia, como lavar uma louça, durante 2 minutos. Logo após, com os seus olhos fechados, escutará um áudio gravado descrevendo a mesma atividade anterior, e você deverá imaginar aquela atividade durante 2 minutos. Depois você realizará atividade imaginada e observada. Cada encontro de atendimento terá cinco ações diferentes que serão repetidas duas vezes. Totalizando, assim, os 60 minutos de intervenção.

No grupo 2: estando sentado, você fechará os seus olhos, escutará um áudio gravado descrevendo uma atividade comum do dia-a-dia, como lavar uma louça, e você deverá imaginar aquela atividade durante 2 minutos. Logo em seguida, na posição sentada ou em pé, conforme a atividade a ser realizada, você deverá realizar a mesma atividade que imaginou antes. Cada encontro de atendimento terá cinco ações diferentes que serão repetidas duas vezes. Totalizando, assim, os 40 minutos de intervenção.

No grupo 3: estando sentado, você assistirá um vídeo gravado para observar uma atividade comum do dia-a-dia, como lavar uma louça, durante 2 minutos. Logo em seguida, na posição sentada ou em pé, conforme a atividade a ser realizada, você deverá realizar a mesma atividade que você assistiu no vídeo. Cada encontro de atendimento terá cinco ações diferentes que serão repetidas duas vezes. Totalizando, assim, os 40 minutos de intervenção.

No grupo 4: estando sentado, você fechará os seus olhos, escutará um áudio gravado descrevendo uma atividade comum do dia-a-dia, como lavar uma louça, e deverá imaginar aquela atividade durante 2 minutos. Logo em seguida, na posição sentada ou em pé, conforme a atividade a ser realizada, você deverá realizar a mesma atividade que

imaginou antes. Em seguida, realizará o tratamento com o exoesqueleto, que consiste na colocação de uma touca na cabeça e uma luva robótica na mão que você tem mais dificuldade, você receberá as instruções para imaginar as mesmas atividades, e ao imaginar, a luva irá movimentar seus dedos. O tempo é de 40 minutos. Totalizando, assim, os 60 minutos de intervenção.

No grupo 5: estando sentado, você assistirá um vídeo gravado para observar uma atividade comum do dia-a-dia, como lavar uma louça, durante 2 minutos. Logo em seguida, na posição sentada ou em pé, conforme a atividade a ser realizada, você deverá realizar a mesma atividade que você assistiu no vídeo, durante o tempo de 2 minutos. Em seguida, realizará o tratamento com o exoesqueleto, que consiste na colocação de uma touca na cabeça e uma luva robótica na mão que você tem mais dificuldade, você receberá as instruções para imaginar as mesmas atividades, e ao imaginar, a luva irá movimentar seus dedos. O tempo é de 40 minutos. Totalizando, assim, os 60 minutos de intervenção.

A pesquisa pode envolver riscos mínimos, e as pesquisadoras estão cientes, além de aptas a conduzir a pesquisa de forma segura e oferecer auxílio se necessário, sendo totalmente responsáveis por qualquer situação envolvendo a pesquisa. O uso dos instrumentos pode causar algum desconforto ou constrangimento ou cansaço durante a aplicabilidade, bem como durante a realização das intervenções. Se acontecer qualquer tipo de situação que cause risco, você pode suspender temporariamente a participação na pesquisa, ou desistir em qualquer etapa, sem causar algum prejuízo e será acompanhado até a resolução da situação, sendo garantia a indenização diante de eventuais danos decorrentes da pesquisa. Como benefícios, apostamos em uma possibilidade positiva de tratamento, com melhora da realização dos movimentos do braço e da mão, de lembrar das coisas e estar mais atento, melhorando a maneira que realiza suas atividades diárias. Ao final da pesquisa, você também receberá um parecer dos resultados dos testes, tendo um conhecimento de suas condições motoras e cognitivas.

Os resultados obtidos poderão ser publicados em revistas científicas da área do estudo, ficando garantida a confidencialidade, sigilo e privacidade da sua identidade. Portanto, fica claro, que você tem a liberdade em recusar-se a participar ou retirar seu consentimento, em qualquer fase da pesquisa sem penalidade alguma ou prejuízo pessoal. Qualquer gasto que você tiver com transporte ou alimentação serão realizados pelas pesquisadoras, mas não receberão pagamento por participar da pesquisa.

O presente documento será emitido em duas vias, uma destinada a você e a outra às pesquisadoras. Caso surjam problemas ou dúvidas sobre o estudo, você pode entrar em

contato com as pesquisadoras através dos telefones disponibilizados no final do documento.

Eu, ______________________________________ fui informado(a), de maneira clara e detalhada, quanto aos objetivos da pesquisa acima citada. Recebi informações a respeito da avaliação e das intervenções que serão realizadas e esclareci minhas dúvidas. Sei que em qualquer momento poderei solicitar novas informações e modificar minha decisão de participação do estudo, se assim eu desejar. As responsáveis, Fernanda Cechetti, Kátine Marchezan Estivalet, certificaram-me de que todos os dados da pesquisa serão confidenciais e terei liberdade de retirar a participação nesta pesquisa, caso julgue necessário.

Em caso de dúvidas e/ou reclamações sobre assuntos relacionados com a pesquisa, favor contatar os pesquisadores nos telefones: Fernanda Cechetti (51) 9 8230 7733; Kátine Marchezan Estivalet (55) 9 9936 9852. Ou ainda, pode-se contatar o Comitê de Ética Pesquisa com Seres Humanos da Universidade Federal das Ciências da Saúde de Porto Alegre (51-33038804), localizado na Rua Sarmento Leite, 245, Porto Alegre-RS. O endereço da pesquisadora fica à disposição: Rua Sarmento Leite, 245, Porto Alegre-RS, sala 300B, fone: 51-33038876.

Porto Alegre, de de 202___.

_________________________ _______________________________

Pesquisador Responsável Participante

DECLARAÇÃO DE RESPONSABILIDADE DOS REALIZADORES DA PESQUISA

Expliquei o objetivo, os riscos e benefícios e a natureza da pesquisa. Esclareci todas as dúvidas dos participantes da pesquisa. O participante compreendeu e aceitou participar da pesquisa.

ASSINATURA DA PROFESSORA RESPONSÁVEL:

____

Fernanda Cechetti

Docente UFCSPA

ANEXO 1 – ESTÁGIOS DA DOENÇA DE PARKINSON CONFORME HOEHN E YAHR

ESTÁGIOS	DESCRIÇÃO
0	Nenhum sinal da doença.
1	Doença unilateral.
1,5	Envolvimento unilateral e axial.
2	Doença bilateral sem déficit de equilíbrio (recupera o equilíbrio dando três passos para trás ou menos).
2,5	Doença bilateral leve, com recuperação na estabilização postural.
3	Doença bilateral leve a moderada; alguma instabilidade postural; capacidade para viver independente.
4	Incapacidade grave, ainda capaz de caminhar ou permanecer de pé sem ajuda.
5	Confinado à cama ou cadeira de rodas a não ser que receba ajuda.

Fonte: Goetz e colaboradores (2004).

ANEXO 2 – MONTREAL COGNITIVE ASSESSMENT

MONTREAL COGNITIVE ASSESSMENT (MOCA)
Versão Experimental Brasileira

Nome:________________ Data de nascimento: __/__/__
Escolaridade:________________ Data de avaliação: __/__/__
Sexo:________________ Idade:_____

VISUOESPACIAL / EXECUTIVA

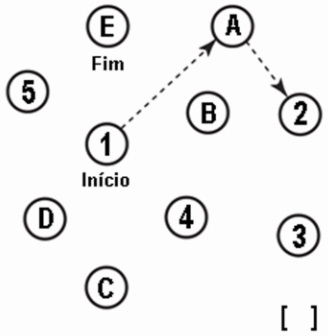

[ ]

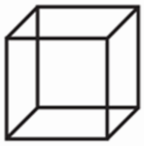

Copiar o cubo [ ]

Desenhar um RELÓGIO (onze horas e dez minutos) (3 pontos)

[ ] Contorno [ ] Números [ ] Ponteiros

Pontos: ___/5

NOMEAÇÃO

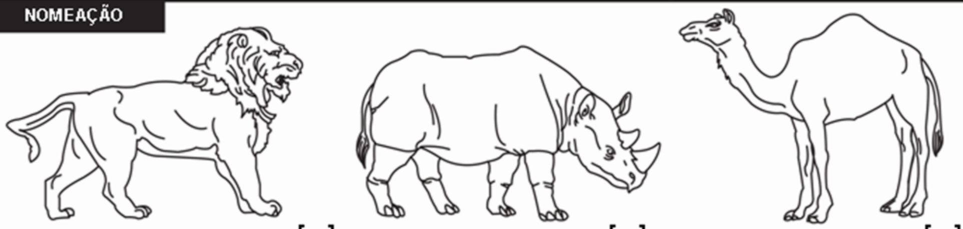

[ ] [ ] [ ]

___/3

MEMÓRIA		Rosto	Veludo	Igreja	Margarida	Vermelho	
Leia a lista de palavras, O sujeito de repeti-la, faça duas tentativas Evocar após 5 minutos	1ª tentativa						Sem Pontuação
	2ª tentativa						

ATENÇÃO Leia a seqüência de números (1 número por segundo)
O sujeito deve repetir a seqüência em ordem direta [ ] 2 1 8 5 4
O sujeito deve repetir a seqüência em ordem indireta [ ] 7 4 2 — ___/2

Leia a série de letras. O sujeito deve bater com a mão (na mesa) cada vez que ouvir a letra "A". Não se atribuem pontos se ≥ 2 erros.
[ ] F B A C M N A A J K L B A F A K D E A A A J A M O F A A B — ___/1

Subtração de 7 começando pelo 100 [ ] 93 [ ] 86 [ ] 79 [ ] 72 [ ] 65
4 ou 5 subtrações corretas: 3 pontos; 2 ou 3 corretas 2 pontos; 1 correta 1 ponto; 0 correta 0 ponto — ___/3

LINGUAGEM Repetir: Eu somente sei que é João quem será ajudado hoje. [ ] O gato sempre se esconde embaixo do Sofá quando o cachorro está na sala. [ ] — ___/2

Fluência verbal: dizer o maior número possível de palavras que comecem pela letra F (1 minuto). [ ]_____ (N ≥ 11 palavras) — ___/1

ABSTRAÇÃO Semelhança p. ex. entre banana e laranja = fruta [ ] trem - bicicleta [ ] relógio - régua — ___/2

EVOCAÇÃO TARDIA	Deve recordar as palavras SEM PISTAS	Rosto	Veludo	Igreja	Margarida	Vermelho	Pontuação apenas para evocação SEM PISTAS	___/5
		[ ]	[ ]	[ ]	[ ]	[ ]		
OPCIONAL	Pista de categoria							
	Pista de múltipla escolha							

ORIENTAÇÃO [ ] Dia do mês [ ] Mês [ ] Ano [ ] Dia da semana [ ] Lugar [ ] Cidade — ___/6

TOTAL ___/30
Adicionar 1 pt se ≤ 12 anos de escolaridade

© Z. Nasreddine MD www.mocatest.org
Versão experimental Brasileira: *Ana Luisa Rosas Sarmento*
Paulo Henrique Ferreira Bertolucci - José Roberto Wajman

(UNIFESP-SP 2007)

ANEXO 3 – QUESTIONÁRIO DE IMAGINAÇÃO CINESTÉSICAS E VISUAL

MOVIMENTOS	Flexão de Ombro	Oponência dos Dedos	Flexão anterior do Tronco	Abdução de Quadril	Bater os Pés
VISUAL					
5 = Imagem clara como visão (imagem tão nítida como vendo)					
4 = Imagem clara					
3 = Imagem moderadamente clara					
2 = Imagem borrada (embaçada)					
1 = Sem imagem					
CINESTÉSICA					
5 = Intensa como uma ação (tão intenso como executando a ação)					
4 = Intensa					
3 = Moderadamente intensa					
2 = Levemente intensa					
1 = Sem sensação					

Fonte: Malouin e colaboradores (2007).

ANEXO 4 - TEMPO MÉDIO DE REALIZAÇÃO DA TAREFA CONFORME OS ESTÁGIOS DA DOENÇA DE PARKINSON

Hoehn e Yahr (estágios)	9-Hole Peg Test (em segundos)	
	mão dominante média ± DP	mão não dominante média ± DP
1	23.5 ± 5.6	23.5 ± 5.2
1.5	23.4 ± 3.2	31.2 ± 10.1
2	26.6 ± 6.6	27.5 ± 6.4
2.5	34.3 ± 22.5	34.4 ± 12.9
3	36.7 ± 16.4	36.8 ± 13.4

Fonte: Earhart e colaboradores (2011).

ANEXO 5 - AVALIAÇÃO DA DOENÇA DE PARKINSON UNIFICADA DA SOCIEDADE DE DISTÚRBIOS DO MOVIMENTO

Parte III: Avaliação Motora

Visão Geral: avaliação dos sinais motores da DP.

Na parte superior do formulário, marque se o participante está utilizando medicação para o tratamento de sintomas da doença de Parkinson e, se estiver sob o uso de levodopa, o tempo desde a última dose.

Se o participante recebe tratamento para os sintomas da doença de Parkinson, marque também o estado clínico usando as seguintes definições:

ON é estado funcional típico de quando recebem medicação e têm uma boa resposta.

OFF é o estado funcional típico de quando os têm uma má resposta apesar de tomarem medicação.

Todos os itens devem ser pontuados com um valor inteiro (sem meios pontos, sem dados em falta). Instruções específicas são fornecidas para testar cada item. O avaliador demonstra enquanto descreve a tarefa que o participante deve realizar e pontua a função imediatamente depois. Para os itens Espontaneidade Global de Movimento e Tremor de Repouso, a pontuação será obtida durante toda a avaliação. No final da pontuação, indicar se discinesia (coreia ou distonia) esteve presente no momento da avaliação, e se assim for, se estes movimentos interferiram com a avaliação motora.

3a O participante usa medicação para o tratamento dos sintomas da doença de Parkinson?

Não (   )   Sim (   )

3b Se o participante recebe medicação para o tratamento dos sintomas da doença de Parkinson, marque o estado clínico do participante usando as seguintes definições:

ON: estado funcional típico de quando os participantes estão a tomar medicação e têm uma boa resposta.

OFF: estado funcional típico de quando os participantes têm uma resposta fraca apesar de tomarem medicação.

3c O participante usa Levodopa ?

Não (   )   Sim (   )

3.c1 Se sim, minutos desde a última dose de levodopa: ______________

RIGIDEZ

Instruções para o avaliador: A rigidez é avaliada usando movimentos passivos lentos das grandes articulações com o participante numa posição relaxada e o avaliador manipulando os membros. Primeiro teste sem a manobra de ativação. Teste e pontue cada membro separadamente. Para os braços, teste as articulações do punho e cotovelos simultaneamente. Se não for detectada rigidez, use uma manobra de ativação tais como bater o primeiro e o segundo dedo, abrir/fechar a mão. Explique ao participante que deve tentar relaxar o máximo possível enquanto é testada a rigidez.

0: Normal: Sem rigidez.

1: Discreto: Rigidez apenas detectada com uma manobra de ativação.

2: Ligeiro: Rigidez detectada sem a manobra de ativação, mas a amplitude total de movimento é facilmente alcançada.

3: Moderado: Rigidez detectada sem a manobra de ativação; amplitude total alcançada com esforço.

4: Grave: Rigidez detectada sem a manobra de ativação e amplitude total de movimento não alcançada.

(   ) MSE   (   ) MSD

BATER DOS DEDOS DA MÃO (PINÇA)

Instruções para o avaliador: Cada mão é testada separadamente. Faça a demonstração da tarefa, mas não realize a tarefa enquanto o participante é testado. Instrua o participante para que toque com o indicador no polegar 10 vezes, o mais rápido e amplo possível. Pontue cada lado separadamente, avaliando velocidade, amplitude, hesitações, interrupções e diminuição da amplitude.

0: Normal: Sem problemas.

1: Discreto: Qualquer dos seguintes: a) o ritmo regular é interrompido com uma ou duas interrupções ou hesitações nos movimentos; b) lentidão mínima; c) a amplitude diminui perto do fim das 10 repetições.

2: Ligeiro: Qualquer um dos seguintes: a) 3 a 5 interrupções durante os movimentos; b) lentidão ligeira; c) a amplitude diminui no meio da sequência das 10 repetições

3: Moderado: Qualquer um dos seguintes: a) mais de 5 interrupções durante os movimentos ou pelo menos uma pausa mais longa (*bloqueio*); b) lentidão moderada; c) a amplitude diminui após o primeiro movimento.

4: Grave: Não consegue ou quase não consegue executar a tarefa devido à lentidão, interrupções ou decrementos.

(   ) MSE   (   ) MSD

MOVIMENTOS DAS MÃOS

Instruções para o avaliador: Cada mão é testada separadamente. Faça a demonstração da tarefa, mas não realize a tarefa enquanto o participante é testado. Instrua o participante a fechar a mão com força com o braço fletido ao nível do cotovelo de forma que a palma da mão esteja virada para o avaliador. Peça ao participante para abrir a mão 10 vezes o mais rápido e amplo possível. Se o participante não fechar a mão firmemente ou não abrir a mão por completo, lembre-o de o fazer. Pontue cada lado separadamente, avaliando velocidade, amplitude, hesitações, interrupções e diminuições da amplitude.

0: Normal: Sem problemas.

1: Discreto: Qualquer dos seguintes: a) o ritmo regular é interrompido com uma ou duas interrupções ou hesitações dos movimentos; b) lentidão mínima; c) a amplitude diminui perto do fim da tarefa.

2: Ligeiro: Qualquer dos seguintes: a) 3 a 5 interrupções durante o movimento; b) lentidão ligeira; c) a amplitude diminui no meio da tarefa.

3: Moderado: Qualquer dos seguintes: a) mais de 5 interrupções durante o movimento ou pelo menos uma pausa mais prolongada (*bloqueio*); b) lentidão moderada; c) a amplitude diminui após a primeira sequência de abrir e fechar.

4: Grave: Não consegue ou quase não consegue executar a tarefa devido à lentidão, interrupções ou decrementos.

(   ) MSE   (   ) MSD

MOVIMENTOS DE PRONAÇÃO-SUPINAÇÃO DAS MÃOS

Instruções para o avaliador: Cada mão é testada separadamente. Faça a demonstração da tarefa, mas não realize a tarefa enquanto o participante é testado. Instrua o participante a estender o braço em frente ao seu corpo com a palma da mão virada para baixo; depois a virar a palma da mão para cima e para baixo alternadamente 10 vezes o mais rápido e amplo possível. Pontue cada lado separadamente, avaliando velocidade, amplitude, hesitações, interrupções e diminuições da amplitude.

0: Normal: Sem problemas.

1: Discreto: Qualquer dos seguintes: a) o ritmo regular é interrompido com uma ou duas interrupções ou hesitações dos movimentos; b) lentidão mínima; c) a amplitude diminui perto do fim da sequência.
2: Ligeiro: Qualquer dos seguintes: a) 3 a 5 interrupções durante o movimento; b) lentidão ligeira; c) a amplitude diminui no meio da sequência.
3: Moderado: Qualquer dos seguintes: a) mais de 5 interrupções durante o movimento ou pelo menos uma pausa mais prolongada (*bloqueio*); b) lentidão moderada; c) a amplitude diminui após a primeira sequência de pronação-supinação.
4: Grave: Não consegue ou quase não consegue executar a tarefa devido à lentidão, interrupções ou decrementos.
( ) MSE ( ) MSD

TREMOR POSTURAL DAS MÃOS

Instruções para o avaliador: Todo o tremor, incluindo o tremor de repouso reemergente, que está presente na postura é incluído nesta pontuação. Pontue cada mão separadamente. Pontue a maior amplitude observada. Instrua o participante a estender os braços em frente do corpo com as palmas das mãos viradas para baixo. O punho deve estar reto e os dedos confortavelmente separados para que não se toquem. Observe esta postura durante 10 segundos.
0: Normal: Sem tremor.
1: Discreto: O tremor está presente mas tem menos de 1 cm de amplitude.
2: Ligeiro: O tremor tem pelo menos 1 cm mas menos de 3 cm de amplitude.
3: Moderado: O tremor tem pelo menos 3 cm, mas menos de 10 cm de amplitude.
4: Grave: O tremor tem pelo menos 10 cm de amplitude.
( ) MSE ( ) MSD

TREMOR CINÉTICO DAS MÃOS

Instruções para o avaliador: Este tremor é testado através da manobra de dedo-nariz. Iniciando com o braço estendido, peça ao participante que execute pelo menos três manobras dedo nariz com cada mão, chegando o mais longe possível para tocar o dedo do avaliador. A manobra dedo-ao-nariz deve ser executada com lentidão suficiente para que o tremor não seja ocultado o que pode acontecer com movimentos muito rápidos do braço. Repetir com a outra mão, pontuando cada mão separadamente. O tremor pode estar

presente durante o movimento ou quando se alcança qualquer um dos alvos (nariz ou dedo). Pontue a maior amplitude observada.

0: Normal: Sem tremor.

1: Discreto: O tremor está presente mas tem menos de 1 cm de amplitude.

2: Ligeiro: O tremor tem pelo menos 1 cm mas menos de 3 cm de amplitude.

3: Moderado: O tremor tem pelo menos 3 cm mas menos de 10 cm de amplitude.

4: Grave: O tremor tem pelo pelo menos 10 cm de amplitude.

( ) MSE ( ) MSD

AMPLITUDE DO TREMOR DE REPOUSO

Instruções para o avaliador: Este e o próximo item foram colocados deliberadamente no final da avaliação para permitir ao avaliador reunir observações sobre o tremor de repouso que podem ter surgido a qualquer momento da avaliação, incluindo quando o participante está calmamente sentado, durante a marcha e durante as atividades em que algumas partes do corpo estão em movimento, mas outras estão em repouso. Pontue a amplitude máxima observada em qualquer momento, como a pontuação final. Pontue apenas a amplitude e não a persistência ou a intermitência do tremor. Como parte desta pontuação, o participante deve sentar-se calmamente numa cadeira, com as mãos colocadas nos braços da cadeira (e não no colo) e os pés confortavelmente apoiados no chão durante 10 segundos sem nenhuma outra instrução. O tremor de repouso é avaliado separadamente, mas será considerado para os membros superiores. Pontue apenas a amplitude máxima observada a qualquer momento, sendo essa a pontuação final.

Extremidades

0: Normal: Sem tremor.

1: Discreto.: < 1 cm de amplitude máxima.

2: Ligeiro: = 1 cm mas < 3 cm de amplitude máxima.

3: Moderado: = 3 cm mas < 10 cm de amplitude máxima.

4: Grave: = 10 cm de amplitude máxima.

( ) MSE ( ) MSD

PERSISTÊNCIA DO TREMOR DE REPOUSO

Instruções para o avaliador: Este item recebe uma pontuação única para todo o tremor de repouso foca-se na persistência do tremor de repouso durante o período de avaliação quando diferentes partes do corpo estão em repouso. Este item é pontuado

deliberadamente no final da avaliação para que vários minutos de informação possam ser reunidos em uma única pontuação.

0: Normal: Sem tremor.

1: Discreto: Tremor de repouso presente durante = 25% do tempo de avaliação.

2: Ligeiro: Tremor de repouso presente durante 26-50% do tempo de avaliação.

3: Moderado: Tremor de repouso presente durante 51-75% do tempo de avaliação.

4: Grave: Tremor de repouso presente durante > 75% do tempo de avaliação.

( ) MSE ( ) MSD

IMPACTO DAS DISCINESIAS NAS PONTUAÇÕES DA PARTE III

A. Estiveram presentes discinesias (coreia ou distonia) durante a avaliação?

Não ( ) Sim ( )

B. Se sim, estes movimentos interferiram com as suas pontuações?

Não ( ) Sim ( )

ANEXO 6 - TEST D'ÉVALUATION DES MEMBRES SUPÉRIERS DE PERSONNES AGÉES

TEMPA – versão brasileira

**Nome:**			**Idade:**
**Diagnóstico:**	**Dominância:**	**Data:**	**Amplitude de movimento Passiva:**

**Alteração Visual:** ( ) Sim ( ) Não	**Uso de óculos:** ( ) Sim ( ) Não
**Prejuízo perceptual ou cognitivo:** ( ) Sim ( ) Não	Não avaliado ( )

	**Análise das Tarefas**														
	Velocidade de execução		Graduação Funcional		Amplitude ativa de movimento		Força		Precisão movimentos amplos		Preensão		Precisão movimentos finos		Observações / Comentários
Tarefas	D	E	D	E	D	E	D	E	D	E	D	E	D	E	
1. Pegar e transportar um pote													xxxx	xxxxx	
2. Abrir um pote, tirar uma colher cheia de café															
3. Pegar uma jarra e servir água em um copo													xxxxxxxxxxxxx		
4. Destrancar fechadura e abrir um recipiente contendo pílulas															
5. Escrever em um envelope e colar um selo							xxxxxxxxx								
6. Embaralhar e distribuir cartas de jogo							xxxxxxxxx								
7. Manusear moedas							xxxxxxxxx								
8.Pegar e mover pequenos objetos							xxxxxxxxx								
															Análise Total das Tarefas
Escore Total Tarefas Unilaterais															
Escore Total Tarefas Bilaterais															
Escore Total Combinado															

	**Direito**	**Esquerdo**
**Força de Preensão (Kg)**		
**Resistência (seg)**		

ANEXO 7 – PARKINSON'S DISEASE-COGNITIVE RATING SCALE

1. MEMÓRIA VERBAL COM EVOCAÇÃO LIVRE IMEDIATA

INSTRUÇÕES

Leia para o sujeito, em voz alta, as palavras na lista abaixo (ritmo de uma palavra por segundo). Três tentativas são realizadas, e pede-se ao sujeito que se lembre do maior número possível de palavras após cada tentativa. Leia para o sujeito: **"Eu vou ler uma lista de palavras e, quando finalizar, quero que você repita o maior número de palavras que puder se recordar. Não precisa ser na mesma ordem da minha leitura. Alguma dúvida? Podemos começar?".** (Leia a primeira lista, no ritmo de uma palavra por segundo. Ao concluir, aguarde as respostas do sujeito. Ao terminar, continue com a instrução da 2a tentativa).

	PONTOS		PONTOS
LUZ	0 1	QUADRO	0 1
SEDA	0 1	BICICLETA	0 1
AREIA	0 1	ESTRELA	0 1
CÍLIO	0 1	LEÃO	0 1
ARROZ	0 1	ANEL	0 1
GRAVATA	0 1	PERFUME	0 1

PONTUAÇÃO (1a. TENTATIVA): ____/12

"Agora vou ler as mesmas palavras e quero que se lembre da maior quantidade que puder se lembrar, inclusive as palavras que já foram ditas na primeira tentativa. Não importa a ordem das palavras, procure dizer todas elas incluindo aquelas que já foram recordadas na vez anterior. Pronto?". (Leia as palavras da 2a tentativa, no ritmo de uma palavra por segundo e aguarde as respostas do sujeito).

	PONTOS		PONTOS
LUZ	0 1	QUADRO	0 1
SEDA	0 1	BICICLETA	0 1
AREIA	0 1	ESTRELA	0 1
CÍLIO	0 1	LEÃO	0 1
ARROZ	0 1	ANEL	0 1
GRAVATA	0 1	PERFUME	0 1

PONTUAÇÃO (2a. TENTATIVA): ____/12

(continuação)

"Agora, vou ler as mesmas palavras pela terceira vez e quero que se lembre da maior quantidade que puder se lembrar, inclusive as palavras que já foram ditas nas tentativas anteriores. Não importa a ordem das palavras, procure dizer todas elas incluindo aquelas que já foram recordadas na vez anterior. Pronto?".

	PONTOS			PONTOS	
LUZ	0	1	QUADRO	0	1
SEDA	0	1	BICICLETA	0	1
AREIA	0	1	ESTRELA	0	1
CÍLIO	0	1	LEÃO	0	1
ARROZ	0	1	ANEL	0	1
GRAVATA	0	1	PERFUME	0	1

PONTUAÇÃO (3a. TENTATIVA): _____/12

MAIOR PONTUAÇÃO ENTRE AS TRÊS TENTATIVAS: _____/12

2. NOMEAÇÃO POR CONFRONTO VISUAL

INSTRUÇÕES

Pede-se ao sujeito que nomeie os desenhos mostrados nos 20 cartões consecutivos. Não há tempo limite para a resposta, e apenas uma única tentativa é permitida. Nenhuma pista semântica ou fonêmica é oferecida. Quando os objetos são incluídos dentro de seu contexto (babador, fivela, crina, anzol, sino e casco), o avaliador pode indicar a parte do desenho a ser nomeado. Qualquer uma das palavras (sinônimas) descritas no quadro podem ser pontuadas.

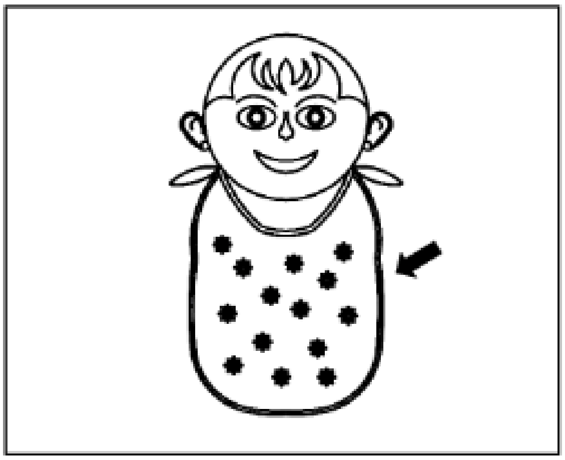

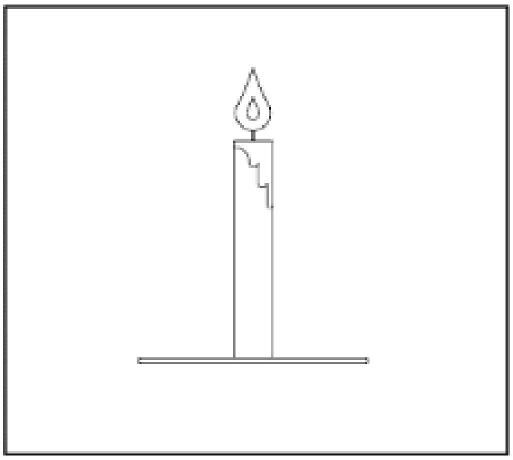

(continuação)

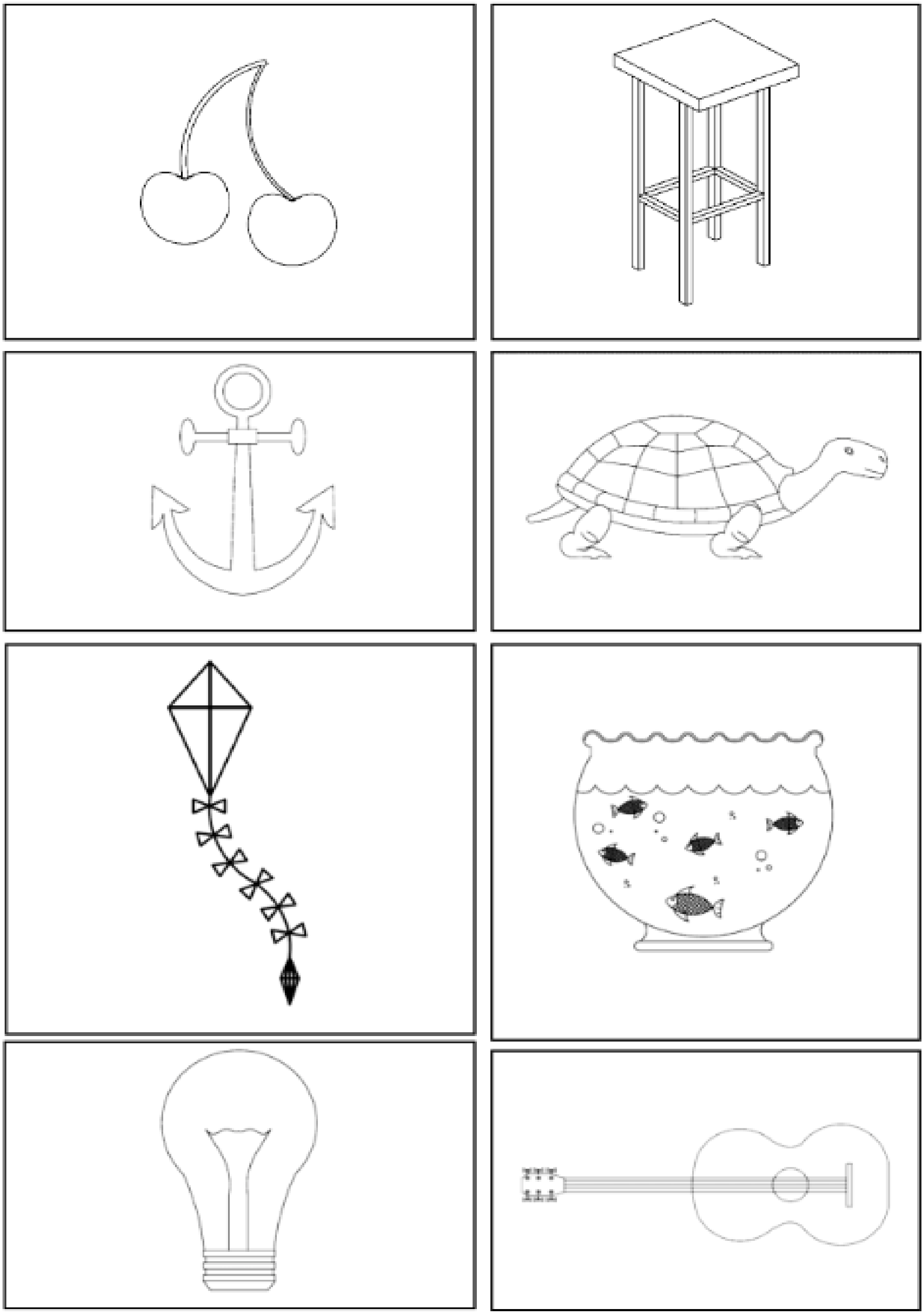

(continuação)

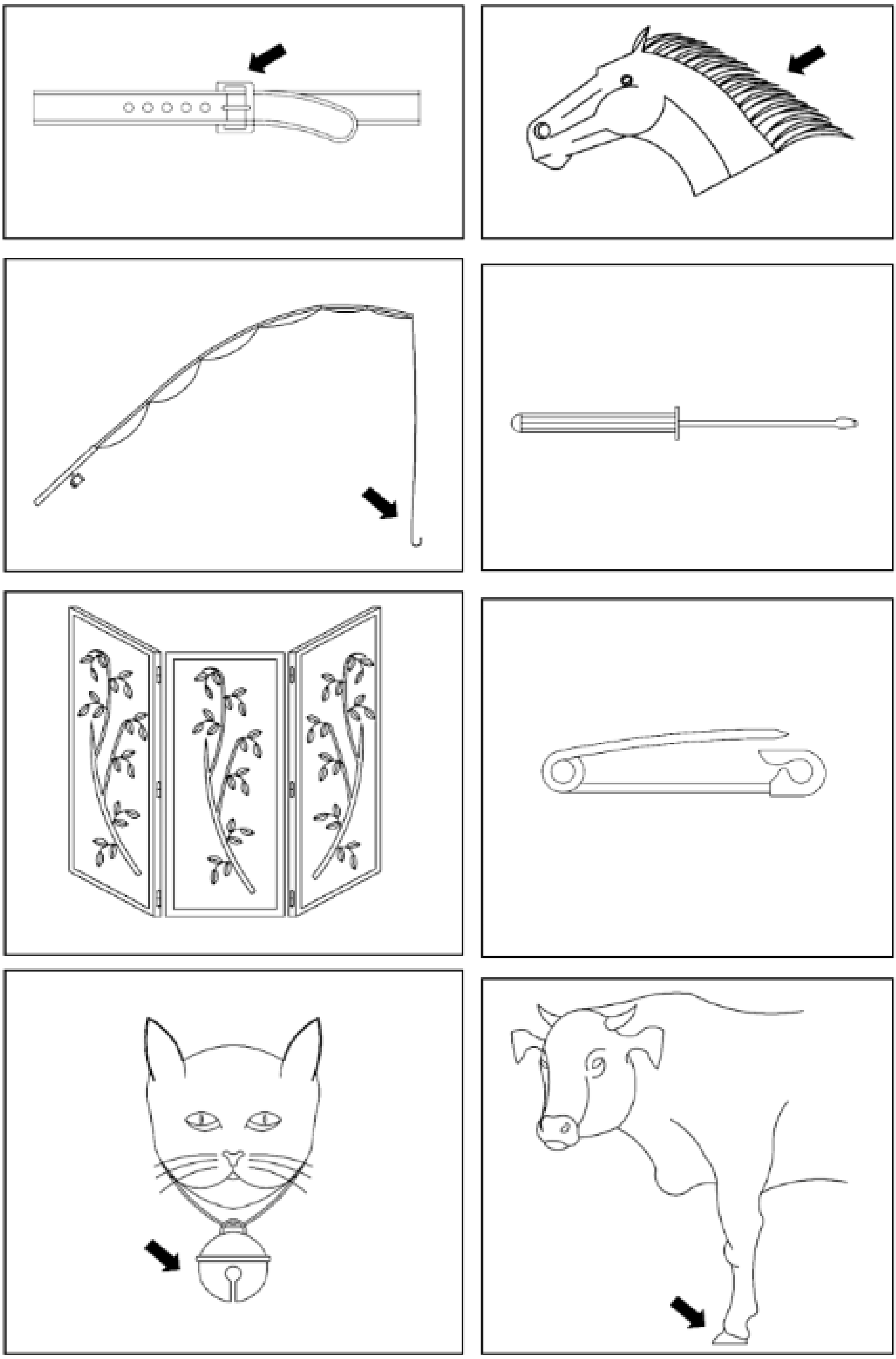

(continuação)

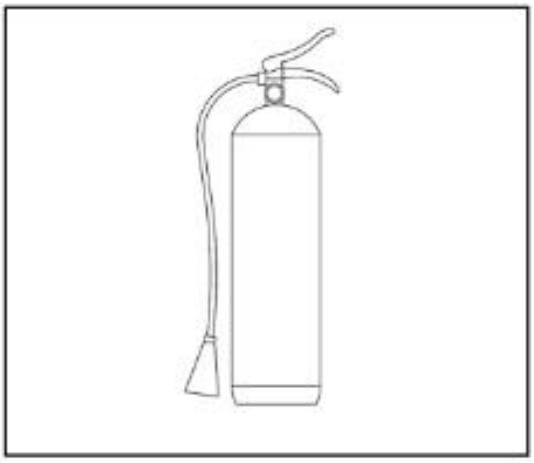

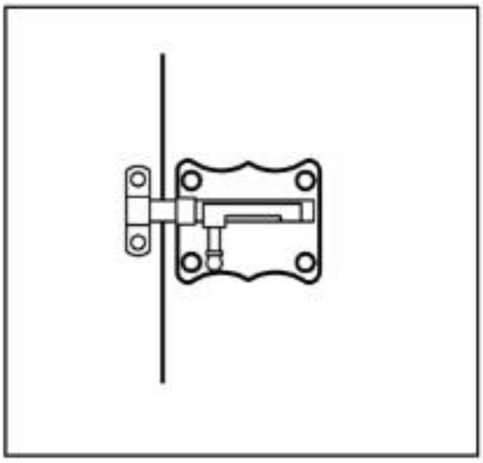

	PONTOS	
BABADOR	0	1
VELA	0	1
CEREJAS / MAÇÃS	0	1
BANQUETA / BANQUINHO / TAMBORETE / BANCO	0	1
ÂNCORA	0	1
TARTARUGA / JABOTI / CÁGADO / TRACAJÁ	0	1
PIPA / PAPAGAIO / ARRAIA	0	1
AQUÁRIO	0	1
LÂMPADA	0	1
VIOLÃO / VIOLA / GUITARRA	0	1
FIVELA	0	1
CRINA / JUBA	0	1
ANZOL	0	1
CHAVE DE FENDA	0	1
ANTEPARO / DIVISÓRIA / BIOMBO	0	1
ALFINETE	0	1
SINO / SININHO / GUIZO / MEDALHA DE IDENTIFICAÇÃO	0	1
CASCO / PATA	0	1
EXTINTOR	0	1
FECHADURA / TRINCO / FERROLHO	0	1

PONTUAÇÃO TOTAL: ____/20

(continuação)

3. ATENÇÃO SUSTENTADA

INSTRUÇÕES

Uma série ascendente de letras e números é lida para o sujeito. Pede-se que sujeito relate a quantidade de letras na sequência lida. Dez séries de letras e números são apresentadas, divididas em cinco níveis de complexidade crescente. Duas séries serão usadas, no início do teste, como treino. Diga ao sujeito: **"Eu vou ler uma sequência de números e letras. Quando eu acabar, quero que me diga quantas letras você ouviu. Preste bastante atenção, pois não posso repetir. Por exemplo, vou dizer 2 - L - T. Quantas letras tem?** (o sujeito responde 2 letras). **Muito bem! Se eu disser 8 - A - 9, quantas letras tem?** (1 letra). **Ok! Vamos começar?**".

TREINO (EXEMPLO)

	RESPOSTA CORRETA
2 L T	2 LETRAS
8 A 9	1 LETRA

TESTE

	RESPOSTA CORRETA	PONTOS
2 P 6 5 4	1 LETRA	0 1
3 A 6 K L	3 LETRAS	0 1
B 9 0 4 L T	3 LETRAS	0 1
3 C P 5 7 3	2 LETRAS	0 1
3 9 5 L 4 Z A	3 LETRAS	0 1
I 1 A S Q 4 1	4 LETRAS	0 1
7 5 D A 4 T B 2	4 LETRAS	0 1
9 6 8 4 3 7 L C	2 LETRAS	0 1
Z 4 9 A T D 3 8 4	4 LETRAS	0 1
9 5 M D 4 S C 3 E	5 LETRAS	0 1

PONTUAÇÃO TOTAL: ____/10

(continuação)

4. MEMÓRIA OPERACIONAL

INSTRUÇÕES

O examinador lê em voz alta uma lista randomizada de números e letras, variando em extensão de 2 a 6 letras e números. Após cada série, pede-se que o sujeito repita os números, em primeiro lugar e, então, as letras. Este teste termina quando o sujeito não for mais capaz de dar a resposta correta em duas séries consecutivas. Duas séries serão apresentadas, no início do teste, como treino. Diga ao sujeito: **"Vou ler uma lista de números e letras. Quando terminar, quero que você repita primeiro os números e depois as letras. Por exemplo, vou dizer L - 2 - T e o que você responde?** (o sujeito diz: 2 - L - T). **Muito bem! Mais uma vez: 8 - A - 9.** (o sujeito diz: 8 - 9 - A). **Muito bem! Podemos começar?"**

TREINO (EXEMPLO)

	RESPOSTA CORRETA
L 2 T →	2 L T
8 A 9 →	8 9 A

TESTE

	RESPOSTA CORRETA	PONTOS
M 3	3 M	0 1
7 P	7 P	0 1
G 8 M	8 G M	0 1
9 I 6	9 6 I	0 1
T 0 4 A	0 4 T A	0 1
7 V 6 J	7 6 V J	0 1
M 6 4 N I	6 4 M N I	0 1
3 5 S G C	3 5 S G C	0 1
1 R 9 V B 3	1 9 3 R V B	0 1
M 2 7 4 Z 9	2 7 4 9 M Z	0 1

PONTUAÇÃO TOTAL: ____/10

(continuação)

5.DESENHO DO RELÓGIO (ESPONTÂNEO)
**INSTRUÇÕES**<br>Pede-se que sujeito desenhe um relógio numa folha de papel em branco e colocar os ponteiros marcando "dez horas e vinte e cinco minutos" (0 a 10). Diga ao sujeito: "**Quero que você desenhe um relógio com todos os números, com os ponteiros marcando dez horas e vinte e cinco minutos. O relógio deve ter um contorno e os dois ponteiros devem ser desenhados na forma de setas. Pode começar.**".

(continuação)

5.DESENHO DO RELÓGIO (ESPONTÂNEO)

INSTRUÇÕES

Pede-se que sujeito desenhe um relógio numa folha de papel em branco e colocar os ponteiros marcando "dez horas e vinte e cinco minutos" (0 a 10). Diga ao sujeito: "**Quero que você desenhe um relógio com todos os números, com os ponteiros marcando dez horas e vinte e cinco minutos. O relógio deve ter um contorno e os dois ponteiros devem ser desenhados na forma de setas. Pode começar.**".

(continuação)

DESENHO ESPONTÂNEO DO RELÓGIO

	PONTOS	
A figura parece um relógio.	0	1
O relógio não é dividido por linhas ou setores.	0	1
Há uma disposição simétrica dos números.	0	1
Somente são escritos números no intervalo de 1 a 12.	0	1
Os números das horas estão corretamente sequenciados.	0	1
Somente dois ponteiros são desenhados.	0	1
Os ponteiros são representados como setas.	0	1
O ponteiro das horas é menor que o ponteiro dos minutos.	0	1
Nenhuma palavra foi escrita.	0	1
O número "25" não foi desenhado.	0	1

PONTUAÇÃO TOTAL: ____/10

CÓPIA DO DESENHO DO RELÓGIO

	PONTOS	
A figura parece um relógio.	0	1
O relógio não é dividido por linhas ou setores.	0	1
Há uma disposição simétrica dos números.	0	1
Somente são escritos números no intervalo de 1 a 12.	0	1
Os números das horas estão corretamente sequenciados.	0	1
Somente dois ponteiros são desenhados.	0	1
Os ponteiros são representados como setas.	0	1
O ponteiro das horas é menor que o ponteiro dos minutos.	0	1
Nenhuma palavra foi escrita.	0	1
O número "25" não foi desenhado.	0	1

PONTUAÇÃO TOTAL: ____/10

(continuação)

7. MEMÓRIA VERBAL DE EVOCAÇÃO LIVRE TARDIA

INSTRUÇÕES

Pede-se ao sujeito para se lembrar do maior número de palavras que puder da lista de palavras apresentadas no início do teste. Diga ao sujeito: **"Quero que você tente se lembrar daquela lista de palavras que foi lida três vezes para você no início deste teste. Tente se lembrar da maior quantidade de palavras que puder, em qualquer ordem".** Não forneça pistas aos pacientes.

	PONTOS			PONTOS	
LUZ	0	1	QUADRO	0	1
SEDA	0	1	BICICLETA	0	1
AREIA	0	1	ESTRELA	0	1
CÍLIO	0	1	LEÃO	0	1
ARROZ	0	1	ANEL	0	1
GRAVATA	0	1	PERFUME	0	1

PONTUAÇÃO: _____/12

7. FLUÊNCIA VERBAL ALTERNADA

INSTRUÇÕES

Pede-se ao sujeito para gerar o maior número de palavras possíveis que comecem com a letra 'S' de maneira alternada com a maior quantidade possível de "itens do vestuário" durante 60 segundos. Os participantes são instruídos a não usarem nomes próprios ou repetir a mesma palavra com terminação diferente (ex.: sapo, sapa, sapinho). Diga ao sujeito: **"Durante um minuto, quero que me diga a maior quantidade de palavras que comecem com a letra 'S', de maneira alternada com a maior quantidade possível de itens do vestuário. Você irá dizer algo que comece com a letra 'S' e em seguida uma vestimenta. Depois outra palavra com 'S' e outra vestimenta. Lembre-se que as vestimentas podem começar com qualquer letra. Não use nomes próprios. Fará isto o mais rapidamente que puder por um minuto. Podemos começar?"**. (Marque 60 segundos no relógio).

REGRA DE PONTUAÇÃO:

1 ponto para cada resposta correta que mantenha a alternância entre as palavras iniciadas com 'S' e um "item do vestuário" (0 a 20).

(continuação)

PALAVRAS INICIADAS POR "S"	VESTIMENTA

PONTUAÇÃO: _____/20

7. FLUÊNCIA VERBAL DE AÇÕES

INSTRUÇÕES

Leia para o sujeito: **"Durante 60 segundos, quero que me diga o maior número de coisas diferentes que você puder pensar que as pessoas fazem. Eu não quero que você use a mesma palavra com diferentes terminações, como "comer, comendo, comido". Também, me diga apenas palavras como "comer", ou "cheirar", no lugar de uma frase ou sentença (ex. indo comer). Quero somente as palavras, entendeu? Podemos começar?".**

REGRA DE PONTUAÇÃO:

1 ponto para cada resposta correta (0 a 30).

PALAVRAS QUE REPRESENTAM "AÇÕES" ("COISAS QUE AS PESSOAS FAZEM")

PONTUAÇÃO: _____/30

(continuação)

TABELA DE CORREÇÃO DOS TESTES	PONTUAÇÃO
MEMÓRIA VERBAL IMEDIATA	____/12
NOMEAÇÃO POR CONFRONTO VISUAL	____/20
ATENÇÃO SUSTENTADA	____/10
MEMÓRIA OPERACIONAL	____/10
DESENHO DO RELÓGIO	____/10
CÓPIA DO RELÓGIO	____/10
MEMÓRIA VERBAL TARDIA	____/12
FLUÊNCIA VERBAL ALTERNADA	____/20
FLUÊNCIA VERBAL DE AÇÕES	____/30
**FRONTAL-SUBCORTICAL**	____/104
**CORTICAL POSTERIOR**	____/30
**TOTAL PD-CRS**	____/134

Observação:
O escore **Cortical Posterior** é composto pela soma dos itens sublinhados.
O escore F**rontal-Subcortical** é composto pela soma dos demais itens.

ANEXO 8 - MEDIDA CANADENSE DE TERAPIA OCUPACIONAL

MEDIDA CANADENSE DE DESEMPENHO OCUPACIONAL (COPM)[1]

Segunda Edição

Autores: Mary Law, Sue Baptiste, Anne Carswell, Mary Ann McColl, Helene Polatajko, Nancy Pollock[2]

Nome do cliente: ______ Idade: ______ Sexo: ______

Entrevistado: ______ Registro nº: ______

(se não for o cliente)

Data da avaliação: ______

Terapeuta: ______

Data prevista para reavaliação: ______

Clínica/Hospital: ______ Programa: ______

Data da reavaliação: ______

PASSO 1: IDENTIFICAÇÃO DE QUESTÕES NO DESEMPENHO OCUPACIONAL

Para identificar problemas, preocupações e questões relativas ao desempenho ocupacional, entreviste o cliente questionando sobre as atividades do dia-a-dia no que se refere às atividades produtivas, de autocuidado e de lazer. Solicite ao cliente que identifique as atividades do dia-a-dia que quer realizar, que necessita realizar ou que é esperado que ele realize, encorajando-o a pensar num dia típico. Em seguida, peça que identifique quais dessas atividades atualmente são difíceis de realizar, de forma satisfatória. Registre estas atividades problemáticas nos Passos 1A, 1B ou 1C.

PASSO 2: CLASSIFICAÇÃO DO GRAU DE IMPORTÂNCIA

Usando os cartões de pontuação, peça ao cliente que classifique, numa escala de 1 a 10, a importância de cada atividade. Coloque as pontuações nos respectivos quadrados nos Passos 1A, 1B e 1C.

A. Autocuidado		Importância
**Cuidados pessoais**	______	☐
(ex.: vestuário, banho,	______	☐
alimentação, higiene)	______	☐
**Mobilidade funcional:**	______	☐
(ex.: transferências, mobilidade	______	☐
dentro e fora de casa)	______	☐
**Independência fora de casa:**	______	☐
(ex.: transportes, compras, finanças)	______	☐
	______	☐
**B. Produtividade**		**Importância**
**Trabalho (remunerado/não-remunerado)**	______	☐
(ex.: procurar/manter um emprego,	______	☐
atividades voluntárias)	______	☐
**Tarefas domésticas**	______	☐
(ex.: limpezas, lavagem de roupas,	______	☐
preparação de refeições)	______	☐
**Brincar/Escola**	______	☐
(ex.: habilidade para brincar,	______	☐
fazer o dever de casa)	______	☐
**C. Lazer**		**Importância**
**Recreação tranqüila**	______	☐
(ex.: *hobbies*, leitura, artesanato)	______	☐
	______	☐
**Recreação ativa**	______	☐
(ex.: esportes, passeios, viagens)	______	☐
	______	☐
**Socialização**	______	☐
(ex.: visitas, telefonemas,	______	☐
festas, escrever cartas)	______	☐

[1] *Canadian Occupational Performance Measure (COPM)*. Versão brasileira traduzida por Lívia C. Magalhães, Lílian V. Magalhães e Ana Amélia Cardoso.

[2] Publicado pela CAOT Publications ACE © M. Law, S. Baptiste, A. Carswell, M. A. McColl, H. Polatajko, N. Pollock, 2000

(continuação)

PASSO 3: PONTUAÇÃO – AVALIAÇÃO INICIAL

Confirme com o cliente os 5 problemas mais importantes e registre-os abaixo. Usando os cartões de pontuação, peça ao cliente para classificar cada problema no que diz respeito ao Desempenho e Satisfação, depois calcule a pontuação total. Para calcular a pontuação total some a pontuação do desempenho ocupacional ou da satisfação de todos os problemas e divida pelo número de problemas.

PASSO 4: REAVALIAÇÃO

No intervalo de tempo apropriado para reavaliação, o cliente classifica novamente cada problema, no que se refere ao Desempenho e à Satisfação.

Problemas de Desempenho Ocupacional	Avaliação Inicial		Reavaliação	
	Desempenho 1	Satisfação 1	Desempenho 2	Satisfação 2
1.				
2.				
3.				
4.				
5.				

Problemas de Desempenho Ocupacional	Pontuação do Desempenho 1	Pontuação da Satisfação 1	Pontuação do Desempenho 2	Pontuação da Satisfação 2
Pontuação Total = Pontuação Total do Desempenho ou da Satisfação / Nº de Problemas	___ / ___ = ___	___ / ___ = ___	___ / ___ = ___	___ / ___ = ___

PASSO 5: COMPUTANDO OS ESCORES DE MUDANÇA

Calcule as mudanças, subtraindo a pontuação obtida na avaliação da obtida na reavaliação.

Mudança no Desempenho = Pontuação do Desempenho 2 ___ – Pontuação do Desempenho 1 ___ = ___

Mudança na Satisfação = Pontuação da Satisfação 2 ___ – Pontuação da Satisfação 1 ___ = ___

ANOTAÇÕES ADICIONAIS E OBSERVAÇÕES

Avaliação inicial:

Reavaliação:

[1] *Canadian Occupational Performance Measure* (COPM). Versão brasileira traduzida por Lívia C. Magalhães, Lilian V. Magalhães e Ana Amélia Cardoso.

[2] Publicado pela CAOT Publications ACE © M. Law, S. Baptiste, A. Carswell, M. A. McColl, H. Polatajko, N. Pollock, 2000
