# Supplementary material for: The use of brain-machine interface, motor imagery, and action observation in the rehabilitation of individuals with Parkinson’s disease: A protocol study for a randomized clinical trial
Source: PLoS One. 2025 Apr 7;20(4):e0315148. doi: 10.1371/journal.pone.0315148 (PMC11975075; doi:10.1371/journal.pone.0315148)
Supplement: Attachment — Submitted filename: PLOS 1.docx [file pone.0315148.s011.docx]

The article is really well written, and it doesn’t almost need formal changes. I’ve only a very few formal criticisms: Authors underlined too frequently the fact that their protocol is very detailed, and it is the first one to be presented in a so detailed manner. One time is enough, I think.

Table 1: Open medication bottle and the following tasks with medications. Why instructions for AO and MI are different as far as the use of dominant and not dominant hand is concerned? This would make AO and MI not comparable. Moreover, the description seems to me contradictory to the statement at page 26, lines 407-8.

Page 27, lines 448-450 “Recruitment based on media outreach and contact with municipal health departments and associations as an enrollment strategy to reach the target sample size.” It seems to me that some words are lacking in this sentence.

On the other hand, I’d like to express a few ideas of mine about the protocol content. The Authors are not obliged to change the protocol according to my ideas, but I’d like to give them some suggestions to think of, and maybe better motivate their choices in the text.

Selection of participants: “Additionally, those assigned to the BMI groups must not be using deep brain stimulation (DBS) devices.” This should be true for all groups. The presence of a DBS device or not represents a great difference between participants with PD for a lot of reasons.

Evaluation of participants: ““How would you rate the severity of your cognitive functions (memory, attention, concentration, organization”. Are the Authors sure that the patients will make a difference between attention and concentration? What’s the difference between attention and concentration according to the Authors?

Experimental treatment, motor imagery: “The activities will be narrated by the therapist during the intervention, guiding the participant - eyes closed and seated - to imagine executing the action from a first-person perspective, without the presence of motor symptoms”. How are the Authors sure that the participants affected by PD will be able to imagine the movement without symptoms? Maybe they should at least ask the participant whether they succeeded or not.

It's really a great limitation the fact that the follow-up evaluation will be performed only by phone and only using the COPM. Why did the Authors choose not to re-examining the enrolled participants in flesh?
