# Supplementary material for: The use of brain-machine interface, motor imagery, and action observation in the rehabilitation of individuals with Parkinson’s disease: A protocol study for a randomized clinical trial
Source: PLoS One. 2025 Apr 7;20(4):e0315148. doi: 10.1371/journal.pone.0315148 (PMC11975075; doi:10.1371/journal.pone.0315148)
Supplement: Attachment — Submitted filename: Response to Reviewers.docx [file pone.0315148.s013.docx]

**Response to Reviewers**

Dear Academic Editor and Reviewers,

In response to your guidance, I address each point raised for corrections to the article. I would like to thank you in advance for your valuable contributions to improving the protocol.

**For Reviewer #1:**

a) We have revised the statistical method as suggested, where the False Discovery Rate (FDR) will be used for multiple comparisons among the five groups (lines 462).
b) The choice of GEE is to avoid losing potential cases in the event of follow-up loss. The goodness of fit will be assessed using the Quasi-Likelihood under Independence Model Criterion (QIC) (lines 467-468).
c) We agree with your comments and have included the suggested information as the last sentence of the article's conclusion (page 32, lines 568-571): “The expected results and findings from this study are representative only of this sample, and future trials with larger sample sizes and conducted in other geographical locations will be necessary to assess the effectiveness of the interventions.”

**For Reviewer #2:**

- I have edited the text to avoid repetition regarding the description of the protocol by removing the word "detailed" in the objective section of the abstract (line 34), in the last paragraph of the introduction (line 105), and in the first paragraph of the study design section in the methods (line 112).
- **Table 1 (pages 17-19):**

The verbal command directed at the participant during MI is correct according to the images. However, there was an error in the technical description of the movements performed during AO, which has now been corrected in the article. We appreciate the observation. It is worth noting that the technical description is not provided to the participant, as they only observe the videos without any additional stimuli. This description is exclusively for therapists when creating the videos, ensuring proper consideration of the movements for each activity stage. Although the person in the video is right-handed, bimanual activities can be performed with either the dominant or non-dominant hand, while unilateral activities are carried out using the side where motor symptoms are present. Therapists adjust the MI guidance accordingly, instructing participants to use either their right or left hand depending on the side affected by symptoms, using accessible language.

- **Page 27, lines 448-450:**

I have revised the sentence to make it complete, including the types of media used for research dissemination (lines 453-455).

- **Selection of participants:**

I have corrected the selection criteria (page 7, lines 157-158), specifying that DBS was considered an exclusion criterion for all groups, precisely for the reason mentioned.

- **Evaluation of participants:**

The intention is to illustrate cognitive functions (memory, attention, concentration, organization), as participants may not be familiar with the concept of cognition or cognitive functions. However, since the assessment does not focus on any specific function but rather evaluates a general state, we do not believe this presents an issue. Nonetheless, we can incorporate real-life examples, such as:

- “If you are watching television and someone asks you for a favor, can you focus only on the television or the request? Can you remember the favor afterward, or do you forget it quickly?”
- “Is it easier for you to remember what you need to buy at the market or memorize someone’s phone number?”
- **Experimental treatment, motor imagery:**

Since we will use the *Kinesthetic and Visual Imagery Questionnaire (KVIQ-10)* during screening, we can already evaluate the participant's imagery ability, including image clarity and sensation intensity. However, we agree it is essential to ask participants afterward whether they were able to imagine themselves, and we have added this information to the protocol (page 12, lines 274-277).

- We removed the section where we mentioned conducting the COPM by phone as a limitation of the study (lines560-563). The COPM is a semi-structured interview in which individuals assess their own perceptions of changes in occupational performance. This format allows for remote application, making it more convenient for participants by eliminating the need for an additional in-person meeting for data collection.

I would like to highlight that I have corrected the formatting of the figures (Fig 1 and Fig 2), placing the captions below the titles (pages 7-8).
